# Supplementary material for: Impact of chronic oral glucocorticoid treatment on mortality in patients with COVID-19: analysis of a population-based cohort
Source: BMJ Open. 2024 Mar 15;14(3):e080640. doi: 10.1136/bmjopen-2023-080640 (PMC10946357; doi:10.1136/bmjopen-2023-080640)
Supplement: Supplementary data [file bmjopen-2023-080640supp001.pdf]

Supplementary appendix

Supplementary Table S1: **Dose equivalents used in the calculation of equivalence to prednisolone............ 2**

Supplementary Table S2: **Covariates selected in the evaluation of the effect of glucocorticoid treatment on death in COVID-19 patients in the Swedish population-based cohort of COVID-19 patients from COVID-19 infection date to Nov 30, 2021.......... 3**

Supplementary Table S3: **Outcome events in individuals with COVID-19 infection in the Swedish population during the period 2015 to COVID-19 infection date until Nov 30, 2021 stratified by level of exposure to oral glucocorticoids (non-exposed, high exposure in the 6 months before infection, or any exposure 12 months before infection and overall).......... 5**

Supplementary Table S4: **Baseline characteristics of individuals with COVID-19 infection in the Swedish population during the period 2015 to COVID-19 infection date (comorbidities) or 1 year before COVID-19 infection date (medications) until Nov 30, 2021, stratified by level of exposure to oral glucocorticoids (high exposure in the 6 months before infection or any exposure 12 months before infection), with standardized mean differences between exposed and 1:2 propensity score matched non-exposed 6**

Supplementary Table S5: **Outcome events in propensity score matched cohorts of individuals with COVID-19 infection in the Swedish population during the period 2015 to COVID-19 infection date until Nov 30, 2021 stratified by level of exposure to oral glucocorticoids (non-exposed, high exposure in the 6 months before infection, or any exposure 12 months before infection and overall).......... 8**

Supplementary Table S6: **Standardised mean differences between the 15 a priori selected covariates of individuals with COVID-19 infections in the Swedish population during the period 2015 to COVID-19 infection date (comorbidities) or 1 year before COVID-19 infection date (medications) in those exposed to oral glucocorticoids compared with those non-exposed before and after propensity score matching, main analysis.......... 9**

Supplementary Table S7: **Standardised mean differences between the 15 a priori selected plus added‡ covariates in sensitivity analysis of individuals with COVID-19 infections in the Swedish population during the period 2015 to COVID-19 infection date (comorbidities) or 1 year before COVID-19 infection date (medications) in those exposed to oral glucocorticoids compared with those non-exposed before and after propensity score matching.......... 10**

Supplementary Table S8: **Unadjusted, as well as adjusted from main and sensitivity analysis of the risk of various causes of death and all-cause mortality between COVID-19 patients who had any or high prior exposure to oral glucocorticoids and non-exposed individuals, hazard ratios from Cox regression analyses and propensity score matched analysis with 95% confidence intervals.......... 11**

Supplementary Figure S1: **Propensity score balance before (a, c) and after (b, d) performing propensity score matching on all 15 covariates for individuals with COVID-19 infections in the Swedish population between 2015 and the COVID-19 infection date comparing those exposed to oral glucocorticoids (pink) with those who were non-exposed (blue) in those with any (a, b) or high glucocorticoid exposure (c, d).. 13**

Supplementary Figure S2: Standardised mean differences between the covariates of individuals with COVID-19 infections in the Swedish population during the period 2015 to COVID-19 infection date (comorbidities) or 1 year before COVID-19 infection date (medications) in the exposed to oral glucocorticoids compared with non-exposed before (red circles) and after (blue triangles) propensity score matching among those with (a) any or (b) high glucocorticoid exposure. 14

Einarsdottir MJ, *et al. BMJ Open* 2024; 14:e080640. doi: 10.1136/bmjopen-2023-080640

Supplementary Table S1: **Dose equivalents used in the calculation of equivalence to prednisolone**

ATC code	Recommended international non-proprietary drug name	Equivalent dose to 1 mg of prednisolone (mg)	Equivalent dose to 750 mg of prednisolone (mg)*
H02AB01	Betamethasone	8·333	6250·0
H02AB02	Dexamethasone	6·666	5000·0
H02AB04	Methylprednisolone	1·25	937·5
H02AB06	Prednisolone	1	750·0
H02AB07	Prednisone	1	750·0
H02AB09	Hydrocortisone	0·25	187·5
H02AB10	Cortisone	0·2	150·0
H02AB13	Deflazacort	0·666	500·0

ATC, Anatomical Therapeutic Chemical. *The definition of high oral glucocorticoid exposure was ≥2 prescriptions of prednisolone ≥750 mg or equivalent within 6 months before COVID-19 infection date. The corresponding equivalent amount to prednisolone 750 mg or the other oral glucocorticoids is shown on this column.

Einarsdottir MJ, *et al. BMJ Open* 2024; 14:e080640. doi: 10.1136/bmjopen-2023-080640

Supplementary Table S2: **Covariates selected in the evaluation of the effect of glucocorticoid treatment on death in COVID-19 patients in the Swedish population-based cohort of COVID-19 patients from COVID-19 infection date to Nov 30, 2021**

**Covariate**	**Units and categories**	**Register source; description**	**Lookback window before COVID-19 infection date (index date)**
Sociodemographic characteristics			
Age	As a continuous variable and in 10-year categories	RTB	At Jan 1, 2020
Sex	Male, female	RTB	At Jan 1, 2020
Education	Primary, secondary, higher, or unknown	LISA: primary education included pre-secondary education <9, 9, or >9 years. Secondary educated included 2-year high school education, secondary education <3 years or >3 years, and higher education included those with post-high school education. Unknown includes children with no education information or individuals with no relevant education (eg, immigrants).	At Jan 1, 2020
Employment status	Employed, unemployed, or unknown	LISA: created using the variable "employment" and stratified by age where: "employed" was either employed or with entrepreneurial income, and "unemployed" was the unemployed and without entrepreneurial income and includes pensioners. Unknown includes individuals with no information on employment.	At Jan 1, 2020
Comorbidity	**Units/categories**	**NPR: ICD-10 codes, any mention of condition as primary or secondary diagnosis for a hospitalisation or ≥1 visit at any time**	Jan 1, 2015 to index date
Pulmonary embolism	Yes/No	NPR: I26	Jan 1, 2015 to index date
Stroke	Yes/No	NPR: I61 I63 I649 I691 I693 I694 I698	Jan 1, 2015 to index date
Deep vein thrombosis	Yes/No	NPR: I80–I82	Jan 1, 2015 to index date
Hypertension	Yes/No	NPR: I10–I15	Jan 1, 2015 to index date
Ischaemic heart disease	Yes/No	NPR: I20 –I25	Jan 1, 2015 to index date
Overall cardiovascular disease	Yes/No	NPR: I00–I99	Jan 1, 2015 to index date
Heart failure	Yes/No	NPR: I50	Jan 1, 2015 to index date
Asthma	Yes/No	NPR: J45	Jan 1, 2015 to index date
COPD	Yes/No	NPR: J44	Jan 1, 2015 to index date
Emphysema	Yes/No	NPR: J43	Jan 1, 2015 to index date
Diabetes	Yes/No	NPR: E10, E11, E13, E14	Jan 1, 2015 to index date
Rheumatic disease	Yes/No	NPR: M05, M06, M31, M35	Jan 1, 2015 to index date
Cancer	Yes/No	NPR: C00–C97	Jan 1, 2015 to index date
Covariates for sensitivity analysis*			
Adrenal insufficiency	Yes/No	NPR: E271, E272, E230, E230A, E230B, E230C, E230D, E230E, E230F, E230W, E230X, E893, E310	Jan 1, 2015 to index date
Rheumatoid arthritis	Yes/No	NPR: M050-M053, M058, M058A, M058B, M058C, M058D, M058F, M058G, M058H, M058L, M058M, M058N, M058X, M059, M059L, M059M, M059N, M06-P, M060, M060L, M060M, M060N, M061-M064, M068, M068L, M068M, M068N, M069, M069A, M069B, M069C, M069D, M069F, M069G, M069H, M069X	Jan 1, 2015 to index date
Chronic lower respiratory diseases	Yes/No	NPR: J41-J47	Jan 1, 2015 to index date
Other respiratory diseases principally affecting the interstitium	Yes/No	NPR: J80-J84	Jan 1, 2015 to index date
Non-infective enteritis and colitis,	Yes/No	NPR: K50-K52	Jan 1, 2015 to index date

Einarsdottir MJ, *et al. BMJ Open* 2024; 14:e080640. doi: 10.1136/bmjopen-2023-080640

Dermatitis and eczema	Yes/No	NPR:L20-L30	Jan 1, 2015 to index date
Inflammatory polyarthropathies	Yes/No	NPR: M05-M14	Jan 1, 2015 to index date
Systemic connective tissue disorders (except RA M31 and M35)	Yes/No	NPR: M30-M36 (excluding M35.3, M31.5 and M31.6)	Jan 1, 2015 to index date
Glomerular diseases,	Yes/No	NPR: N01-N08	Jan 1, 2015 to index date
Organ or tissue transplant	Yes/No	NPR: Z940-Z944, Z948	Jan 1, 2015 to index date
Chronic liver disease	Yes/No	NPR: K72-K75	Jan 1, 2015 to index date
Alcoholism and other substance abuse	Yes/No	NPR: F10-F19	Jan 1, 2015 to index date
Prescribed medications	**Units/categories**	**NPDR: ATC codes. At least one filled prescription in lookback window**	
Inhaled corticosteroids*	Yes/No	R03BA, R03AK, R03AL08, R03AL09	1 year before index
Oral anticoagulants	Yes/No	B01AA, B01AF	1 year before index
Statins*	Yes/No	C10AA	1 year before index
ACEIs*	Yes/No	C09A	1 year before index
Opioids*	Yes/No	N02AB	1 year before index

ACEIs, angiotensin-converting enzyme inhibitors; ATC, Anatomical Therapeutic Chemical; BMI, body mass index; COPD, chronic obstructive pulmonary disease; ICD-10, International Classification of Diseases, version 10; LISA, Longitudinal Integrated Database for Health Insurance and Labor Market Studies; NPDR, Swedish National Prescribed Drug Register; NPR, Swedish National Patient Register; RTB, Register of Total Population; SNAR, Swedish National Airways Register; *16 covariates for sensitivity analyses.

Einarsdottir MJ, *et al. BMJ Open* 2024; 14:e080640. doi: 10.1136/bmjopen-2023-080640

Supplementary Table S3: **Outcome events in individuals with COVID-19 infection in the Swedish population during the period 2015 to COVID-19 infection date until Nov 30, 2021 stratified by level of exposure to oral glucocorticoids (non-exposed, high exposure in the 6 months before infection, or any exposure 12 months before infection and overall)**

	No=0/Yes=1	Non-exposed (n=1 151 347)	Any GC exposure* (n=48 806)	High GC exposure† (n=13 497)	Total study population (n=1 200 153)
All-cause mortality					
	0	1 136 497 (98·7%)	45 428 (93·1%)	11 474 (85·0%)	1 181 925 (98·5%)
	1	14 850 (1·3%)	3378 (6·9%)	2023 (15·0%)	18 228 (1·5%)
Death from pulmonary embolism					
	0	1 150 769 (99·9%)	48 654 (99·7%)	13 417 (99·4%)	1 199 423 (99·9%)
	1	578 (0·1%)	152 (0·3%)	80 (0·6%)	730 (0·1%)
Death from sepsis					
	0	1 150 576 (99·9%)	48 520 (99·4%)	13 335 (98·8%)	1 199 096 (99·9%)
	1	771 (0·1%)	286 (0·6%)	162 (1·2%)	1057 (0·1%)
Death from myocardial infarction					
	0	1 150 755 (99·9%)	48 707 (99·8%)	13 440 (99·6%)	1 199 462 (99·9%)
	1	592 (0·1%)	99 (0·2%)	57 (0·4%)	691 (0·1%)
Death from stroke					
	0	1 150 152 (99·9%)	48 625 (99·6%)	13 396 (99·3%)	1 198 777 (99·9%)
	1	1195 (0·1%)	181 (0·4%)	101 (0·7%)	1376 (0·1%)
Death from COVID-19					
	0	1 137 876 (98·8%)	45 781 (93·8%)	11 678 (86·5%)	1 183 657 (98·6%)
	1	13 471 (1·2%)	3025 (6·2%)	1819 (13·5%)	16 496 (1·4%)
Hospitalisation for COVID-19					
	0	1 088 788 (94·6%)	38 383 (78·6%)	8460 (62·7%)	1 127 171 (93·9%)
	1	62 559 (5·4%)	10 423 (21·4%)	5037 (37·3%)	72 982 (6·1%)
ICU admission for COVID-19					
	0	1 144 510 (99·4%)	47 747 (97·8%)	13 004 (96·3%)	1 192 257 (99·3%)
	1	6837 (0·6%)	1059 (2·2%)	493 (3·7%)	7896 (0·7%)

GC, glucocorticoid; ICU, intensive care unit. *One or more prescriptions of oral GCs within 12 months before the COVID-19 infection date. †Two or more prescriptions and prednisolone ≥750 mg or equivalent within 6 months before COVID-19 infection date. Note this group is a subset of any prior exposure.

Einarsdottir MJ, *et al. BMJ Open* 2024; 14:e080640. doi: 10.1136/bmjopen-2023-080640

BMJ Publishing Group Limited (BMJ) disclaims all liability and responsibility arising from any reliance placed on this supplemental material which has been supplied by the author(s)

Supplementary Table S4: **Baseline characteristics of individuals with COVID-19 infection in the Swedish population during the period 2015 to COVID-19 infection date (comorbidities) or 1 year before COVID-19 infection date (medications) until Nov 30, 2021, stratified by level of exposure to oral glucocorticoids (high exposure in the 6 months before infection or any exposure 12 months before infection), with standardized mean differences between exposed and 1:2 propensity score matched non-exposed**

	Any prior GC exposure* (n=47 737)	Non-exposed matched to any prior exposure (n=91 878)	SMD (any prior *vs* matched non-exposed)	High GC exposure† (n=12 943)	Non-exposed matched to the high GC exposed (n=23 898)	SMD (high *vs* matched non-exposed)
Age (years)	50·83 (21·06)	49·95 (20·83)	0·042	61·60 (19·69)	60·17 (19·63)	0·073
Age category (years)			0·047			0·081
0–9	930 (1·9)	1840 (2·0)		91 (0·7)	174 (0·7)	
10–19	3056 (6·4)	6089 (6·6)		284 (2·2)	556 (2·3)	
20–29	4491 (9·4)	8982 (9·8)		567 (4·4)	1128 (4·7)	
30–39	5879 (12·3)	11 699 (12·7)		925 (7·1)	1834 (7·7)	
40–49	8041 (16·8)	15 882 (17·3)		1514 (11·7)	2940 (12·3)	
50–59	8953 (18·8)	17 620 (19·2)		2237 (17·3)	4481 (18·8)	
60–69	6221 (13·0)	11 784 (12·8)		2146 (16·6)	4095 (17·1)	
70–79	5321 (11·1)	9652 (10·5)		2515 (19·4)	4403 (18·4)	
80–89	3719 (7·8)	6374 (6·9)		2021 (15·6)	3243 (13·6)	
≥90	1126 (2·4)	1956 (2·1)		643 (5·0)	1044 (4·4)	
Sex			0·002			0·021
Male	20 132 (42·2)	38 844 (42·3)		5480 (42·3)	10 368 (43·4)	
Female	27 605 (57·8)	53 034 (57·7)		7463 (57·7)	13 530 (56·6)	
Birth country			0·087			0·137
Sweden	37 607 (78·8)	69 317 (75·4)		10 450 (80·7)	18 124 (75·8)	
Nordic	1223 (2·6)	2201 (2·4)		456 (3·5)	756 (3·2)	
Europe except Nordics	3084 (6·5)	6953 (7·6)		744 (5·7)	1768 (7·4)	
Other	5823 (12·2)	13 407 (14·6)		1293 (10·0)	3250 (13·6)	
Employment status			0·036			0·053
Employed	31 069 (65·1)	61 146 (66·6)		6398 (49·4)	12 421 (52·0)	
Unemployed	14 422 (30·2)	26 260 (28·6)		6337 (49·0)	11 073 (46·3)	
Unknown	2246 (4·7)	4472 (4·9)		208 (1·6)	404 (1·7)	
Education level			0·018			0·018
Primary	9397 (19·7)	17 445 (19·0)		3408 (26·3)	6114 (25·6)	
Secondary	19 470 (40·8)	37 752 (41·1)		5466 (42·2)	10 160 (42·5)	
Higher	15 699 (32·9)	30 487 (33·2)		3628 (28·0)	6819 (28·5)	
Unknown	3171 (6·6)	6194 (6·7)		441 (3·4)	805 (3·4)	
Comorbidities						
Pulmonary embolism	1536 (3·2)	2643 (2·9)	0·020	768 (5·9)	1243 (5·2)	0·032
Stroke	1606 (3·4)	2780 (3·0)	0·019	793 (6·1)	1366 (5·7)	0·017
Deep vein thrombosis	278 (0·6)	542 (0·6)	0·001	87 (0·7)	154 (0·6)	0·003
Hypertension	12 570 (26·3)	23 230 (25·3)	0·024	5753 (44·4)	10 133 (42·4)	0·041
Ischaemic heart disease	4054 (8·5)	7209 (7·8)	0·024	1907 (14·7)	3201 (13·4)	0·039
Overall CV disease	18 019 (37·7)	31 289 (34·1)	0·077	7751 (59·9)	12 982 (54·3)	0·113
Heart failure	3989 (8·4)	7060 (7·7)	0·025	2033 (15·7)	3442 (14·4)	0·036
Asthma	7625 (16·0)	4507 (4·9)	0·368	1935 (15·0)	1591 (6·7)	0·270
COPD	3403 (7·1)	6444 (7·0)	0·004	1500 (11·6)	2769 (11·6)	0·001

Einarsdottir MJ, *et al. BMJ Open* 2024; 14:e080640. doi: 10.1136/bmjopen-2023-080640

Emphysema	354 (0·7)	404 (0·4)	0·039	185 (1·4)	190 (0·8)	0·061
Diabetes mellitus	5098 (10·7)	9671 (10·5)	0·005	2431 (18·8)	4336 (18·1)	0·016
Rheumatic disease	5523 (11·6)	7075 (7·7)	0·131	3557 (27·5)	4843 (20·3)	0·170
Cancer	7171 (15·0)	13 624 (14·8)	0·005	3219 (24·9)	6235 (26·1)	0·028
Prescribed medications						
Inhaled corticosteroids	17 363 (36·4)	14 069 (15·3)	0·496	4061 (31·4)	4602 (19·3)	0·281
Oral anticoagulants	8017 (16·8)	14 677 (16·0)	0·022	3617 (27·9)	6288 (26·3)	0·037
Statins	10 571 (22·1)	19 209 (20·9)	0·030	4240 (32·8)	7728 (32·3)	0·009
ACEIs	6601 (13·8)	12 781 (13·9)	0·002	2658 (20·5)	5130 (21·5)	0·023
Opioids	12 026 (25·2)	14 517 (15·8)	0·234	4284 (33·1)	5064 (21·2)	0·270

Data are n (%) or mean (SD). ACEI, angiotensin-converting enzyme inhibitor; COPD, chronic obstructive pulmonary disease; CV, cardiovascular; GC, glucocorticoid; SMD, standardised mean difference.
*One or more prescriptions of oral GCs within 12 months before the COVID-19 infection date. †Two or more prescriptions and prednisolone ≥750 mg or equivalent within 6 months before COVID-19 infection date. Note this group is a subset of any prior exposure.

Supplementary Table S5: **Outcome events in propensity score matched cohorts of individuals with COVID-19 infection in the Swedish population during the period 2015 to COVID-19 infection date until Nov 30, 2021 stratified by level of exposure to oral glucocorticoids (non-exposed, high exposure in the 6 months before infection, or any exposure 12 months before infection and overall)**

	No=0/Yes=1	Any GC exposure and matched non-exposed			High GC exposure and matched non-exposed		
		Any GC exposure* (n=47 737)	Matched non-exposed (n=91 878)	Total any exposure and matched non-exposed (n=139 615)	High GC exposure† (n=12 943)	Matched non-exposed (n=23 898)	Total high exposure and matched non-exposed (n=36 841)
All-cause mortality							
	0	44 573 (93·4%)	87 973 (95·7%)	132 546 (94·9%)	11 054 (85·4%)	22 024 (92·2%)	33 078 (89·8%)
	1	3164 (6·6%)	3905 (4·3%)	7069 (5·1%)	1889 (14·6%)	1874 (7·8%)	3763 (10·2%)
Death from pulmonary embolism							
	0	47 598 (99·7%)	91 691 (99·8%)	139 289 (99·8%)	12 867 (99·4%)	23 804 (99·6%)	36 671 (99·5%)
	1	139 (0·3%)	187 (0·2%)	326 (0·2%)	76 (0·6%)	94 (0·4%)	170 (0·5%)
Death from sepsis							
	0	47 467 (99·4%)	91 627 (99·7%)	139 094 (99·6%)	12 799 (98·9%)	23 786 (99·5%)	36 585 (99·3%)
	1	270 (0·6%)	251 (0·3%)	521 (0·4%)	144 (1·1%)	112 (0·5%)	256 (0·7%)
Death from myocardial infarction							
	0	47 646 (99·8%)	91 718 (99·8%)	139 364 (99·8%)	12 889 (99·6%)	23 830 (99·7%)	36 719 (99·7%)
	1	91 (0·2%)	160 (0·2%)	251 (0·2%)	54 (0·4%)	68 (0·3%)	122 (0·3%)
Death from stroke							
	0	475 63 (99·6%)	91 592 (99·7%)	139 155 (99·7%)	12 845 (99·2%)	23 752 (99·4%)	36 597 (99·3%)
	1	174 (0·4%)	286 (0·3%)	460 (0·3%)	98 (0·8%)	146 (0·6%)	244 (0·7%)
Death from COVID-19							
	0	44 904 (94·1%)	88 387 (96·2%)	133 291 (95·5%)	11 240 (86·8%)	22 219 (93·0%)	33459 (90·8%)
	1	2833 (5·9%)	3491 (3·8%)	6324 (4·5%)	1703 (13·2%)	1679 (7·0%)	3382 (9·2%)
Hospitalisation for COVID-19							
	0	37 981 (79·6%)	77 727 (84·6%)	115 708 (82·9%)	8252 (63·8%)	17 810 (74·5%)	26 062 (70·7%)
	1	9756 (20·4%)	14 151 (15·4%)	23 907 (17·1%)	4691 (36·2%)	6088 (25·5%)	10 779 (29·3%)
ICU admission for COVID-19							
	0	46 736 (97·9%)	90 383 (98·4%)	137 119 (98·2%)	12 470 (96·3%)	23 320 (97·6%)	35 790 (97·1%)
	1	1001 (2·1%)	1495 (1·6%)	2496 (1·8%)	473 (3·7%)	578 (2·4%)	1051 (2·9%)

GC, glucocorticoid; ICU, intensive care unit. *One or more prescriptions of oral GCs within 12 months before the COVID-19 infection date. †Two or more prescriptions and prednisolone ≥750 mg of or equivalent within 6 months before COVID-19 infection date. Note this group is a subset of any prior exposure.

Einarsdottir MJ, *et al. BMJ Open* 2024; 14:e080640. doi: 10.1136/bmjopen-2023-080640

Supplementary Table S6: **Standardised mean differences between the 15 a priori selected covariates of individuals with COVID-19 infections in the Swedish population during the period 2015 to COVID-19 infection date (comorbidities) or 1 year before COVID-19 infection date (medications) in those exposed to oral glucocorticoids compared with those non-exposed before and after propensity score matching, main analysis**

**Variable**	**Any prior GC exposure***		**High GC exposure†**	
	**SMD before PS matching**	**SMD after PS matching**	**SMD before PS matching**	**SMD after PS matching**
Age categories	0·672	0·047	1·276	0·081
Sex	0·149	0·002	0·147	0·021
Education	0·300	0·018	0·501	0·018
Employment	0·453	0·036	0·888	0·053
Pulmonary embolism	0·194	0·020	0·310	0·032
Stroke	0·161	0·019	0·282	0·017
Deep vein thrombosis	0·088	0·001	0·098	0·003
Hypertension	0·547	0·024	0·972	0·041
Ischaemic heart disease	0·292	0·024	0·487	0·039
Heart failure	0·341	0·025	0·561	0·036
COPD	0·356	0·004	0·488	0·001
Diabetes mellitus	0·289	0·005	0·510	0·016
Rheumatic disease	0·510	0·131	0·897	0·170
Cancer	0·406	0·005	0·653	0·028
Oral anticoagulants	0·413	0·022	0·689	0·037

COPD, chronic obstructive pulmonary disease; GC, glucocorticoid; PS, propensity score; SMD, standardised mean difference. *One or more prescriptions of oral GCs within 12 months before the COVID-19 infection date. †Two or more prescriptions and a total of prednisolone ≥750 mg or equivalent within 6 months before COVID-19 infection date. Note this group is a subset of any prior exposure.

Supplementary Table S7: **Standardised mean differences between the 15 a priori selected plus added‡ covariates in sensitivity analysis of individuals with COVID-19 infections in the Swedish population during the period 2015 to COVID-19 infection date (comorbidities) or 1 year before COVID-19 infection date (medications) in those exposed to oral glucocorticoids compared with those non-exposed before and after propensity score matching**

Variable	Any prior GC exposure*		High GC exposure†	
	SMD before PS matching	SMD after PS matching	SMD before PS matching	SMD after PS matching
Age categories	0.672	0.052	1.276	0.046
Sex	0.149	0.006	0.147	0.019
Birth country	0.105	0.065	0.199	0.092
Employment status	0.453	0.048	0.888	0.035
Education level	0.300	0.023	0.501	0.017
Pulmonary embolism	0.194	0.034	0.310	0.026
Stroke	0.161	0.026	0.282	0.013
Deep vein thrombosis	0.088	0.005	0.098	0.006
Hypertension	0.547	0.053	0.972	0.045
Ischaemic heart disease	0.292	0.043	0.487	0.027
Heart failure	0.341	0.060	0.561	0.048
COPD	0.357	0.047	0.488	0.006
Diabetes mellitus	0·289	0.068	0·510	0.105
Rheumatic disease	0·510	0.039	0·897	0.021
Cancer	0·406	0.151	0·653	0.185
Oral anticoagulants	0.413	0.046	0.689	0.035
Added covariates‡				
Adrenal insufficiency	0.168	0.031	0.285	0.023
Rheumatoid arthritis	0.344	0.086	0.539	0.078
Chronic lower respiratory diseases	0.559	0.009	0.618	0.043
Other respiratory diseases principally affecting the interstitium	0.195	0.037	0.308	0.031
Non-infective enteritis and colitis,	0.255	0.020	0.322	0.056
Dermatitis and eczema	0.262	0.016	0.287	0.023
Inflammatory polyarthropathies	0.471	0.072	0.718	0.049
Systemic connective tissue disorders (except rheumatoid arthritis ICD-coded M31 and M35)	0.234	0.044	0.362	0.029
Glomerular diseases,	0.160	0.048	0.247	0.068
Chronic liver disease	0.140	0.070	0.213	0.009
Organ or tissue transplant	0.234	0.107	0.358	0.111
Alcoholism and other substance abuse	0.118	0.014	0.156	0.004
Opioids	0.424	0.016	0.601	0.008
Inhaled corticosteroids	0.645	0.059	0.535	0.071
Statins	0.389	0.027	0.640	0.014
ACEIs	0.283	0.016	0.460	0.001

COPD, chronic obstructive pulmonary disease; GC, glucocorticoid; PS, propensity score; SMD, standardised mean difference; ICD, 10th edition of the International Classification of Diseases; ACEIs, angiotensin converting enzyme inhibitors. *One or more prescriptions of oral GCs within 12 months before the COVID-19 infection date. †Two or more prescriptions and a total of prednisolone ≥750 mg or equivalent within 6 months before COVID-19 infection date. Note this group is a subset of any prior exposure. ‡ 16 other covariates of interest for oral GC users.

Supplementary Table S8: **Unadjusted, as well as adjusted from main and sensitivity analysis of the risk of various causes of death and all-cause mortality between COVID-19 patients who had any or high prior exposure to oral glucocorticoids and non-exposed individuals, hazard ratios from Cox regression analyses and propensity score matched analysis with 95% confidence intervals.**

**Outcomes and method**	**Any prior GC exposure***		**High GC exposure†**	
	**HR (95% CI)**	**No. of events**	**HR (95% CI)**	**No. of events**
All-cause mortality				
Unadjusted Cox regression	5·46 (5·26–5·67)	18 228	12·33 (11·77–12·92)	16 873
Adjusted Cox regression‡	1·58 (1·52–1·65)	18 228	1·98 (1·87–2·09)	16 873
Cox with extended adjustments‡‡ [Sensitivity analysis]	1.55 [1.48-1.61]	18 228	1.85 [1.75-1.96]	16 873
PS matching¶	1·47 (1·40–1·54)	7 069	1·83 (1·71–1·95)	3 763
PS matching with additional covariates¶¶[Sensitivity analysis]	1.53 [1.46-1.61]	6 673	1.67 [1.57-1.79]	3 886
Death from pulmonary embolism				
Unadjusted Cox regression	6·29 (5·26–7·52)	730	12·68 (10·04–16·03)	658
Adjusted Cox regression‡	1·33 (1·11–1·59)	730	1·37 (1·09–1·74)	658
Cox with extended adjustments‡‡ [Sensitivity analysis]	1.38 [1.16-1.65]	730	1.35 [1.07-1.71]	658
PS matching¶	1·32 (1·05–1·65)	326	1·52 (1·12–2·06)	170
PS matching with additional covariates¶¶[Sensitivity analysis]	1.54 [1.21-1.95]	290	1.52 [1.11-2.09]	160
Death from sepsis				
Unadjusted Cox regression	8·67 (7·57–9·94)	1 057	18·72 (15·79–22·19)	933
Adjusted Cox regression‡	2·35 (2·01–2·74)	1 057	2·90 (2·37–3·54)	933
Cox with extended adjustments‡‡ [Sensitivity analysis]	2.10 [1.79-2.47]	1 057	2.43 [1.96-3.00]	933
PS matching¶	1·92 (1·61–2·29)	521	2·40 (1·86–3·09)	256
PS matching with additional covariates¶¶[Sensitivity analysis]	1.87 [1.56-2.25]	492	1.90 [1.49-2.42]	276
Death from stroke				
Unadjusted Cox regression	3·47 (2·96–4·05)	1 376	7·22 (5·89–8·85)	1 296
Adjusted Cox regression‡	1·16 (0·98–1·37)	1 376	1·33 (1·05–1·67)	1 296
Cox with extended adjustments‡‡ [Sensitivity analysis]	1.14 [0.96-1.36]	1 376	1.23 [0.97-1.56]	1 296
PS matching¶	1·08 (0·89–1·31)	460	1·23 (0·94–1·59)	244
PS matching with additional covariates¶¶[Sensitivity analysis]	1.01 [0.83-1.24]	443	0.99 [0.76-1.28]	263
Death from myocardial infarction				
Unadjusted Cox regression	3·84 (3·10–4·75)	691	8·23 (6·27–10·82)	649
Adjusted Cox regression‡	1·04 (0·82–1·31)	691	1·36 (1·00–1·86)	649
Cox with extended adjustments‡‡ [Sensitivity analysis]	1.08 [0.85-1.38]	691	1.41 [1.02-1.94]	649
PS matching¶	1·02 (0·79–1·33)	251	1·48 (1·03–2·13)	122
PS matching with additional covariates¶¶[Sensitivity analysis]	1.05 [0.80-1.37]	239	1.24 [0.86-1.78]	125
Death from COVID–19				
Unadjusted Cox regression	5·43 (5·22–5·65)	16 496	12·34 (11·75–12·96)	15 290
Adjusted Cox regression‡	1·57 (1·50–1·64)	16 496	1·96 (1·85–2·08)	15 290
Cox with extended adjustments‡‡ [Sensitivity analysis]	1.53 [1.46-1.60]	16 496	1.83 [1.73-1.94]	15 290
PS matching¶	1·48 (1·41–1·56)	6 324	1·84 (1·72–1·97)	3 382
PS matching with additional covariates¶¶[Sensitivity analysis]	1.54 [1.46-1.62]	5 989	1.69 [1.58-1.81]	3 489
Secondary outcomes				
Hospitalisation for COVID–19				
Unadjusted Cox regression	4·34 (4·25–4·43)	72 982	8·52 (8·28–8·77)	67 596
Adjusted Cox regression‡	1·42 (1·39–1·45)	72 982	1·51 (1·46–1·57)	67 596
Cox with extended adjustments‡‡ [Sensitivity analysis]	1.20 [1.17-1.23]	72 982	1.25 [1.20-1.29]	67 596
PS matching¶	1·28 (1·25–1·31)	23 907	1·44 (1·38–1·49)	10 779

PS matching with additional covariates[¶¶][Sensitivity analysis]	1.19 [1.16-1.22]	23 814	1.29 [1.24-1.34]	11 037
ICU admission for COVID–19				
Unadjusted Cox regression	3·72 (3·49–3·97)	7 896	6·41 (5·85–7·03)	7 330
Adjusted Cox regression[‡]	1·33 (1·24–1·43)	7 896	1·40 (1·26–1·55)	7 330
Cox with extended adjustments[‡‡] [Sensitivity analysis]	0.90 [0.84-0.97]	7 896	0.80 [0.72-0.89]	7 330
PS matching[¶]	1·24 (1·15–1·35)	2 496	1·52 (1·34–1·72)	1 051
PS matching with additional covariates[¶¶][Sensitivity analysis]	0.99 [0.92-1.07]	2 703	1.00 [0.89-1.13]	1 206

CI, confidence interval; GC, glucocorticoid; HR, hazard ratio; PS, propensity score. *One or more prescriptions of oral GCs within 12 months before the COVID-19 infection date. [†]Two or more prescriptions and prednisolone ≥750 mg or equivalent within 6 months before COVID-19 infection date. Note high exposure is a subset of any prior exposure. [‡]Adjusted for initial a priori selected covariates: age, sex, education, employment, and previous medical history (diabetes, deep vein thrombosis, pulmonary embolism, hypertension, stroke, ischaemic heart disease, heart failure, cancer, chronic obstructive pulmonary disease, rheumatologic conditions, and oral anticoagulation therapy. [‡‡] Adjusted for additional covariates on top of initial (initial covariates + adrenal insufficiency, rheumatoid arthritis, chronic lower respiratory diseases, other respiratory diseases principally affecting the interstitium, non-infective enteritis and colitis, dermatitis and eczema, inflammatory polyarthropathies, systemic connective tissue disorders (except rheumatoid arthritis), glomerular diseases, organ or tissue transplant , chronic liver disease, medications: inhaled corticosteroids, statins, aceis, opioids, alcoholism and other substance abuse). [¶]1:2 matched on a PS score based on 15 initial a priori selected covariates: age, sex, education, employment, and previous medical history (diabetes, deep vein thrombosis, pulmonary embolism, hypertension, stroke, ischaemic heart disease, heart failure, cancer, chronic obstructive pulmonary disease, rheumatologic conditions, oral anticoagulation therapy). [¶¶] 1:2 matched on a PS score based on 31 covariates: initial 15 + adrenal insufficiency, rheumatoid arthritis, chronic lower respiratory diseases, other respiratory diseases principally affecting the interstitium, non-infective enteritis and colitis, dermatitis and eczema, inflammatory polyarthropathies, systemic connective tissue disorders (except rheumatoid arthritis), glomerular diseases, organ or tissue transplant , chronic liver disease, medications: inhaled corticosteroids, statins, aceis, opioids, alcoholism and other substance abuse.

Einarsdottir MJ, *et al. BMJ Open* 2024; 14:e080640. doi: 10.1136/bmjopen-2023-080640

Supplementary Figure S1: **Propensity score balance before (a, c) and after (b, d) performing propensity score matching on all 15 covariates for individuals with COVID-19 infections in the Swedish population between 2015 and the COVID-19 infection date comparing those exposed to oral glucocorticoids (pink) with those who were non-exposed (blue) in those with any (a, b) or high glucocorticoid exposure (c, d)**

GC, glucocorticoid; PS, propensity score. Any prior GC exposure equals ≥1 prescription of oral GC within 12 months before the COVID-19 infection date. High GC exposure equals ≥2 prescriptions with a total of ≥750 mg of prednisolone or equivalent. The latter group is a subset of any prior exposure. Note: The figure includes PS density plots and histograms illustrating the distribution of PS values. Prior to matching, the PS density plots and histograms represent the distribution of PS values for both exposed and non-exposed individuals. Subsequently, a 1:2 matching scheme was employed using a caliper of 0.2 where each exposed individual was paired with two non-exposed individuals based on their similarity in PS values. The PS density plots and histograms after matching reflect the distribution of PS values for the matched groups, demonstrating the achieved balance in the covariates. The 15 covariates used in the logistic model to estimate the propensity score can be found in Supplementary Table S7.

(a) Any GC exposure before PS matching

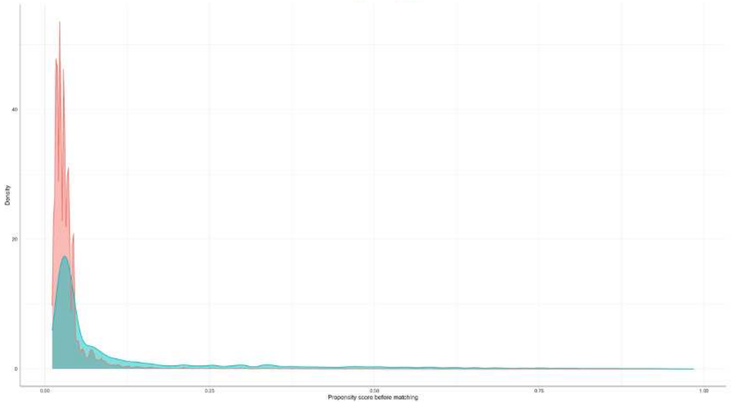

(b) Any GC exposure after PS matching

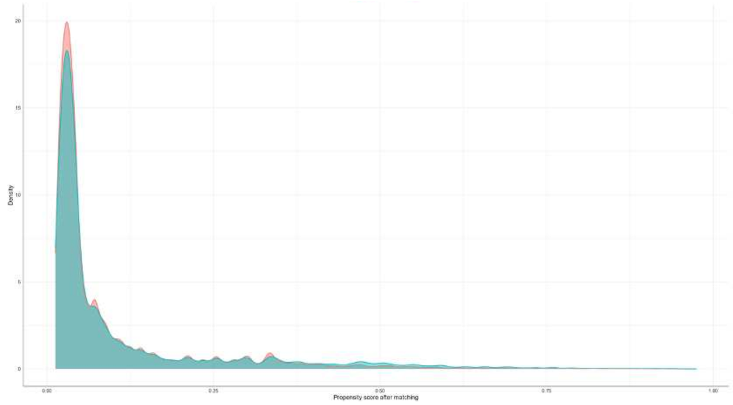

(c) High GC exposure before PS matching

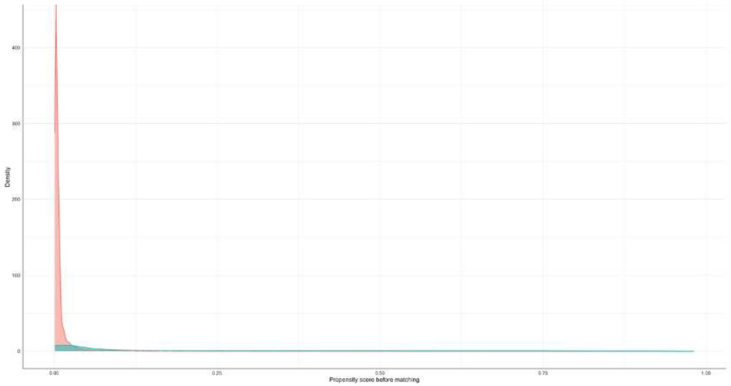

(d) High GC exposure after PS matching

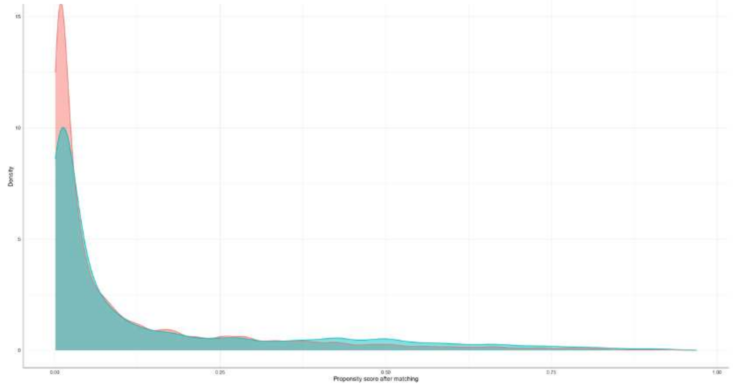

Supplementary Figure S2: Standardised mean differences between the covariates of individuals with COVID-19 infections in the Swedish population during the period 2015 to COVID-19 infection date (comorbidities) or 1 year before COVID-19 infection date (medications) in the exposed to oral glucocorticoids compared with non-exposed before (red circles) and after (blue triangles) propensity score matching among those with (a) any or (b) high glucocorticoid exposure. COPD, chronic obstructive pulmonary disease; GC, glucocorticoid. Any prior exposure equals ≥1 prescription of oral glucocorticoids within 12 months before the COVID-19 infection date. High exposure equals ≥2 prescriptions of oral glucocorticoids with a total of ≥750 mg of prednisolone or equivalent within 6 months before COVID-19 infection date. Note the latter group is a subset of any prior exposure.

(a) Any GC exposure

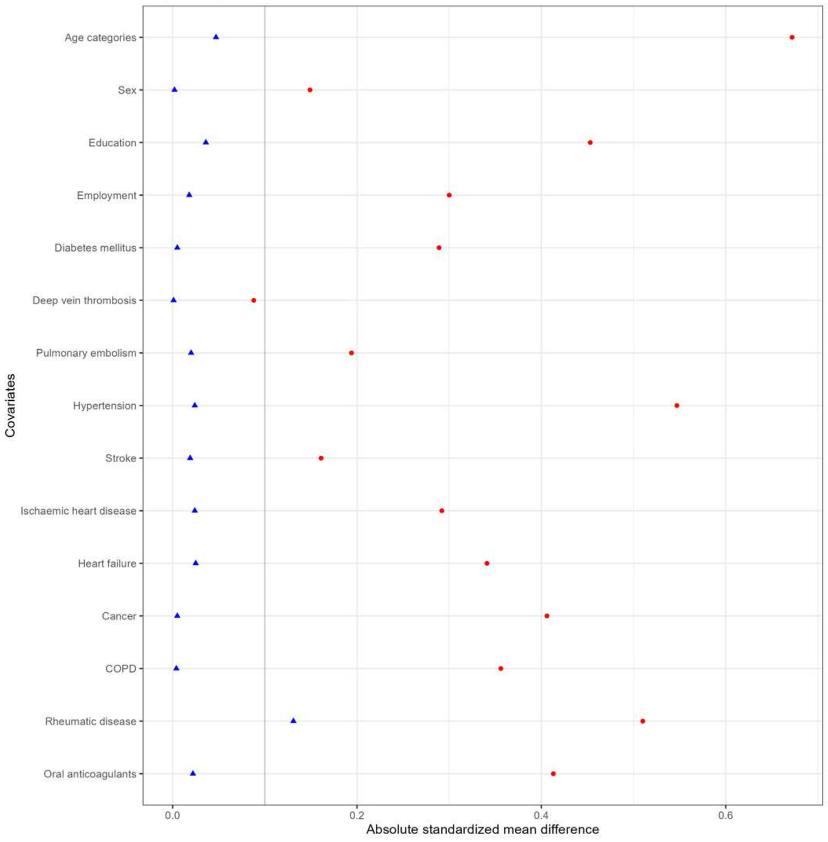

(b) High GC exposure

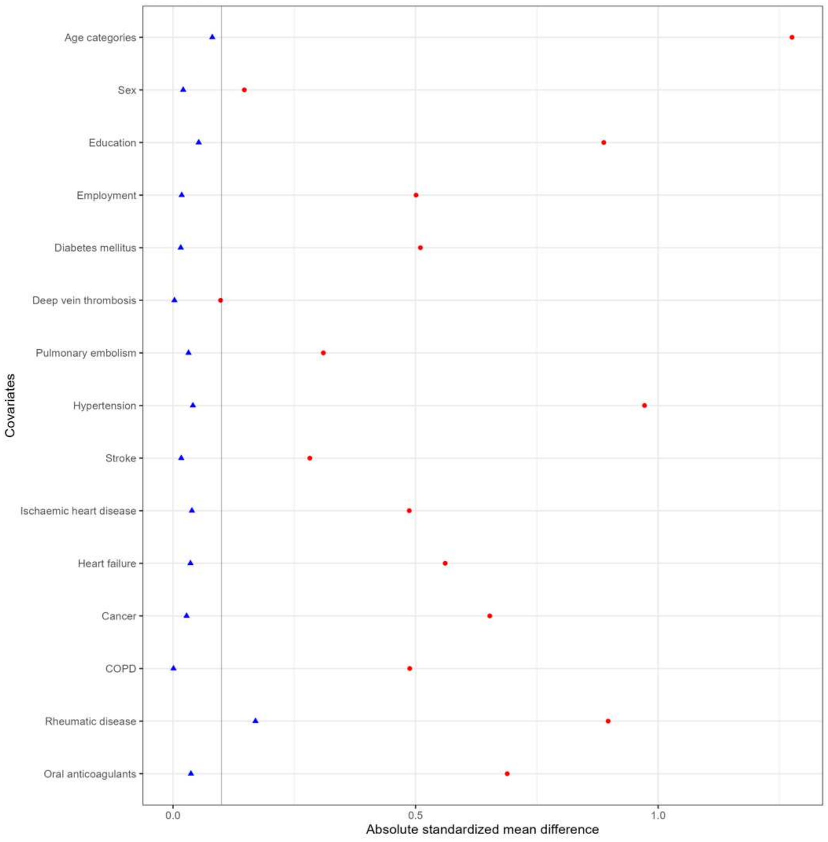

Einarsdottir MJ, *et al. BMJ Open* 2024; 14:e080640. doi: 10.1136/bmjopen-2023-080640
