# Supplementary material for: Impact of chronic oral glucocorticoid treatment on mortality in patients with COVID-19: analysis of a population-based cohort
Source: BMJ Open. 2024 Mar 15;14(3):e080640. doi: 10.1136/bmjopen-2023-080640 (PMC10946357; doi:10.1136/bmjopen-2023-080640)
Supplement: Author's manuscript [file bmjopen-2023-080640.draft_revisions.pdf]

BMJ Open is committed to open peer review. As part of this commitment we make the peer review history of every article we publish publicly available.

When an article is published we post the peer reviewers' comments and the authors' responses online. We also post the versions of the paper that were used during peer review. These are the versions that the peer review comments apply to.

The versions of the paper that follow are the versions that were submitted during the peer review process. They are not the versions of record or the final published versions. They should not be cited or distributed as the published version of this manuscript.

BMJ Open is an open access journal and the full, final, typeset and author-corrected version of record of the manuscript is available on our site with no access controls, subscription charges or pay-per-view fees (http://bmjopen.bmj.com).

If you have any questions on BMJ Open's open peer review process please email info.bmjopen@bmj.com

BMJ Open

Impact of chronic oral glucocorticoid treatment on mortality in patients with COVID-19: analysis of a population-based cohort

Journal:	*BMJ Open*
Manuscript ID	bmjopen-2023-080640
Article Type:	Original research
Date Submitted by the Author:	06-Oct-2023

Complete List of Authors:	Einarsdottir, Margret; University of Gothenburg, Department of Internal Medicine and Clinical Nutrition; Sahlgrenska University Hospital, Department of Endocrinology<br>Kibiwott Kirui, Brian; University of Gothenburg, School of Public Health and Community Medicine, Institute of Medicine, Sahlgrenska Academy<br>Li, Huiqi; University of Gothenburg School of Public Health and Community Medicine<br>Olsson, Daniel; University of Gothenburg, Department of Internal Medicine and Clinical Nutrition; AstraZeneca<br>Johannsson, Gudmundur; University of Gothenburg, Department of Internal Medicine and Clinical Nutrition; Sahlgrenska University Hospital, Department of Endocrinology<br>Nyberg, Fredrik; University of Gothenburg School of Public Health and Community Medicine<br>Ragnarsson, Oskar; University of Gothenburg, Department of Internal Medicine and Clinical Nutrition; Sahlgrenska University Hospital, Department of Endocrinology

Keywords:	Mortality, COVID-19, Adrenal disorders < DIABETES & ENDOCRINOLOGY

SCHOLARONE™ Manuscripts

I, the Submitting Author has the right to grant and does grant on behalf of all authors of the Work (as defined in the below author licence), an exclusive licence and/or a non-exclusive licence for contributions from authors who are: i) UK Crown employees; ii) where BMJ has agreed a CC-BY licence shall apply, and/or iii) in accordance with the terms applicable for US Federal Government officers or employees acting as part of their official duties; on a worldwide, perpetual, irrevocable, royalty-free basis to BMJ Publishing Group Ltd ("BMJ") its licensees and where the relevant Journal is co-owned by BMJ to the co-owners of the Journal, to publish the Work in this journal and any other BMJ products and to exploit all rights, as set out in our licence.

The Submitting Author accepts and understands that any supply made under these terms is made by BMJ to the Submitting Author unless you are acting as an employee on behalf of your employer or a postgraduate student of an affiliated institution which is paying any applicable article publishing charge ("APC") for Open Access articles. Where the Submitting Author wishes to make the Work available on an Open Access basis (and intends to pay the relevant APC), the terms of reuse of such Open Access shall be governed by a Creative Commons licence – details of these licences and which Creative Commons licence will apply to this Work are set out in our licence referred to above.

Other than as permitted in any relevant BMJ Author's Self Archiving Policies, I confirm this Work has not been accepted for publication elsewhere, is not being considered for publication elsewhere and does not duplicate material already published. I confirm all authors consent to publication of this Work and authorise the granting of this licence.

For peer review only

Impact of chronic oral glucocorticoid treatment on mortality in patients with COVID-19: analysis of a population-based cohort

Running title: Glucocorticoids, mortality and COVID-19

Authors: Margrét J Einarsdóttir (0000-0002-6010-9599)[1,2]*, Brian K Kirui (0000-0002-6656-6029)[3]*, Huiqi Li (0000-0002-1127-0829) [3], Daniel S Olsson (0000-0002-9734-0786)[1,2,5], Gudmundur Johannsson (0000-0003-3484-8440)[1,2], Fredrik Nyberg (0000-0003-0892-5668)[3†], Oskar Ragnarsson (0000-0003-0204-9492)[1,2,4†]

*Joint first authors

†Joint senior authors

Affiliations: [1]Department of Internal Medicine and Clinical Nutrition, Institute of Medicine, Sahlgrenska Academy, University of Gothenburg, Gothenburg, Sweden; [2]Department of Endocrinology, Sahlgrenska University Hospital, Gothenburg, Sweden; [3]School of Public Health and Community Medicine, Institute of Medicine, Sahlgrenska Academy, University of Gothenburg, Gothenburg, Sweden; [4]Wallenberg Center for Molecular and Translational Medicine, University of Gothenburg, Gothenburg, Sweden; [5]Cardiovascular, Renal and Metabolism (CVRM), BioPharmaceuticals R&D, AstraZeneca, Gothenburg, Sweden.

Address for correspondence and reprint requests: Oskar Ragnarsson, Department of Internal Medicine and Clinical Nutrition, Institute of Medicine,

For peer review only

Sahlgrenska Academy, University of Gothenburg, 405 30 Gothenburg, Sweden; Tel: +46 70 728 2229; E-mail: oskar.ragnarsson@medic.gu.se

Manuscript category: Original article

Abstract length: 240

Text length: 2929

No. of references: 29

No. of graphics: 4 (1 table and 3 figures)

Supplementary data: Appendix provided as separate file

Author addresses:

Margrét J Einarsdóttir, Department of Internal Medicine and Clinical Nutrition, Institute of Medicine, Sahlgrenska Academy, University of Gothenburg, 405 30 Gothenburg, Sweden; Tel: +46313427056; E-mail: margret.jona.einarsdottir@gu.se

Brian K Kirui, School of Public Health and Community Medicine, Institute of Medicine, Sahlgrenska Academy, University of Gothenburg, Gothenburg, Sweden; Tel: +46700579203; E-mail: brian.kirui@gu.se

Huiqi Li, School of Public Health and Community Medicine, Institute of Medicine, Sahlgrenska Academy, University of Gothenburg, Gothenburg, Sweden; Tel: +46317866251; E-mail: huiqi.li@gu.se

For peer review only

Daniel S Olsson, Department of Internal Medicine and Clinical Nutrition, Institute of Medicine, Sahlgrenska Academy, University of Gothenburg, 405 30 Gothenburg, Sweden; Tel: +46708278671; E-mail: daniel.olsson@gu.se

Gudmundur Johannsson, Department of Internal Medicine and Clinical Nutrition, Institute of Medicine, Sahlgrenska Academy, University of Gothenburg, 405 30 Gothenburg, Sweden; Tel: +46313427052 E-mail: gudmundur.johannsson@medic.gu.se

Fredrik Nyberg, School of Public Health and Community Medicine, Institute of Medicine, Sahlgrenska Academy, University of Gothenburg, Gothenburg, Sweden; Tel: +46 31 7866289; E-mail: fredrik.nyberg.2@gu.se

Oskar Ragnarsson, Department of Internal Medicine and Clinical Nutrition, Institute of Medicine, Sahlgrenska Academy, University of Gothenburg, 405 30 Gothenburg, Sweden; Tel: +46 70 728 2229; E-mail: oskar.ragnarsson@medic.gu.se

For peer review only

Data availability statement

The individual level data used in this study are pseudonymised and sourced from Swedish healthcare registers. Interested researchers can obtain access to the data from the appropriate Swedish public data holders subject to obtaining ethics approval for the research and adhering to all relevant legislation, processes, and data protection protocols.

Funding information

ALF agreement (ALFGBG-938453, ALFGBG-971130, ALFGBG-978954, ALFGBG-966066), Swedish Research Council (2020-02828, 2019-01112), and Swedish Heart-Lung Foundation (20210030, 20210581).

Ethics approval statement

Ethical approval for this study was obtained from the Swedish Ethical Review Authority (2020-01800 with subsequent amendments).

Authors contributions

OR, ME, BK, FN, HL, DSO, and GJ were involved in the conceptualisation and design of the study. FN and HL acquired the data. BK and HL performed statistical analysis. OR, ME, BK, FN, HL, DSO, and GJ were involved with interpretation of the data and initial drafting of the manuscript. All authors participated in the critical appraisal, further interpretation, revision, and approved the final manuscript.

Competing interests statement

GJ has served as consultant for Ascendis Pharma, Astra Zeneca, and Novo Nordisk, and has received lecture fees from Novo Nordisk and Pfizer. DSO has received unrestricted project grants from Pfizer and is an employee at AstraZeneca as of August 8, 2021. FN holds some AstraZeneca shares. BK, ME, HL, and OR have nothing to disclose.

For peer review only

Abstract

Objectives:

While glucocorticoid (GC) treatment initiated for COVID-19 reduces mortality, it is unclear whether GC treatment prior to COVID-19 affects mortality. Long-term GC use raises infection and thromboembolic risks. We investigated if oral GC use prior to COVID-19 increase overall mortality.

Design: Population-based observational cohort study

Settings: Population-based register data in Sweden

Participants: All patients infected with COVID-19 in Sweden from January 2020 to November 2021 (n=1,200,153).

Outcome measures: Any prior oral GC use was defined as ≥1 GC prescription during 12 months before index. High exposure was defined as ≥2 GC prescriptions with a cumulative prednisolone dose ≥750 mg or equivalent during 6 months before index. GC users were compared with COVID-19 patients who had not received GCs within 12 months before index. We used Cox proportional hazard models and 1:2 propensity score matching to estimate HRs and 95% CIs, controlling for the same confounders in all analyses.

Results:

3378 deaths occurred in subjects with any prior GC exposure (n=48,806; 6·9%) and 14,850 among non-exposed (n=1,151,347; 1·3%). Both high (1·98, 1·87–2·09) and any exposure (1·58, 1·52–1·65) to GCs were associated with overall death. Deaths from pulmonary embolism, sepsis, and COVID-19 were associated with high GC

For peer review only - http://bmjopen.bmj.com/site/about/guidelines.xhtml

exposure and, similarly but weaker, with any exposure. High exposure to GCs was associated with increased deaths caused by stroke and myocardial infarction.

Conclusion:

Patients on oral GC treatment prior to COVID-19 have increased mortality, particularly from pulmonary embolism, sepsis, and COVID-19.

Key words: glucocorticoids, mortality, adrenal insufficiency, COVID-19

Strengths and limitations of this study

- Utilizes a population-based design
- First study to investigate if oral glucocorticoid exposure prior to COVID-19 affects mortality
- Employs adjusted Cox models and propensity score matching for confounder control. A large number of covariates, and a sizable comparison cohort.
- Observational nature may still introduce residual bias and unmeasured confounding.

For peer review only

INTRODUCTION

Oral glucocorticoids (GCs) are commonly prescribed for a variety of conditions, including inflammatory disorders as well as various allergic, respiratory, skin, and autoimmune conditions (1). In Sweden, 0.5% of the population receive long-term (>2 years) oral GC treatment (1), a treatment that is known to be associated with excess mortality mainly due to myocardial infarction, sepsis, and pulmonary embolism (2). Hypercortisolism is associated with a hypercoagulable state due to increased levels of procoagulant factors and impaired fibrinolysis (3). Patients receiving long-term GC treatment frequently also develop GC-induced adrenal insufficiency that renders them at increased risk of adrenal crisis, and subsequent death, during a severe infection (4).

Thromboembolic events, including pulmonary embolism and stroke, are common complications in patients with COVID-19 (5). Despite anticoagulation treatment, a notable proportion (16·7%) of patients with COVID-19 develop pulmonary embolism (5). COVID-19 patients may also develop arterial thrombosis, which can lead to serious outcomes such as stroke or myocardial infarction (6).

The use of oral GCs in COVID-19 patients has been a topic of research and debate. Current literature on GC therapy initiated for COVID-19 and effect on thromboembolic events warns that GC administration during COVID-19 may increase the risk of thromboembolic events, particularly in critically ill patients or those with pre-existing thrombotic risk factors (7). In May 2020, WHO published clinical guidelines on the management of COVID-19 and advised against the use of

For peer review only - http://bmjopen.bmj.com/site/about/guidelines.xhtml

GCs unless indicated for another reason (eg, exacerbation of asthma or chronic obstructive pulmonary disease) (8). At that time, high-dose GC therapy initiated for COVID-19 had been associated with delayed virus clearing, prolonged hospitalisation, and increased use of mechanical ventilation in patients with COVID-19 (9). In July 2020, the RECOVERY trial showed that dexamethasone treatment initiated in COVID-19 patients resulted in lower 28-day mortality among those requiring respiratory support (mechanical ventilation or oxygen alone) (10). Consequently, in September 2020, WHO revised the guidelines and recommended initiation of systemic GC treatment for patients with severe and critical COVID-19 (11). Nevertheless, GC treatment for COVID-19 patients is still controversial. GC therapy initiated for COVID-19 was related to increased death compared with conventional treatment in many observational studies, while beneficial effects on mortality were seen in randomised clinical trials (12). There is also a concern regarding the dose of GC therapy initiated for COVID-19, as higher doses have been shown to increase mortality rates in hypoxic patients not requiring ventilatory support (13). In the aforementioned studies, GC treatment was initiated for the treatment of COVID-19.

There is less known about how already present long-term GC treatment prior to COVID-19 effects outcome and mortality. An international study of patients with rheumatic disease receiving long-term GC treatment showed that prednisolone ≥10 mg/day prior to COVID-19 was associated with an increased hospitalisation rate (14). Chronic systemic GC therapy has also been associated with increased mortality in immunosuppressed patients with COVID-19 (15). Whether patients

For peer review only

receiving GC treatment for other conditions are at increased risk of death after developing COVID-19 has not been systematically studied.

Our hypothesis was that patients on oral GC treatment before being diagnosed with COVID-19 have an increased mortality from several conditions, including pulmonary embolism, myocardial infarction, stroke, sepsis, and COVID-19 itself compared with those who do not have GC treatment before COVID-19. To test this hypothesis, we investigated the association between prior GC treatment and the risk of mortality from these conditions among COVID-19 patients using population-based analysis.

METHODS

Study design, data sources, and study population

This was a register-based cohort study centred on the regularly updated SCIFI-PEARL (Swedish COVID-19 Investigation for Future Insights – a Population Epidemiology Approach using Register Linkage) project database,(16) which currently includes the entire Swedish population from 2015.

The study population consisted of individuals with COVID-19 who met the following criteria before Nov 30, 2021: (a) a positive test for SARS-CoV-2 in the National Database of Notifiable Communicable Diseases (SmiNet); (b) identified in the Swedish National Patient Register (NPR) with the International Classification of Diseases, revision 10 (ICD-10) codes U07.1 or U07.2 from a specialist visit or hospitalisation; or (c) identified in the Cause-of-Death Register with COVID-19 as an underlying or contributing cause of death. For each individual, their earliest date of

For peer review only - http://bmjopen.bmj.com/site/about/guidelines.xhtml

meeting any of these COVID-19 diagnosis criteria was considered their index date (baseline) in the study.

Additional data and sources used from the SCIFI-PEARL database included: socio-demographic data and dates of emigration or death from Statistics Sweden (Register of Total Population and the Longitudinal Integrated Database for Health Insurance and Labor Market Studies [LISA]) (17); prescribed medication data from the National Prescribed Drug Register (NPDR) (18); comorbidities from the NPR;(19) and intensive care data from the Swedish Intensive Care Register (SIR). Comprehensive data for each patient were obtained by linking their information across the different registers using the national personal identification number, as previously described (16). Ethical approval for this study was obtained from the Swedish Ethical Review Authority (2020-01800 with subsequent amendments).

For peer review only

Exposures, outcomes, and follow-up

Data from the NPDR were used to identify oral GC users as well as GC dosage and formulation (capsules, drops, or tablets). Prior oral GC treatment was defined in two ways: (a) any prior exposure as ≥1 dispensed prescriptions of oral GCs within 12 months before the index date; and (b) high exposure defined >2 dispensed prescriptions with a cumulative dose of prednisolone (Anatomical Therapeutic Chemical [ATC] code H02AB06) ≥750 mg, or equivalent amount (Appendix p 2) of betamethasone (H02AB01), dexamethasone (H02AB02), methylprednisolone (H02AB04), prednisone (H02AB07), hydrocortisone (H02AB09), cortisone (H02AB10), deflazacort (H02AB13), or a combination of these within 6 months before the index date. All patients belonging to the "high exposure" group were thus

also included in the "any prior exposure" group. The non-exposed comparison group included COVID-19 patients who had not received any oral GC prescriptions during the year before their index date.

The primary outcomes were overall death (all-cause mortality) and cause-specific death according to ICD-10 classification due to pulmonary embolism (I26), sepsis (A40, A41, or R57.2), myocardial infarction (I21–I23), stroke (I60–I64, I630–I635, I638, or I639), and COVID-19 (U07.1 or U07.2). Outcomes of cause-specific death were identified using the Cause-of-Death Register with the diagnosis being either the underlying or contributing cause. Secondary outcomes were two measures of severe COVID-19, hospitalisation for COVID-19, and direct admission to an intensive care unit, each defined by U07.1 or U07.2 as primary or secondary diagnosis, respectively, in the NPR or SIR. The different outcomes were all analysed separately. For each outcome, all subjects were followed from the index date (COVID-19 infection date) until the earliest of the following: an event for the specific outcome, emigration, death, or end of the study period (Nov 30, 2021).

Baseline covariates

The following baseline information was extracted from relevant registers (for details see Appendix p 3): (1) demographic factors from Statistics Sweden including age, sex, education, and employment; (2) comorbidities from the NPR as primary or secondary diagnoses for specialist outpatient or inpatient care from Jan 1, 2015 until the index date; and (3) dispensed prescribed medications from the NPDR during the year before the index date. Based on *a priori* selection, the following covariates were used for confounding adjustment in all analyses: age, sex, education, employment,

For peer review only

and relevant previous medical history (diabetes, deep vein thrombosis, pulmonary embolism, hypertension, stroke, ischaemic heart disease, heart failure, cancer, chronic obstructive pulmonary disease, rheumatologic conditions, and oral anticoagulation therapy).

Statistical analysis

The exposed and non-exposed groups were characterised using descriptive statistics with continuous variables presented as mean and standard deviation (SD), and categorical variables as number (%). Differences between exposed and non-exposed groups were characterised using standardised mean differences (SMDs). Kaplan-Meier curves were used to examine the outcomes over time during the study period. Cox proportional hazards regression was used to obtain hazard ratios (HRs) with 95% confidence intervals (CIs). In the main analysis, confounding was addressed by adjusting for the selected potential confounders in standard Cox models. In a sensitivity analysis, confounding was addressed by 1:2 matching on a propensity score that included the same set of confounders in the main analysis. The propensity score was estimated by fitting a logistic regression model with the selected covariates as predictors and oral GC exposure as independent variables. A 1:2 matching on the propensity score was then performed and evaluated by assessment of SMD pre- and post-matching for all variables and by visually checking the propensity score overlap. Analyses were performed in SAS, and R statistical software version 4.04 and matching was implemented using the MatchIT package in R (20).

For peer review only

Role of the funding sources

The funders of the study had no role in study design, data collection, data analysis, data interpretation, or writing of this manuscript.

RESULTS

Demographic and clinical characteristics

A cohort of 1 200 153 individuals with COVID-19 was identified with 48 806 having any GC exposure the year before index, including 13 493 individuals with high GC exposure during the last 6 months (table 1). The exposed groups were older and included more women, whereas the non-exposed group had a more even sex distribution. Substantial differences between the exposed and non-exposed (SMDs >0·2) were found for age, sex, and multiple comorbidities including cardiovascular, respiratory, cancer, and rheumatic disease (table 1). There were 3378 deaths in patients with any GC exposure (6·9%), of which 2023 deaths occurred in patients with high exposure (15·0%) and 14 850 deaths in the non-exposed group (1·3%) (Appendix p 4).

Association between GC exposure and death

Unadjusted cumulative incidence curves indicated that crude overall and cause-specific mortality was higher among GC users compared with non-users (figures 1 and 2).

In the main Cox regression analyses, the adjusted HR for all-cause mortality was 1·98 (95% CI 1·87–2·09) in the high exposure group and 1·58 (1·52–1·65) in

For peer review only - http://bmjopen.bmj.com/site/about/guidelines.xhtml

patients with any exposure compared with the non-exposed group (figure 3). High exposure to oral GCs was also associated with significantly increased risks of death from pulmonary embolism, sepsis, stroke, and COVID-19. Any prior exposure was similarly associated with death due to pulmonary embolism, sepsis, and COVID-19, but not significantly with fatal stroke (figure 3). The association for high exposure with death from myocardial infarction was similar in strength to that with fatal stroke, but marginally not significant (figure 3).

Association between GC exposure and severe COVID-19

Compared with the non-exposed group, the high exposure group demonstrated an elevated risk of hospitalisation for COVID-19 (adjusted HR 1·51, 95% CI 1·46–1·57) as well as intensive care unit admission (1·40, 1·26–1·55) (figure 3). There were also consistent but relatively weaker effects observed in patients with any prior exposure.

Sensitivity analysis with propensity score matching

Propensity score matching resulted in 91 878 non-exposed individuals matched to 47 737 individuals with any exposure and 23 898 individuals matched to 12 943 individuals with high exposure (Appendix pp 5–6). The numbers of outcome events in the matched cohorts are indicated in Appendix p 7. In the matched cohorts, the covariates of the exposed and matched non-exposed groups were well balanced (Appendix p 8) with good overlap of the propensity scores (Appendix p 10) and low absolute SMDs between the matched cohorts (Appendix pp 9 and 11).

The results obtained from the propensity score matched analysis were, overall, in good agreement with the adjusted Cox model (figure 3). Propensity score matching

For peer review only - http://bmjopen.bmj.com/site/about/guidelines.xhtml

indicated somewhat stronger effects of GC exposure on pulmonary embolism and myocardial infarction death, and weaker effects on sepsis and stroke death, severe COVID-19, and overall mortality in both high and any exposure categories (figure 3). In this analysis, high oral GC exposure was not significantly associated with death from stroke but, conversely, it was significantly and more strongly associated with myocardial infarction deaths.

For peer review only

DISCUSSION

This nationwide population-based cohort study of approximately 1·2 million individuals who had contracted COVID-19 infection showed increased mortality following the infection for 48 806 oral GC users compared with non-users, especially from sepsis, stroke, and pulmonary embolism. Individuals receiving oral GC treatment prior to the infection also had an increased risk of COVID-19-related outcomes including hospitalisation, intensive care unit admission, and death.

Comparison to other studies

Our study showed an increased risk of death from sepsis after contracting COVID-19 and the risk was greater in the high GC exposure group. Treatment with GCs is known to cause immunosuppression and GC users have increased susceptibility to infections (21). Similarly, studies on endogenous hypercortisolism and GC users show a high mortality risk due to infection in other settings (2 22). For example, one-third of deaths in patients with Cushing disease is due to infections (22) and GC users have generally increased mortality from sepsis and pneumonia (2). There is also evidence that higher doses of GCs are associated with an increased risk of

pneumonia and mortality in COVID-19 patients hospitalised with clinical hypoxia compared with standard low-dose GC treatment (13). Our results are also in line with observational data on patients with rheumatic diseases, showing that prednisolone dose ≥10mg/day prior to COVID-19 was associated with increased hospitalisation for COVID-19 (14).

We also found increased mortality from pulmonary embolism in GC users after contracting COVID-19. A generally increased incidence of thromboembolism in patients with endogenous hypercortisolism is known (23). A recent study showed that patients with Cushing's syndrome have a generally increased incidence of stroke and thromboembolism (23). Studies on GC users have reported similar findings with an approximately two-fold increased risk of both pulmonary embolism and deep vein thrombosis (24). The risk of comparable events after COVID-19 in the current study is similar in magnitude.

GC treatment was not significantly associated with death from stroke in our study, but it was significantly and more strongly associated with myocardial infarction deaths. The association was stronger in the high exposure group compared with the any prior exposure group. Our findings are in line with a population-based, nested, case-control study from the UK on oral GC and risk of cardiovascular and cerebrovascular disease based on data from 1988 to 1998 (25). The study showed increased risk of ischaemic heart disease (odds ratio [OR] 1·20, 95% CI 1·11–1·29) in current GC users, but not for increased risk of stroke/transient ischaemic attack (OR 0·95, 95% CI 0·89–1·01) (25).

For peer review only

We used both adjusted Cox models and propensity score matching to control for potential confounders in an effort to obtain unbiased estimates of the impact of oral GC use on severe outcomes. To clarify and understand imbalances in characteristics between the exposure groups before and after propensity score matching, we evaluated SMDs and achieved good balance after matching (26). It may be noted that the estimand for the adjusted Cox model is the conditional average treatment effect for the treated (ATE), ie, the effect if everyone were to be on treatment with oral GCs, conditional on their characteristics, while the estimand for the matched analysis is the average treatment effect for the treated (ATT), ie, the effect among those actually receiving oral GC treatment (27). ATE can be used to decide whether to mandate treatment for all patients, whereas ATT can be used to decide whether to withhold it from patients who would otherwise receive it. Nevertheless, the two methods produced comparable results that are clinically relevant and complementary.

Strengths and limitations of this study

This study has several strengths including a population-level sample, a large number of covariates, and a large comparison cohort which minimizes the risk of selection bias. However, since our study is observational, the analyses may still be subject to residual bias and unmeasured confounding, and thus the results require cautious interpretation. All GC users have an underlying disease that may increase mortality risk, potentially confounding the results. We mitigated this concern by adjusting for many diseases associated with GC treatment or increased mortality (eg, cancer, chronic obstructive pulmonary disease, rheumatologic conditions) using two analytical approaches, thus reducing the potential for major residual confounding. In

For peer review only

our study, we relied on a prescription register that only provided information about dispensed prescriptions, and thus compliance and actual use must be assumed (as is generally the case).

Unanswered questions and future research

The causal relationship between GC use and mortality remains a topic yet to be fully elucidated, primarily due to the fact that all long-term GC users have an underlying disease that may heighten mortality risk. Our study provides a comprehensive overview of excess mortality in this population. However, it's crucial to highlight that studying each underlying medical condition necessitates specific analytical considerations. To enhance our understanding of the factors contributing to excess mortality, future research should prioritize dissecting the precise impact of prolonged GC use on these underlying conditions.

Fundamentally, GC treatment suppresses the hypothalamic-pituitary-adrenal axis and can cause GC-induced adrenal insufficiency (4 28). Undiagnosed and untreated GC-induced adrenal insufficiency can lead to a life-threatening adrenal crisis. To prevent adrenal crisis, GC users should know how to increase their GC dose during sick days and during intercurrent illness (29). Awareness of GC-induced adrenal insufficiency is increasing. In 2020, guidance on the prevention of adrenal crisis during the COVID-19 pandemic was published and recommended that GC users increase their daily GC dose during COVID-19 infection and to take GC every 6–12 hours (depending on the type of GC used) (29). GC users who need to be admitted to hospital because of COVID-19 should receive GC intravenously (29).

For peer review only

CONCLUSION

Patients receiving GC treatment prior to COVID-19 infection face excess mortality in the aftermath of their acute COVID-19 infection, particularly from sepsis, pulmonary embolism, and COVID-19. These patients also have a high risk of intensive care unit admission and death due to COVID-19, and close monitoring and clinical attention may thus prevent their death.

Acknowledgements: We thank Peter Todd of Tajut Ltd. (Kaiapoi, New Zealand) for third-party editorial support, for which he received financial compensation from ALF funding.

For peer review only

References

1. Einarsdottir MJ, Ekman P, Trimpou P, et al. High prescription rate of oral glucocorticoids in children and adults: A retrospective cohort study from Western Sweden. *Clin Endocrinol (Oxf)* 2020;92(1):21-28.
2. Einarsdottir MJ, Ekman P, Molin M, et al. High Mortality Rate in Oral Glucocorticoid Users: A Population-Based Matched Cohort Study. *Front Endocrinol (Lausanne)* 2022;13:918356.
3. Manetti L, Bogazzi F, Giovannetti C, et al. Changes in coagulation indexes and occurrence of venous thromboembolism in patients with Cushing's syndrome: results from a prospective study before and after surgery. *Eur J Endocrinol* 2010;163(5):783-91.
4. Broersen LH, Pereira AM, Jorgensen JO, et al. Adrenal Insufficiency in Corticosteroids Use: Systematic Review and Meta-Analysis. *J Clin Endocrinol Metab* 2015;100(6):2171-80.
5. Helms J, Tacquard C, Severac F, et al. High risk of thrombosis in patients with severe SARS-CoV-2 infection: a multicenter prospective cohort study. *Intensive Care Med* 2020:1-10.
6. Katsoularis I, Fonseca-Rodríguez O, Farrington P, et al. Risk of acute myocardial infarction and ischaemic stroke following COVID-19 in Sweden: a self-controlled case series and matched cohort study. *Lancet* 2021;398(10300):599-607.
7. Sarfraz A, Sarfraz Z, Razzack AA, et al. Venous Thromboembolism, Corticosteroids and COVID-19: A Systematic Review and Meta-Analysis. *Clin Appl Thromb Hemost* 2021;27:1076029621993573.
8. World Health Organization. Clinical management of COVID-19: interim guidance, 27 May 2020 2020 [Available from: https://apps.who.int/iris/handle/10665/332196 accessed June 14 2023.
9. Li H, Chen C, Hu F, et al. Impact of corticosteroid therapy on outcomes of persons with SARS-CoV-2, SARS-CoV, or MERS-CoV infection: a systematic review and meta-analysis. *Leukemia* 2020;34(6):1503-11.
10. RECOVERY Collaborative Group; Horby P, Lim WS, Emberson JR, et al. Dexamethasone in hospitalized patients with Covid-19. *N Engl J Med* 2021; 25;384: 693–704
11. World Health Organization. Corticosteroids for COVID-19, Living guidance 2 September 2020 [Available from: https://www.who.int/publications/i/item/WHO-2019-nCoV-Corticosteroids-2020.1 accessed 8 October 2020.
12. Patel C, Parmar K, Patel D, et al. Effect of corticosteroid therapy on mortality in COVID-19 patients-A systematic review and meta-analysis. *Rev Med Virol* 2022;32(5):e2386.
13. Higher dose corticosteroids in patients admitted to hospital with COVID-19 who are hypoxic but not requiring ventilatory support (RECOVERY): a randomised, controlled, open-label, platform trial. *Lancet* 2023;401(10387):1499-507.
14. Gianfrancesco M, Hyrich KL, Al-Adely S, et al. Characteristics associated with hospitalisation for COVID-19 in people with rheumatic disease: data from the COVID-19 Global Rheumatology Alliance physician-reported registry. *Ann Rheum Dis* 2020;79(7):859-66.
15. Calderón-Parra J, Cuervas-Mons V, Moreno-Torres V, et al. Influence of chronic use of corticosteroids and calcineurin inhibitors on COVID-19 clinical outcomes: analysis of a nationwide registry. *Int J Infect Dis* 2022;116:51-58.
16. Nyberg F, Franzén S, Lindh M, et al. Swedish Covid-19 Investigation for Future Insights - A Population Epidemiology Approach Using Register Linkage (SCIFI-PEARL). *Clin Epidemiol* 2021;13:649-59.

For peer review only

17. Ludvigsson JF, Svedberg P, Olén O, et al. The longitudinal integrated database for health insurance and labour market studies (LISA) and its use in medical research. *Eur J Epidemiol* 2019;34(4):423-37.
18. The Swedish Prescribed Drug Register [Internet]. Socialstyrelsen. [cited 2021 Mar 29]. Available from: https://www.socialstyrelsen.se/en/statistics-and-data/registers/register-information/the-swedish-prescribed-drug-register/ [
19. The National Patient Register [Internet]. Socialstyrelsen. [cited 2021 Mar 29]. Available from: https://www.socialstyrelsen.se/en/statistics-and-data/registers/register-information/the-national-patient-register/ [
20. Stuart EA, King G, Imai K, et al. MatchIt: nonparametric preprocessing for parametric causal inference. *Journal of statistical software* 2011
21. Stuck AE, Minder CE, Frey FJ. Risk of infectious complications in patients taking glucocorticosteroids. *Rev Infect Dis* 1989;11(6):954-63.
22. Ragnarsson O, Olsson DS, Papakokkinou E, et al. Overall and Disease-Specific Mortality in Patients With Cushing Disease: A Swedish Nationwide Study. *J Clin Endocrinol Metab* 2019;104(6):2375-84.
23. Papakokkinou E, Olsson DS, Chantzichristos D, et al. Excess Morbidity Persists in Patients with Cushing's Disease during Long-term Remission - A Swedish Nationwide Study. *J Clin Endocrinol Metab* 2020
24. Johannesdottir SA, Horvath-Puho E, Dekkers OM, et al. Use of glucocorticoids and risk of venous thromboembolism: a nationwide population-based case-control study. *JAMA Intern Med* 2013;173(9):743-52.
25. Souverein PC, Berard A, Van Staa TP, et al. Use of oral glucocorticoids and risk of cardiovascular and cerebrovascular disease in a population based case-control study. *Heart* 2004;90(8):859-65.
26. Austin PC. Balance diagnostics for comparing the distribution of baseline covariates between treatment groups in propensity-score matched samples. *Statistics in medicine* 2009;28(25):3083-107.
27. Austin PC. The use of propensity score methods with survival or time-to-event outcomes: reporting measures of effect similar to those used in randomized experiments. *Statistics in medicine* 2014;33(7):1242-58.
28. Schlaghecke R, Kornely E, Santen RT, et al. The effect of long-term glucocorticoid therapy on pituitary-adrenal responses to exogenous corticotropin-releasing hormone. *N Engl J Med* 1992;326(4):226-30.
29. Arlt W, Baldeweg SE, Pearce SHS, et al. ENDOCRINOLOGY IN THE TIME OF COVID-19: Management of adrenal insufficiency. *Eur J Endocrinol* 2020;183(1):G25-g32.

For peer review only

Table 1: Baseline characteristics of individuals with COVID-19 infection in Sweden from January 2020 to November 2021 without and with glucocorticoid exposure prior to the infection

	Non-exposed (n=1 151 347)	Any prior GC exposure* (n=48 806)		High GC exposure† (n=13 497)	
			SMD *vs* non-exposed		SMD *vs* non-exposed
Age (years)	37·9 (19·2)	51·4 (21·2)	0·666	62·4 (19·7)	1·257
Age category (years)			0·672		1·276
0–9	56 461 (4·9%)	930 (1·9%)		91 (0·7%)	
10–19	171 590 (14·9%)	3056 (6·3%)		284 (2·1%)	
20–29	200 477 (17·4%)	4491 (9·2%)		567 (4·2%)	
30–39	200 414 (17·4%)	5882 (12·1%)		925 (6·9%)	
40–49	205 427 (17·8%)	8045 (16·5%)		1514 (11·2%)	
50–59	167 108 (14·5%)	8993 (18·4%)		2249 (16·7%)	
60–69	79 704 (6·9%)	6396 (13·1%)		188 (16·2%)	
70–79	37 797 (3·3%)	5725 (11·7%)		2680 (19·9%)	
80–89	23 055 (2·0%)	4123 (8·4%)		2302 (17·1%)	
≥90	9314 (0·8%)	1165 (2·4%)		697 (5·2%)	
Sex			0·149		0·147
Male	566 579 (49·2%)	20 407 (41·8%)		5659 (41·9%)	
Female	584 768 (50·8%)	28 399 (58·2%)		7838 (58·1%)	
Birth country			0·105		0·199
Sweden	883 118 (76·7%)	38 515 (78·9%)		10 923 (80·9%)	
Nordic	18 577 (1·6%)	1285 (2·6%)		484 (3·6%)	
Europe	83 455 (7·2%)	3142 (6·4%)		779 (5·8%)	
Other	166 197 (14·4%)	5864 (12·0%)		1311 (9·7%)	
Employment status			0·453		0·888

For peer review only

Employed	838 341 (72·8%)	31 232 (64·0%)		6432 (47·7%)	
Unemployed	172 951 (15·0%)	15 328 (31·4%)		6857 (50·8%)	
Unknown	140 055 (12·2%)	2246 (4·6%)		208 (1·5%)	
Education level			0·300		0·501
Primary	173 194 (15·0%)	9817 (20·1%)		3672 (27·2%)	
Secondary	426 657 (37·1%)	19 950 (40·9%)		5656 (41·9%)	
Higher	376 185 (32·7%)	15 855 (32·5%)		3714 (27·5%)	
Unknown	175 311 (15·2%)	3184 (6·5%)		455 (3·4%)	
Comorbidities					
Pulmonary embolism	7977 (0·7%)	1676 (3·4%)	0·194	853 (6·3%)	0·310
Stroke	12 140 (1·1%)	1679 (3·4%)	0·161	852 (6·3%)	0·282
Deep vein thrombosis	1085 (0·1%)	301 (0·6%)	0·088	97 (0·7%)	0·098
Hypertension	86 847 (7·5%)	13 486 (27·6%)	0·547	6255 (46·3%)	0·972
Ischaemic heart disease	26 541 (2·3%)	4370 (9·0%)	0·291	2147 (15·9%)	0·487
Overall CV disease	149 594 (13·0%)	19 033 (39·0%)	0·621	8293 (61·4%)	1·158
Heart failure	18 043 (1·6%)	4456 (9·1%)	0·341	2347 (17·4%)	0·561
Asthma	37 185 (3·2%)	7841 (16·1%)	0·445	2026 (15·0%)	0·418
COPD	8731 (0·8%)	3856 (7·9%)	0·356	1700 (12·6%)	0·488
Emphysema	793 (0·1%)	392 (0·8%)	0·112	199 (1·5%)	0·161
Diabetes mellitus	42 598 (3·7%)	5478 (11·2%)	0·289	2638 (19·5%)	0·510
Rheumatic disease	8727 (0·8%)	6568 (13·5%)	0·510	4109 (30·4%)	0·897
Cancer	44 717 (3·9%)	7666 (15·7%)	0·406	3511 (26·0%)	0·653
Prescribed medications					
Inhaled corticosteroids	120 572 (10·5%)	17 815 (36·5%)	0·645	4258 (31·5%)	0·536
Oral anticoagulants	57 018 (5·0%)	8693 (17·8%)	0·413	3992 (29·6%)	0·689
Statins	101 469 (8·8%)	11 096 (22·7%)	0·389	4556 (33·8%)	0·640
ACEIs	67 321 (5·8%)	6969 (14·3%)	0·283	2860 (21·2%)	0·460
Opioids	112 707 (9·8%)	12 505 (25·6%)	0·424	4523 (33·5%)	0·601

Data are n (%) or mean (SD). *One or more prescriptions of oral GCs within 12 months before the COVID-19 infection date. †Two or more prescriptions and prednisolone ≥750 mg or equivalent amount of betamethasone, dexamethasone, methylprednisolone, prednisone, hydrocortisone, cortisone, or deflazacort within 6 months before COVID-19 infection date. Note this group is a subset

For peer review only

of any prior exposure. ACEI, angiotensin-converting enzyme inhibitor; COPD, chronic obstructive pulmonary disease; CV, cardiovascular; GC, glucocorticoid; SMD, standardised mean difference.

For peer review only

Figure legends

Figure 1: **Unadjusted cumulative incidence of all-cause (a) and cause-specific (b–f) mortality among COVID-19 patients in Sweden with any prior exposure* to oral glucocorticoids compared to non-exposed, from COVID-19 infection date to Nov 30, 2021**

GC, glucocorticoids. *Any prior GC exposure equals ≥1 prescription of oral GC within 12 months before the COVID-19 infection date.

Figure 2: **Unadjusted cumulative incidence of all-cause (a) and cause-specific (b–f) mortality among COVID-19 patients with any prior exposure* to oral glucocorticoids compared to non-exposed, from COVID-19 infection date to Nov 30, 2021**

GC, glucocorticoids. *High GC exposure equals ≥2 prescriptions with a total of ≥750 mg of prednisolone or equivalent within 6 months before COVID-19 infection date. This group is a subset of any prior exposure see figure 1).

Figure 3: **Adjusted Cox regression analysis and propensity score matched analysis comparing the risk of all-cause and cause-specific mortality between COVID-19 patients with prior exposure to oral glucocorticoids and non-exposed patients**

CI, confidence interval; HR, hazard ratio; ICU, intensive care unit; PS, propensity score. Any prior GC exposure equals ≥1 prescription of oral GC within 12 months before the COVID-19 infection date. High GC exposure equals ≥2 prescriptions with a total of ≥750 mg of prednisolone or equivalent within 6 months before COVID-19

For peer review only

infection date. The latter group is a subset of any prior exposure. *Adjusted for covariates; †Propensity score matched on covariates.

For peer review only

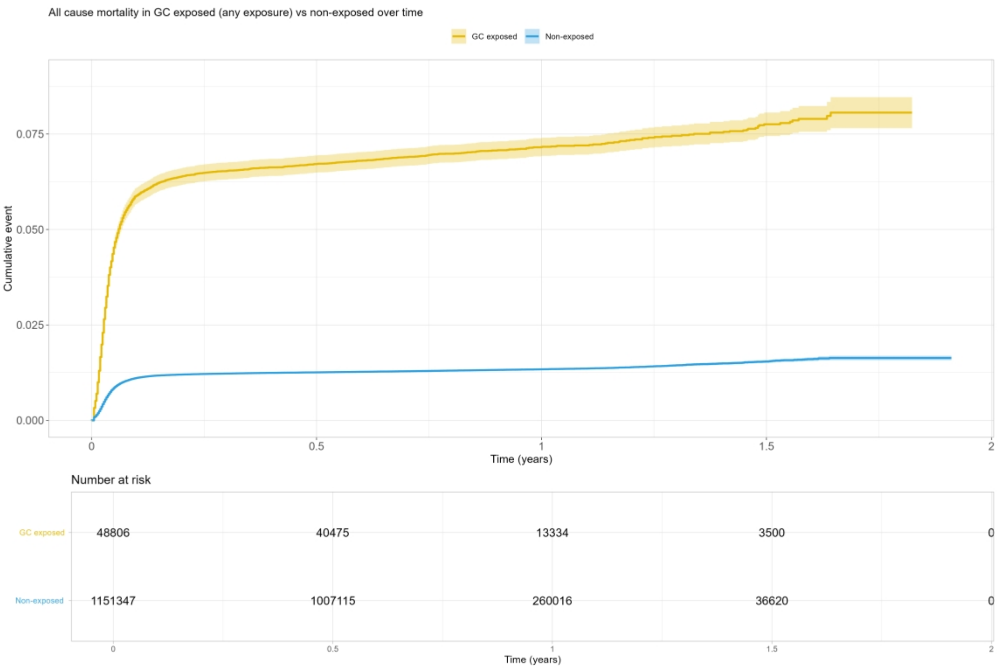

Figure 1a: Unadjusted cumulative incidence of all-cause (a) and cause-specific (b–f) mortality among COVID-19 patients in Sweden with any prior exposure* to oral glucocorticoids compared to non-exposed, from COVID-19 infection date to Nov 30, 2021
GC, glucocorticoids. *Any prior GC exposure equals ≥1 prescription of oral GC within 12 months before the COVID-19 infection date.

968x645mm (118 x 118 DPI)

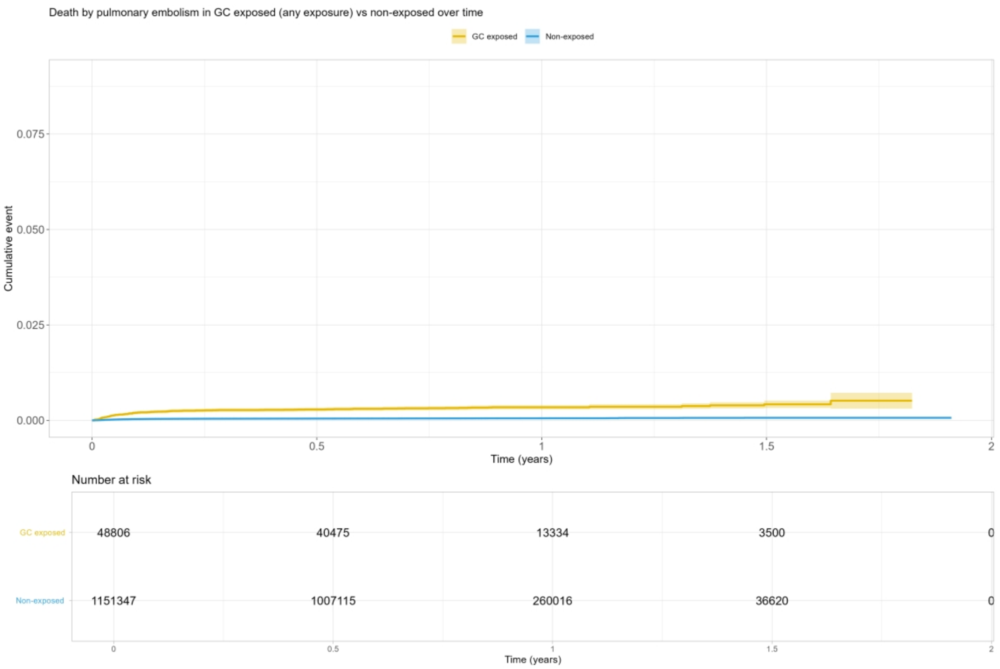

Figure 1b: Unadjusted cumulative incidence of all-cause (a) and cause-specific (b–f) mortality among COVID-19 patients in Sweden with any prior exposure* to oral glucocorticoids compared to non-exposed, from COVID-19 infection date to Nov 30, 2021
GC, glucocorticoids. *Any prior GC exposure equals ≥1 prescription of oral GC within 12 months before the COVID-19 infection date.

968x645mm (118 x 118 DPI)

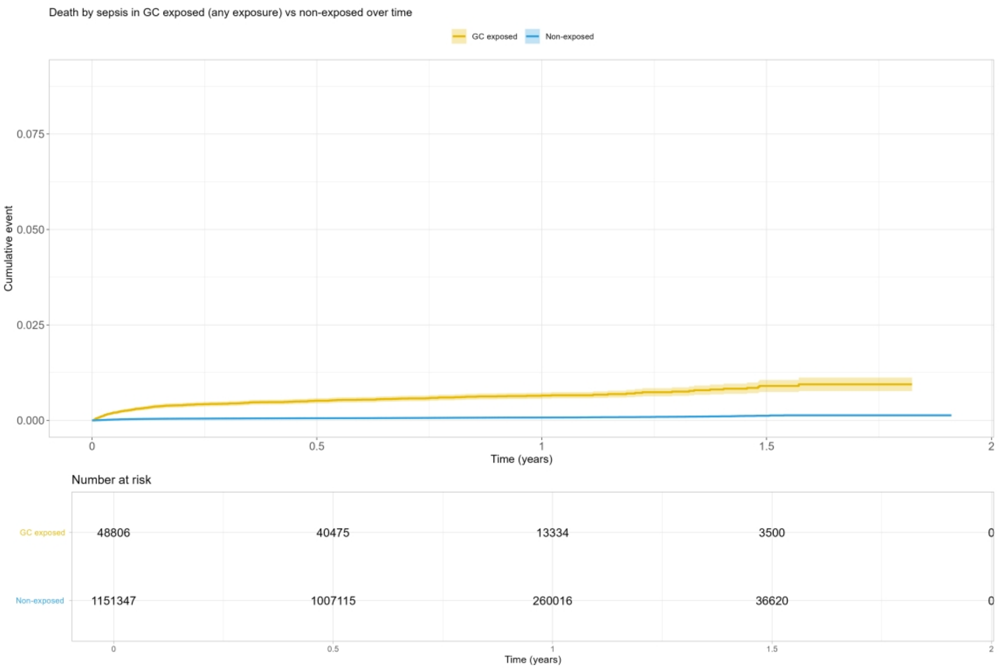

Figure 1c: Unadjusted cumulative incidence of all-cause (a) and cause-specific (b–f) mortality among COVID-19 patients in Sweden with any prior exposure* to oral glucocorticoids compared to non-exposed, from COVID-19 infection date to Nov 30, 2021
GC, glucocorticoids. *Any prior GC exposure equals ≥1 prescription of oral GC within 12 months before the COVID-19 infection date.

968x645mm (118 x 118 DPI)

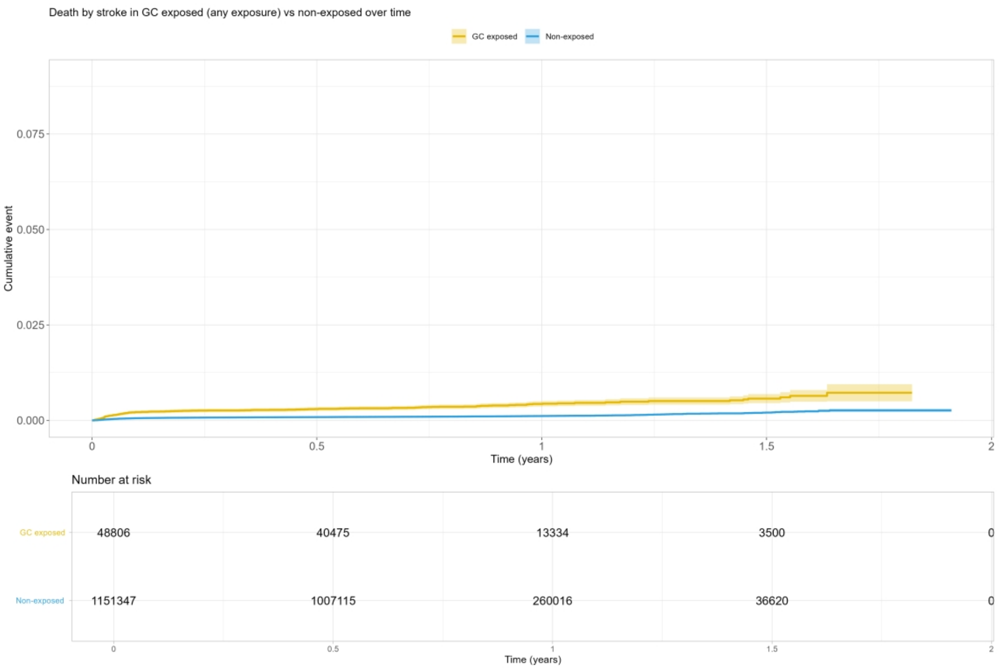

Figure 1d: Unadjusted cumulative incidence of all-cause (a) and cause-specific (b–f) mortality among COVID-19 patients in Sweden with any prior exposure* to oral glucocorticoids compared to non-exposed, from COVID-19 infection date to Nov 30, 2021
GC, glucocorticoids. *Any prior GC exposure equals ≥1 prescription of oral GC within 12 months before the COVID-19 infection date.

968x645mm (118 x 118 DPI)

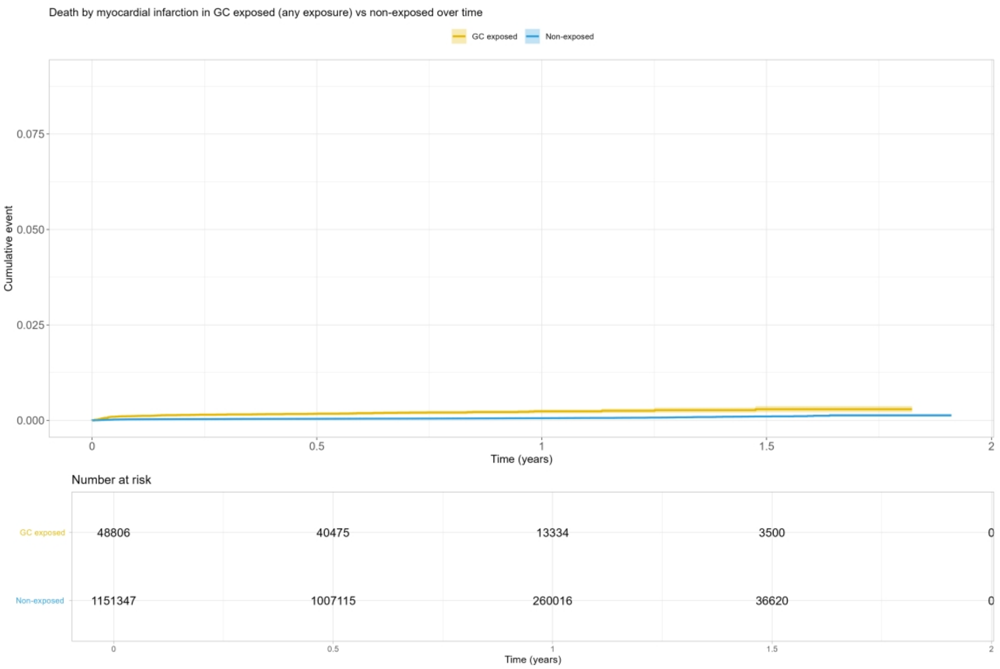

Figure 1e: Unadjusted cumulative incidence of all-cause (a) and cause-specific (b–f) mortality among COVID-19 patients in Sweden with any prior exposure* to oral glucocorticoids compared to non-exposed, from COVID-19 infection date to Nov 30, 2021
GC, glucocorticoids. *Any prior GC exposure equals ≥1 prescription of oral GC within 12 months before the COVID-19 infection date.

968x645mm (118 x 118 DPI)

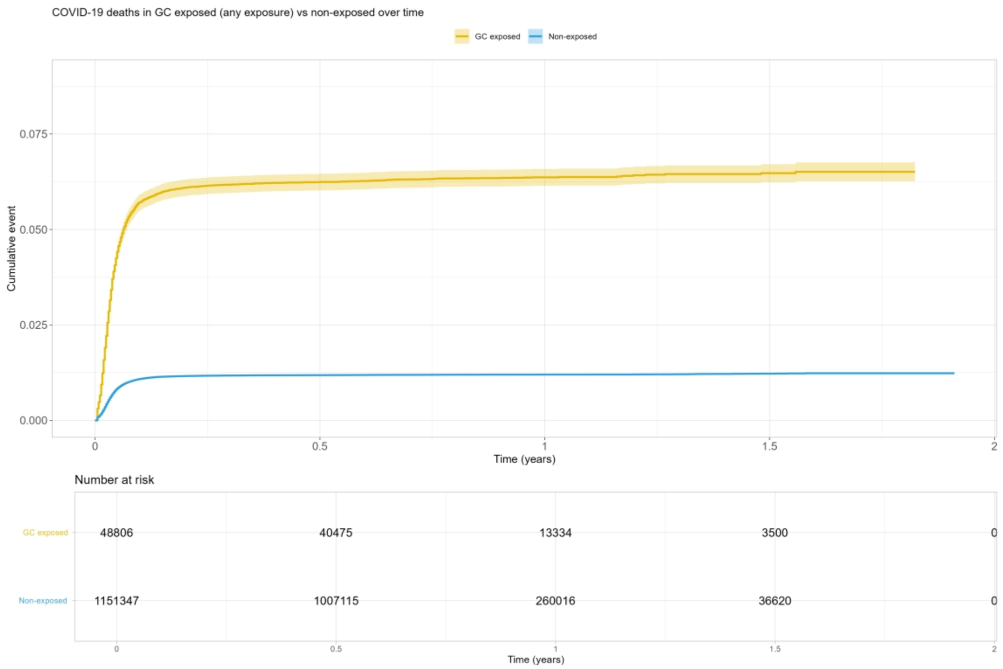

Figure 1f: Unadjusted cumulative incidence of all-cause (a) and cause-specific (b–f) mortality among COVID-19 patients in Sweden with any prior exposure* to oral glucocorticoids compared to non-exposed, from COVID-19 infection date to Nov 30, 2021
GC, glucocorticoids. *Any prior GC exposure equals ≥1 prescription of oral GC within 12 months before the COVID-19 infection date.

968x645mm (118 x 118 DPI)

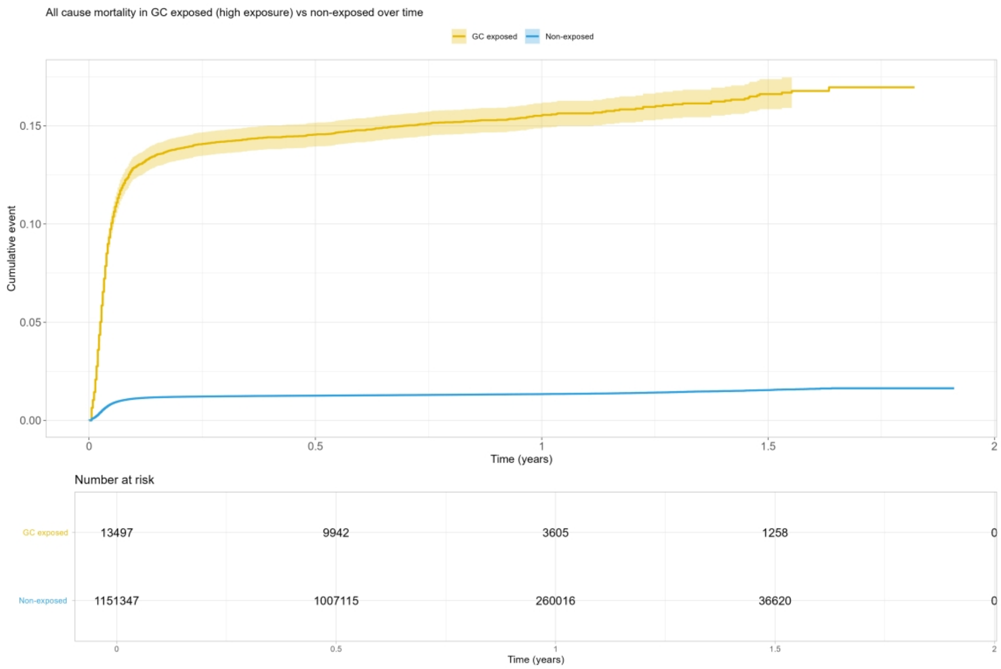

Figure 2a: Unadjusted cumulative incidence of all-cause (a) and cause-specific (b–f) mortality among COVID-19 patients with any prior exposure* to oral glucocorticoids compared to non-exposed, from COVID-19 infection date to Nov 30, 2021
GC, glucocorticoids. *High GC exposure equals ≥2 prescriptions with a total of ≥750 mg of prednisolone or equivalent within 6 months before COVID-19 infection date. This group is a subset of any prior exposure see figure 1).

968x645mm (118 x 118 DPI)

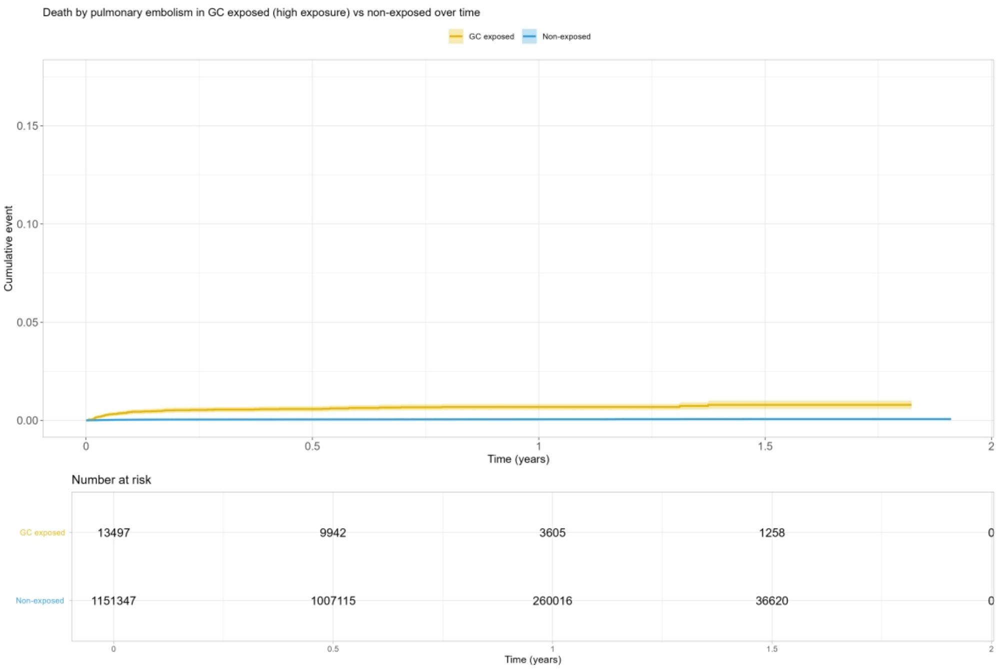

Figure 2b: Unadjusted cumulative incidence of all-cause (a) and cause-specific (b–f) mortality among COVID-19 patients with any prior exposure* to oral glucocorticoids compared to non-exposed, from COVID-19 infection date to Nov 30, 2021
GC, glucocorticoids. *High GC exposure equals ≥2 prescriptions with a total of ≥750 mg of prednisolone or equivalent within 6 months before COVID-19 infection date. This group is a subset of any prior exposure see figure 1).

968x645mm (118 x 118 DPI)

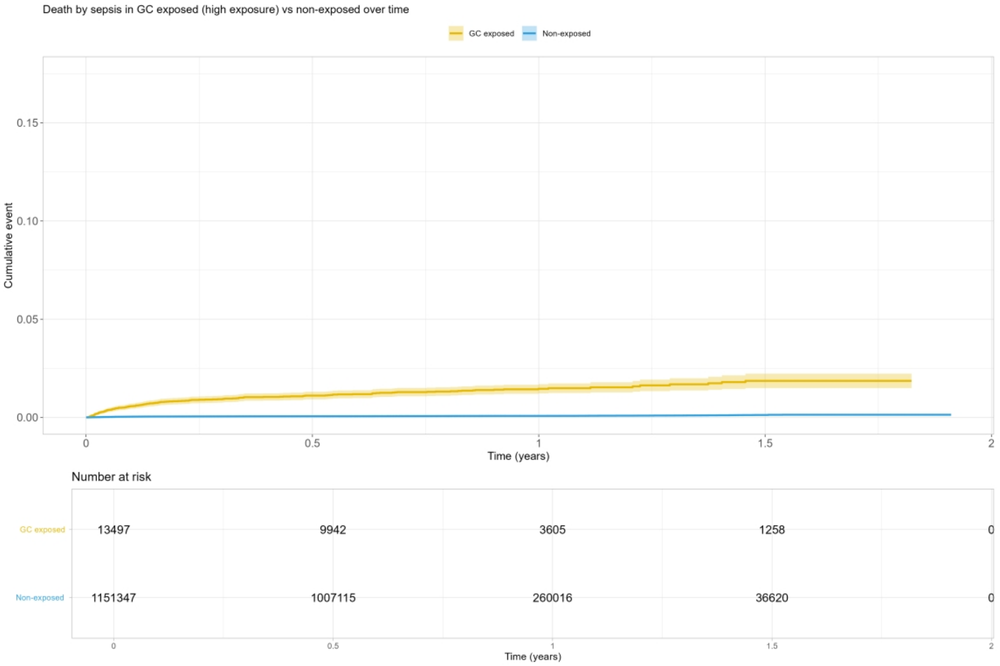

Figure 2c: Unadjusted cumulative incidence of all-cause (a) and cause-specific (b–f) mortality among COVID-19 patients with any prior exposure* to oral glucocorticoids compared to non-exposed, from COVID-19 infection date to Nov 30, 2021
GC, glucocorticoids. *High GC exposure equals ≥2 prescriptions with a total of ≥750 mg of prednisolone or equivalent within 6 months before COVID-19 infection date. This group is a subset of any prior exposure see figure 1).

968x645mm (118 x 118 DPI)

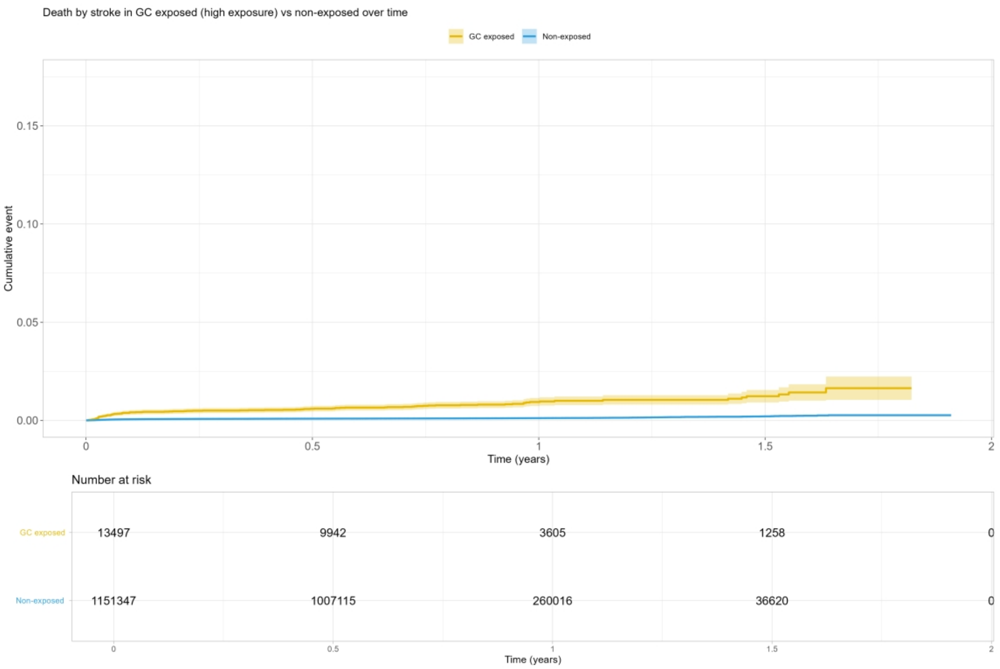

Figure 2d: Unadjusted cumulative incidence of all-cause (a) and cause-specific (b–f) mortality among COVID-19 patients with any prior exposure* to oral glucocorticoids compared to non-exposed, from COVID-19 infection date to Nov 30, 2021
GC, glucocorticoids. *High GC exposure equals ≥2 prescriptions with a total of ≥750 mg of prednisolone or equivalent within 6 months before COVID-19 infection date. This group is a subset of any prior exposure see figure 1).

968x645mm (118 x 118 DPI)

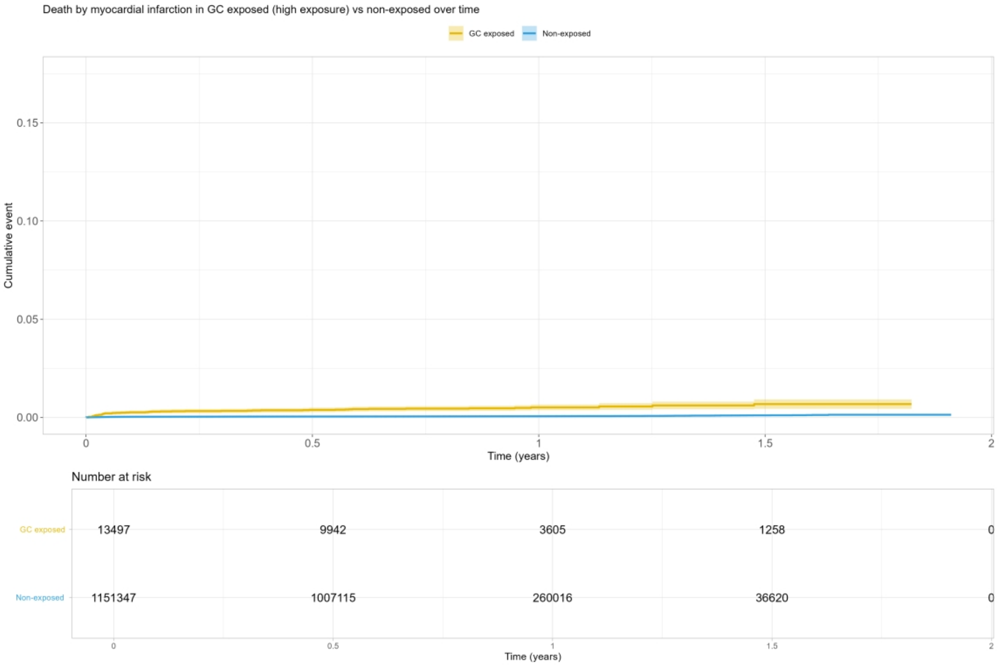

Figure 2e: Unadjusted cumulative incidence of all-cause (a) and cause-specific (b–f) mortality among COVID-19 patients with any prior exposure* to oral glucocorticoids compared to non-exposed, from COVID-19 infection date to Nov 30, 2021
GC, glucocorticoids. *High GC exposure equals ≥2 prescriptions with a total of ≥750 mg of prednisolone or equivalent within 6 months before COVID-19 infection date. This group is a subset of any prior exposure see figure 1).

968x645mm (118 x 118 DPI)

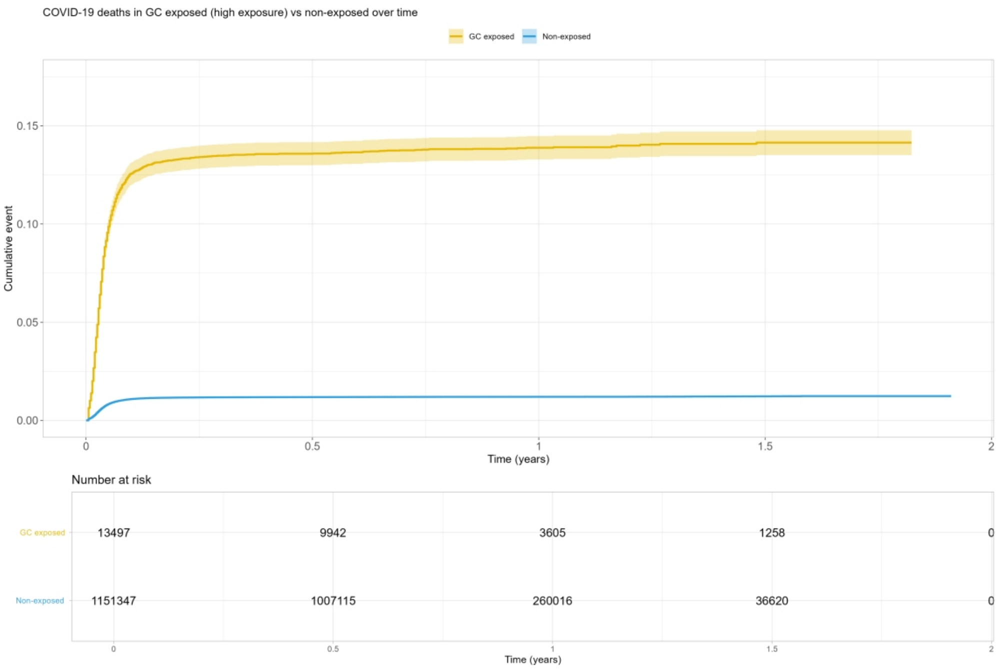

Figure 2f: Unadjusted cumulative incidence of all-cause (a) and cause-specific (b–f) mortality among COVID-19 patients with any prior exposure* to oral glucocorticoids compared to non-exposed, from COVID-19 infection date to Nov 30, 2021
GC, glucocorticoids. *High GC exposure equals ≥2 prescriptions with a total of ≥750 mg of prednisolone or equivalent within 6 months before COVID-19 infection date. This group is a subset of any prior exposure see figure 1).

968x645mm (118 x 118 DPI)

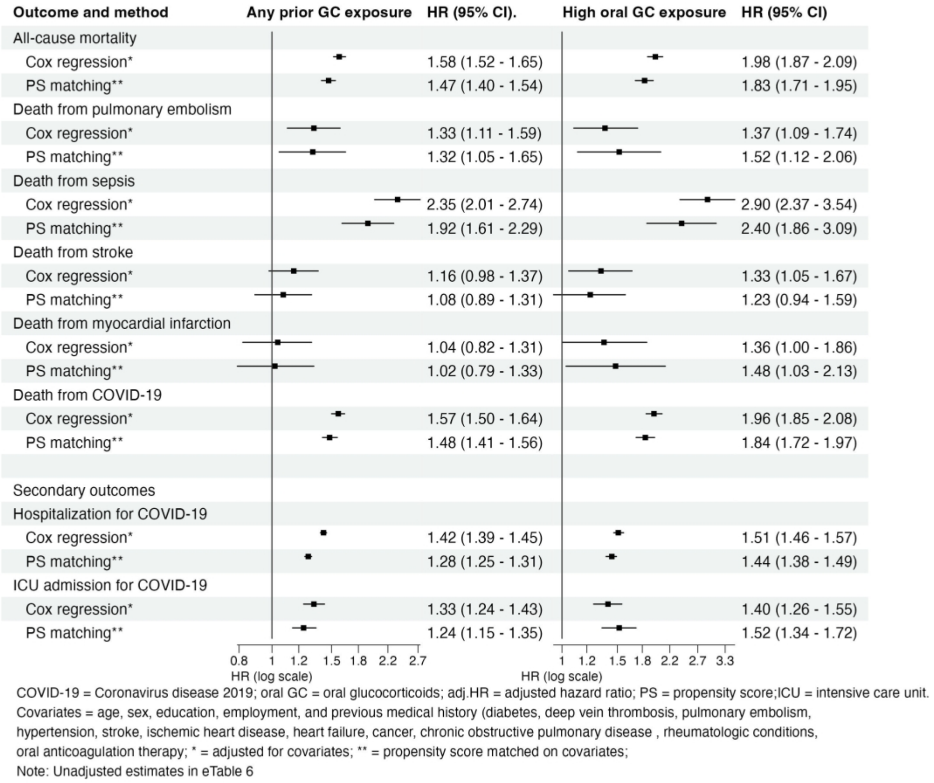

Figure 3: Adjusted Cox regression analysis and propensity score matched analysis comparing the risk of all-cause and cause-specific mortality between COVID-19 patients with prior exposure to oral glucocorticoids and non-exposed patients

CI, confidence interval; HR, hazard ratio; ICU, intensive care unit; PS, propensity score. Any prior GC exposure equals ≥1 prescription of oral GC within 12 months before the COVID-19 infection date. High GC exposure equals ≥2 prescriptions with a total of ≥750 mg of prednisolone or equivalent within 6 months before COVID-19 infection date. The latter group is a subset of any prior exposure. *Adjusted for covariates; †Propensity score matched on covariates.

971x932mm (72 x 72 DPI)

Supplementary appendix

Supplementary Table S1: **Dose equivalents used in the calculation of equivalence to prednisolone..................2**

Supplementary Table S2: **Covariates selected in the evaluation of the effect of glucocorticoid treatment on death in COVID-19 patients in the Swedish population-based cohort of COVID-19 patients from COVID-19 infection date to Nov 30, 2021...3**

Supplementary Table S3: **Outcome events in individuals with COVID-19 infection in the Swedish population during the period 2015 to COVID-19 infection date until Nov 30, 2021 stratified by level of exposure to oral glucocorticoids (non-exposed, high exposure in the 6 months before infection, or any exposure 12 months before infection and overall)...4**

Supplementary Table S4: **Baseline characteristics of individuals with COVID-19 infection in the Swedish population during the period 2015 to COVID-19 infection date (comorbidities) or 1 year before COVID-19 infection date (medications) until 30 Nov 2021, stratified by level of exposure to oral glucocorticoids (high exposure in the 6 months before infection or any exposure 12 months before infection) with standardised mean differences between exposed and 1:2 propensity score matched non-exposed..5**

Supplementary Table S5: **Outcome events in propensity score matched cohorts of individuals with COVID-19 infection in the Swedish population during the period 2015 to COVID-19 infection date until Nov 30, 2021 stratified by level of exposure to oral glucocorticoids (non-exposed, high exposure in the 6 months before infection, or any exposure 12 months before infection and overall)..7**

Supplementary Table S6: **Standardised mean differences between the 15 covariates of individuals with COVID-19 infections in the Swedish population during the period 2015 to COVID-19 infection date (comorbidities) or 1 year before COVID-19 infection date (medications) in those exposed to oral glucocorticoids compared with those non-exposed before and after propensity score matching.....................8**

Supplementary Table S7: **Unadjusted and adjusted hazard ratios from Cox regression analyses and propensity score matched analysis with 95% confidence intervals comparing the risk of various causes of death and all-cause mortality between COVID-19 patients who had any or high prior exposure to oral glucocorticoids and non-exposed individuals...9**

Supplementary Figure S1: **Propensity score balance before (a, c) and after (b, d) performing propensity score matching on all 15 covariates for individuals with COVID-19 infections in the Swedish population between 2015 and the COVID-19 infection date comparing those exposed to oral glucocorticoids (pink) with those who were non-exposed (blue) in those with any (a, b) or high glucocorticoid exposure (c, d)...10**

Supplementary Figure S2: **Standardised mean differences between the covariates of individuals with COVID-19 infections in the Swedish population during the period 2015 to COVID-19 infection date (comorbidities) or 1 year before COVID-19 infection date (medications) in the exposed to oral glucocorticoids compared to non-exposed before (red circles) and after (blue triangles) propensity score matching among those with (a) any or (b) high glucocorticoid exposure...11**

For peer review only

Supplementary Table S1: **Dose equivalents used in the calculation of equivalence to prednisolone**

ATC code	Recommended international non-proprietary drug name	Equivalent dose to 1 mg of prednisolone (mg)	Equivalent dose to 750 mg of prednisolone (mg)*
H02AB01	Betamethasone	8·333	6250·0
H02AB02	Dexamethasone	6·666	5000·0
H02AB04	Methylprednisolone	1·25	937·5
H02AB06	Prednisolone	1	750·0
H02AB07	Prednisone	1	750·0
H02AB09	Hydrocortisone	0·25	187·5
H02AB10	Cortisone	0·2	150·0
H02AB13	Deflazacort	0·666	500·0

ATC, Anatomical Therapeutic Chemical. *The definition of high oral glucocorticoid exposure was ≥2 prescriptions of prednisolone ≥750 mg or equivalent within 6 months before COVID-19 infection date. The corresponding equivalent amount to prednisolone 750 mg or the other oral glucocorticoids is shown on this column.

For peer review only

Supplementary Table S2: **Covariates selected in the evaluation of the effect of glucocorticoid treatment on death in COVID-19 patients in the Swedish population-based cohort of COVID-19 patients from COVID-19 infection date to Nov 30, 2021**

Covariate	Units and categories	Register source; description	Lookback window before COVID-19 infection date (index date)
Sociodemographic characteristics			
Age	As a continuous variable and in 10-year categories	RTB	At Jan 1, 2020
Sex	Male, female	RTB	At Jan 1, 2020
Education	Primary, secondary, higher, or unknown	LISA: primary education included pre-secondary education <9, 9, or >9 years. Secondary educated included 2-year high school education, secondary education <3 years or >3 years, and higher education included those with post-high school education. Unknown includes children with no education information or individuals with no relevant education (eg, immigrants).	At Jan 1, 2020
Employment status	Employed, unemployed, or unknown	LISA: created using the variable "employment" and stratified by age where: "employed" was either employed or with entrepreneurial income, and "unemployed" was the unemployed and without entrepreneurial income and includes pensioners. Unknown includes individuals with no information on employment.	At Jan 1, 2020
Comorbidity	**Units/categories**	**NPR: ICD-10 codes, any mention of condition as primary or secondary diagnosis for a hospitalisation or ≥1 visit at any time**	Jan 1, 2015 to index date
Pulmonary embolism	Yes/No	NPR: I26	Jan 1, 2015 to index date
Stroke	Yes/No	NPR: I61 I63 I649 I691 I693 I694 I698	Jan 1, 2015 to index date
Deep vein thrombosis	Yes/No	NPR: I80–I82	Jan 1, 2015 to index date
Hypertension	Yes/No	NPR: I10–I15	Jan 1, 2015 to index date
Ischaemic heart disease	Yes/No	NPR: I20 –I25	Jan 1, 2015 to index date
Overall cardiovascular disease	Yes/No	NPR: I00–I99	Jan 1, 2015 to index date
Heart failure	Yes/No	NPR: I50	Jan 1, 2015 to index date
Asthma	Yes/No	NPR: J45	Jan 1, 2015 to index date
COPD	Yes/No	NPR: J44	Jan 1, 2015 to index date
Emphysema	Yes/No	NPR: J43	Jan 1, 2015 to index date
Interstitial lung disease	Yes/No	NPR: J84	Jan 1, 2015 to index date
Diabetes	Yes/No	NPR: E10, E11, E13, E14	Jan 1, 2015 to index date
Rheumatic disease	Yes/No	NPR: M05, M06, M31, M35	Jan 1, 2015 to index date
Cancer	Yes/No	NPR: C00–C97	Jan 1, 2015 to index date
Prescribed medications	**Units/categories**	**NPDR: ATC codes. At least one filled prescription in lookback window**	
Inhaled corticosteroids	Yes/No	R03BA, R03AK, R03AL08, R03AL09	1 year before index
Oral anticoagulants	Yes/No	B01AA, B01AF	1 year before index
Statins	Yes/No	C10AA	1 year before index
ACEIs	Yes/No	C09A	1 year before index
Opioids	Yes/No	N02AB	1 year before index

ACEI, angiotensin-converting enzyme inhibitor; ATC, Anatomical Therapeutic Chemical; BMI, body mass index; COPD, chronic obstructive pulmonary disease; ICD-10, International Classification of Diseases, version 10; LISA, Longitudinal Integrated Database for Health Insurance and Labor Market Studies; NPDR, Swedish National Prescribed Drug Register; NPR, Swedish National Patient Register; RTB, Register of Total Population; SNAR, Swedish National Airways Register

For peer review only

Supplementary Table S3: **Outcome events in individuals with COVID-19 infection in the Swedish population during the period 2015 to COVID-19 infection date until Nov 30, 2021 stratified by level of exposure to oral glucocorticoids (non-exposed, high exposure in the 6 months before infection, or any exposure 12 months before infection and overall)**

	No=0/Yes=1	Non-exposed (n=1 151 347)	Any GC exposure* (n=48 806)	High GC exposure† (n=13 497)	Total study population (n=1 200 153)
All-cause mortality					
	0	1 136 497 (98·7%)	45 428 (93·1%)	11 474 (85·0%)	1 181 925 (98·5%)
	1	14 850 (1·3%)	3378 (6·9%)	2023 (15·0%)	18 228 (1·5%)
Death from pulmonary embolism					
	0	1 150 769 (99·9%)	48 654 (99·7%)	13 417 (99·4%)	1 199 423 (99·9%)
	1	578 (0·1%)	152 (0·3%)	80 (0·6%)	730 (0·1%)
Death from sepsis					
	0	1 150 576 (99·9%)	48 520 (99·4%)	13 335 (98·8%)	1 199 096 (99·9%)
	1	771 (0·1%)	286 (0·6%)	162 (1·2%)	1057 (0·1%)
Death from myocardial infarction					
	0	1 150 755 (99·9%)	48 707 (99·8%)	13 440 (99·6%)	1 199 462 (99·9%)
	1	592 (0·1%)	99 (0·2%)	57 (0·4%)	691 (0·1%)
Death from stroke					
	0	1 150 152 (99·9%)	48 625 (99·6%)	13 396 (99·3%)	1 198 777 (99·9%)
	1	1195 (0·1%)	181 (0·4%)	101 (0·7%)	1376 (0·1%)
Death from COVID-19					
	0	1 137 876 (98·8%)	45 781 (93·8%)	11 678 (86·5%)	1 183 657 (98·6%)
	1	13 471 (1·2%)	3025 (6·2%)	1819 (13·5%)	16 496 (1·4%)
Hospitalisation for COVID-19					
	0	1 088 788 (94·6%)	38 383 (78·6%)	8460 (62·7%)	1 127 171 (93·9%)
	1	62 559 (5·4%)	10 423 (21·4%)	5037 (37·3%)	72 982 (6·1%)
ICU admission for COVID-19					
	0	1 144 510 (99·4%)	47 747 (97·8%)	13 004 (96·3%)	1 192 257 (99·3%)
	1	6837 (0·6%)	1059 (2·2%)	493 (3·7%)	7896 (0·7%)

GC, glucocorticoid; ICU, intensive care unit. *One or more prescriptions of oral GCs within 12 months before the COVID-19 infection date. †Two or more prescriptions and prednisolone ≥750 mg or equivalent within 6 months before COVID-19 infection date. Note this group is a subset of any prior exposure.

For peer review only

Supplementary Table S4: **Baseline characteristics of individuals with COVID-19 infection in the Swedish population during the period 2015 to COVID-19 infection date (comorbidities) or 1 year before COVID-19 infection date (medications) until Nov 30, 2021, stratified by level of exposure to oral glucocorticoids (high exposure in the 6 months before infection or any exposure 12 months before infection), with standardized mean differences between exposed and 1:2 propensity score matched non-exposed**

	Any prior GC exposure* (n=47 737)	Non-exposed matched to any prior exposure (n=91 878)	SMD (any prior *vs* matched non-exposed)	High GC exposure† (n=12 943)	Non-exposed matched to the high GC exposed (n=23 898)	SMD (high *vs* matched non-exposed)
Age (years)	50·83 (21·06)	49·95 (20·83)	0·042	61·60 (19·69)	60·17 (19·63)	0·073
Age category (years)			0·047			0·081
0–9	930 (1·9)	1840 (2·0)		91 (0·7)	174 (0·7)	
10–19	3056 (6·4)	6089 (6·6)		284 (2·2)	556 (2·3)	
20–29	4491 (9·4)	8982 (9·8)		567 (4·4)	1128 (4·7)	
30–39	5879 (12·3)	11 699 (12·7)		925 (7·1)	1834 (7·7)	
40–49	8041 (16·8)	15 882 (17·3)		1514 (11·7)	2940 (12·3)	
50–59	8953 (18·8)	17 620 (19·2)		2237 (17·3)	4481 (18·8)	
60–69	6221 (13·0)	11 784 (12·8)		2146 (16·6)	4095 (17·1)	
70–79	5321 (11·1)	9652 (10·5)		2515 (19·4)	4403 (18·4)	
80–89	3719 (7·8)	6374 (6·9)		2021 (15·6)	3243 (13·6)	
≥90	1126 (2·4)	1956 (2·1)		643 (5·0)	1044 (4·4)	
Sex			0·002			0·021
Male	20 132 (42·2)	38 844 (42·3)		5480 (42·3)	10 368 (43·4)	
Female	27 605 (57·8)	53 034 (57·7)		7463 (57·7)	13 530 (56·6)	
Birth country			0·087			0·137
Sweden	37 607 (78·8)	69 317 (75·4)		10 450 (80·7)	18 124 (75·8)	
Nordic	1223 (2·6)	2201 (2·4)		456 (3·5)	756 (3·2)	
Europe	3084 (6·5)	6953 (7·6)		744 (5·7)	1768 (7·4)	
Other	5823 (12·2)	13 407 (14·6)		1293 (10·0)	3250 (13·6)	
Employment status			0·036			0·053
Employed	31 069 (65·1)	61 146 (66·6)		6398 (49·4)	12 421 (52·0)	
Unemployed	14 422 (30·2)	26 260 (28·6)		6337 (49·0)	11 073 (46·3)	
Unknown	2246 (4·7)	4472 (4·9)		208 (1·6)	404 (1·7)	
Education level			0·018			0·018
Primary	9397 (19·7)	17 445 (19·0)		3408 (26·3)	6114 (25·6)	
Secondary	19 470 (40·8)	37 752 (41·1)		5466 (42·2)	10 160 (42·5)	
Higher	15 699 (32·9)	30 487 (33·2)		3628 (28·0)	6819 (28·5)	
Unknown	3171 (6·6)	6194 (6·7)		441 (3·4)	805 (3·4)	
Comorbidities						
Pulmonary embolism	1536 (3·2)	2643 (2·9)	0·020	768 (5·9)	1243 (5·2)	0·032
Stroke	1606 (3·4)	2780 (3·0)	0·019	793 (6·1)	1366 (5·7)	0·017
Deep vein thrombosis	278 (0·6)	542 (0·6)	0·001	87 (0·7)	154 (0·6)	0·003
Hypertension	12 570 (26·3)	23 230 (25·3)	0·024	5753 (44·4)	10 133 (42·4)	0·041
Ischaemic heart disease	4054 (8·5)	7209 (7·8)	0·024	1907 (14·7)	3201 (13·4)	0·039
Overall CV disease	18 019 (37·7)	31 289 (34·1)	0·077	7751 (59·9)	12 982 (54·3)	0·113
Heart failure	3989 (8·4)	7060 (7·7)	0·025	2033 (15·7)	3442 (14·4)	0·036
Asthma	7625 (16·0)	4507 (4·9)	0·368	1935 (15·0)	1591 (6·7)	0·270
COPD	3403 (7·1)	6444 (7·0)	0·004	1500 (11·6)	2769 (11·6)	0·001

For peer review only

Emphysema	354 (0·7)	404 (0·4)	0·039	185 (1·4)	190 (0·8)	0·061
Diabetes mellitus	5098 (10·7)	9671 (10·5)	0·005	2431 (18·8)	4336 (18·1)	0·016
Rheumatic disease	5523 (11·6)	7075 (7·7)	0·131	3557 (27·5)	4843 (20·3)	0·170
Cancer	7171 (15·0)	13 624 (14·8)	0·005	3219 (24·9)	6235 (26·1)	0·028
Prescribed medications						
Inhaled corticosteroids	17 363 (36·4)	14 069 (15·3)	0·496	4061 (31·4)	4602 (19·3)	0·281
Oral anticoagulants	8017 (16·8)	14 677 (16·0)	0·022	3617 (27·9)	6288 (26·3)	0·037
Statins	10 571 (22·1)	19 209 (20·9)	0·030	4240 (32·8)	7728 (32·3)	0·009
ACEIs	6601 (13·8)	12 781 (13·9)	0·002	2658 (20·5)	5130 (21·5)	0·023
Opioids	12 026 (25·2)	14 517 (15·8)	0·234	4284 (33·1)	5064 (21·2)	0·270

Data are n (%) or mean (SD). ACEI, angiotensin-converting enzyme inhibitor; COPD, chronic obstructive pulmonary disease; CV, cardiovascular; GC, glucocorticoid; SMD, standardised mean difference. *One or more prescriptions of oral GCs within 12 months before the COVID-19 infection date. †Two or more prescriptions and prednisolone ≥750 mg or equivalent within 6 months before COVID-19 infection date. Note this group is a subset of any prior exposure.

For peer review only

Supplementary Table S5: **Outcome events in propensity score matched cohorts of individuals with COVID-19 infection in the Swedish population during the period 2015 to COVID-19 infection date until Nov 30, 2021 stratified by level of exposure to oral glucocorticoids (non-exposed, high exposure in the 6 months before infection, or any exposure 12 months before infection and overall)**

	No=0/Yes=1	Any GC exposure and matched non-exposed			High GC exposure and matched non-exposed		
		Any GC exposure* (n=47 737)	Matched non-exposed (n=91 878)	Total any exposure and matched non-exposed (n=139 615)	High GC exposure† (n=12 943)	Matched non-exposed (n=23 898)	Total high exposure and matched non-exposed (n=36 841)
All-cause mortality							
	0	44 573 (93·4%)	87 973 (95·7%)	132 546 (94·9%)	11 054 (85·4%)	22 024 (92·2%)	33 078 (89·8%)
	1	3164 (6·6%)	3905 (4·3%)	7069 (5·1%)	1889 (14·6%)	1874 (7·8%)	3763 (10·2%)
Death from pulmonary embolism							
	0	47 598 (99·7%)	91 691 (99·8%)	139 289 (99·8%)	12 867 (99·4%)	23 804 (99·6%)	36 671 (99·5%)
	1	139 (0·3%)	187 (0·2%)	326 (0·2%)	76 (0·6%)	94 (0·4%)	170 (0·5%)
Death from sepsis							
	0	47 467 (99·4%)	91 627 (99·7%)	139 094 (99·6%)	12 799 (98·9%)	23 786 (99·5%)	36 585 (99·3%)
	1	270 (0·6%)	251 (0·3%)	521 (0·4%)	144 (1·1%)	112 (0·5%)	256 (0·7%)
Death from myocardial infarction							
	0	47 646 (99·8%)	91 718 (99·8%)	139 364 (99·8%)	12 889 (99·6%)	23 830 (99·7%)	36 719 (99·7%)
	1	91 (0·2%)	160 (0·2%)	251 (0·2%)	54 (0·4%)	68 (0·3%)	122 (0·3%)
Death from stroke							
	0	475 63 (99·6%)	91 592 (99·7%)	139 155 (99·7%)	12 845 (99·2%)	23 752 (99·4%)	36 597 (99·3%)
	1	174 (0·4%)	286 (0·3%)	460 (0·3%)	98 (0·8%)	146 (0·6%)	244 (0·7%)
Death from COVID-19							
	0	44 904 (94·1%)	88 387 (96·2%)	133 291 (95·5%)	11 240 (86·8%)	22 219 (93·0%)	33459 (90·8%)
	1	2833 (5·9%)	3491 (3·8%)	6324 (4·5%)	1703 (13·2%)	1679 (7·0%)	3382 (9·2%)
Hospitalisation for COVID-19							
	0	37 981 (79·6%)	77 727 (84·6%)	115 708 (82·9%)	8252 (63·8%)	17 810 (74·5%)	26 062 (70·7%)
	1	9756 (20·4%)	14 151 (15·4%)	23 907 (17·1%)	4691 (36·2%)	6088 (25·5%)	10 779 (29·3%)
ICU admission for COVID-19							
	0	46 736 (97·9%)	90 383 (98·4%)	137 119 (98·2%)	12 470 (96·3%)	23 320 (97·6%)	35 790 (97·1%)
	1	1001 (2·1%)	1495 (1·6%)	2496 (1·8%)	473 (3·7%)	578 (2·4%)	1051 (2·9%)

GC, glucocorticoid; ICU, intensive care unit. *One or more prescriptions of oral GCs within 12 months before the COVID-19 infection date. †Two or more prescriptions and prednisolone ≥750 mg of or equivalent within 6 months before COVID-19 infection date. Note this group is a subset of any prior exposure.

For peer review only

Supplementary Table S6: **Unadjusted and adjusted hazard ratios from Cox regression analyses and propensity score matched analysis with 95% confidence intervals comparing the risk of various causes of death and all-cause mortality between COVID-19 patients who had any or high prior exposure to oral glucocorticoids and non-exposed individuals**

Outcomes and method	Any prior GC exposure*		High GC exposure†	
	HR (95% CI)	No. of events	HR (95% CI)	No. of events
All-cause mortality				
Unadjusted Cox regression	5·46 (5·26–5·67)	18 228	12·33 (11·77–12·92)	16 873
Adjusted Cox regression‡	1·58 (1·52–1·65)	18 228	1·98 (1·87–2·09)	16 873
PS matching¶	1·47 (1·40–1·54)	7069	1·83 (1·71–1·95)	3763
Death from pulmonary embolism				
Unadjusted Cox regression	6·29 (5·26–7·52)	730	12·68 (10·04–16·03)	658
Adjusted Cox regression‡	1·33 (1·11–1·59)	730	1·37 (1·09–1·74)	658
PS matching¶	1·32 (1·05–1·65)	326	1·52 (1·12–2·06)	170
Death from sepsis				
Unadjusted Cox regression	8·67 (7·57–9·94)	1057	18·72 (15·79–22·19)	933
Adjusted Cox regression‡	2·35 (2·01–2·74)	1057	2·90 (2·37–3·54)	933
PS matching ¶	1·92 (1·61–2·29)	521	2·40 (1·86–3·09)	256
Death from stroke				
Unadjusted Cox regression	3·47 (2·96–4·05)	1376	7·22 (5·89–8·85)	1296
Adjusted Cox regression‡	1·16 (0·98–1·37)	1376	1·33 (1·05–1·67)	1296
PS matching¶	1·08 (0·89–1·31)	460	1·23 (0·94–1·59)	244
Death from myocardial infarction				
Unadjusted Cox regression	3·84 (3·10–4·75)	691	8·23 (6·27–10·82)	649
Adjusted Cox regression‡	1·04 (0·82–1·31)	691	1·36 (1·00–1·86)	649
PS matching¶	1·02 (0·79–1·33)	251	1·48 (1·03–2·13)	122
Death from COVID–19				
Unadjusted Cox regression	5·43 (5·22–5·65)	16 496	12·34 (11·75–12·96)	15 290
Adjusted Cox regression‡	1·57 (1·50–1·64)	16 496	1·96 (1·85–2·08)	15 290
PS matching¶	1·48 (1·41–1·56)	6324	1·84 (1·72–1·97)	3382
Secondary outcomes				
Hospitalisation for COVID–19				
Unadjusted Cox regression	4·34 (4·25–4·43)	72 982	8·52 (8·28–8·77)	67 596
Adjusted Cox regression‡	1·42 (1·39–1·45)	72 982	1·51 (1·46–1·57)	67 596
PS matching¶	1·28 (1·25–1·31)	23 907	1·44 (1·38–1·49)	10 779
ICU admission for COVID–19				
Unadjusted Cox regression	3·72 (3·49–3·97)	7896	6·41 (5·85–7·03)	7330
Adjusted Cox regression‡	1·33 (1·24–1·43)	7896	1·40 (1·26–1·55)	7330
PS matching¶	1·24 (1·15–1·35)	2496	1·52 (1·34–1·72)	1051

CI, confidence interval; GC, glucocorticoid; HR, hazard ratio; PS, propensity score. *One or more prescriptions of oral GCs within 12 months before the COVID-19 infection date. †Two or more prescriptions and prednisolone ≥750 mg or equivalent within 6 months before COVID-19 infection date. Note high exposure is a subset of any prior exposure. ‡Adjusted for covariates: age, sex, education, employment, and previous medical history (diabetes, deep vein thrombosis, pulmonary embolism, hypertension, stroke, ischaemic heart disease, heart failure, cancer, chronic obstructive pulmonary disease, rheumatologic conditions, and oral anticoagulation therapy. ¶1:2 matched on a PS score based on 15 covariates: age, sex, education, employment, and previous medical history (diabetes, deep vein thrombosis, pulmonary embolism, hypertension, stroke, ischaemic heart disease, heart failure, cancer, chronic obstructive pulmonary disease, rheumatologic conditions, oral anticoagulation therapy).

For peer review only - http://bmjopen.bmj.com/site/about/guidelines.xhtml

Supplementary Table S7: **Standardised mean differences between the 15 covariates of individuals with COVID-19 infections in the Swedish population during the period 2015 to COVID-19 infection date (comorbidities) or 1 year before COVID-19 infection date (medications) in those exposed to oral glucocorticoids compared with those non-exposed before and after propensity score matching**

Variable	Any prior GC exposure*		High GC exposure†	
	SMD before PS matching	SMD after PS matching	SMD before PS matching	SMD after PS matching
Age categories	0·672	0·047	1·276	0·081
Sex	0·149	0·002	0·147	0·021
Education	0·300	0·018	0·501	0·018
Employment	0·453	0·036	0·888	0·053
Diabetes mellitus	0·194	0·020	0·310	0·032
Deep vein thrombosis	0·161	0·019	0·282	0·017
Pulmonary embolism	0·088	0·001	0·098	0·003
Hypertension	0·547	0·024	0·972	0·041
Stroke	0·292	0·024	0·487	0·039
Ischaemic heart disease	0·341	0·025	0·561	0·036
Heart failure	0·356	0·004	0·488	0·001
Cancer	0·289	0·005	0·510	0·016
COPD	0·510	0·131	0·897	0·170
Rheumatic disease	0·406	0·005	0·653	0·028
Oral anticoagulants	0·413	0·022	0·689	0·037

COPD, chronic obstructive pulmonary disease; GC, glucocorticoid; PS, propensity score; SMD, standardised mean difference. *One or more prescriptions of oral GCs within 12 months before the COVID-19 infection date. †Two or more prescriptions and a total of prednisolone ≥750 mg or equivalent within 6 months before COVID-19 infection date. Note this group is a subset of any prior exposure.

For peer review only

Supplementary Figure S1: **Propensity score balance before (a, c) and after (b, d) performing propensity score matching on all 15 covariates for individuals with COVID-19 infections in the Swedish population between 2015 and the COVID-19 infection date comparing those exposed to oral glucocorticoids (pink) with those who were non-exposed (blue) in those with any (a, b) or high glucocorticoid exposure (c, d)**

GC, glucocorticoid; PS, propensity score. Any prior GC exposure equals ≥1 prescription of oral GC within 12 months before the COVID-19 infection date. High GC exposure equals ≥2 prescriptions with a total of ≥750 mg of prednisolone or equivalent. The latter group is a subset of any prior exposure. Note: The figure includes PS density plots and histograms illustrating the distribution of PS values. Prior to matching, the PS density plots and histograms represent the distribution of PS values for both exposed and non-exposed individuals. Subsequently, a 1:2 matching scheme was employed using a caliper of 0.2 where each exposed individual was paired with two non-exposed individuals based on their similarity in PS values. The PS density plots and histograms after matching reflect the distribution of PS values for the matched groups, demonstrating the achieved balance in the covariates. The 15 covariates used in the logistic model to estimate the propensity score can be found in Supplementary Table S7.

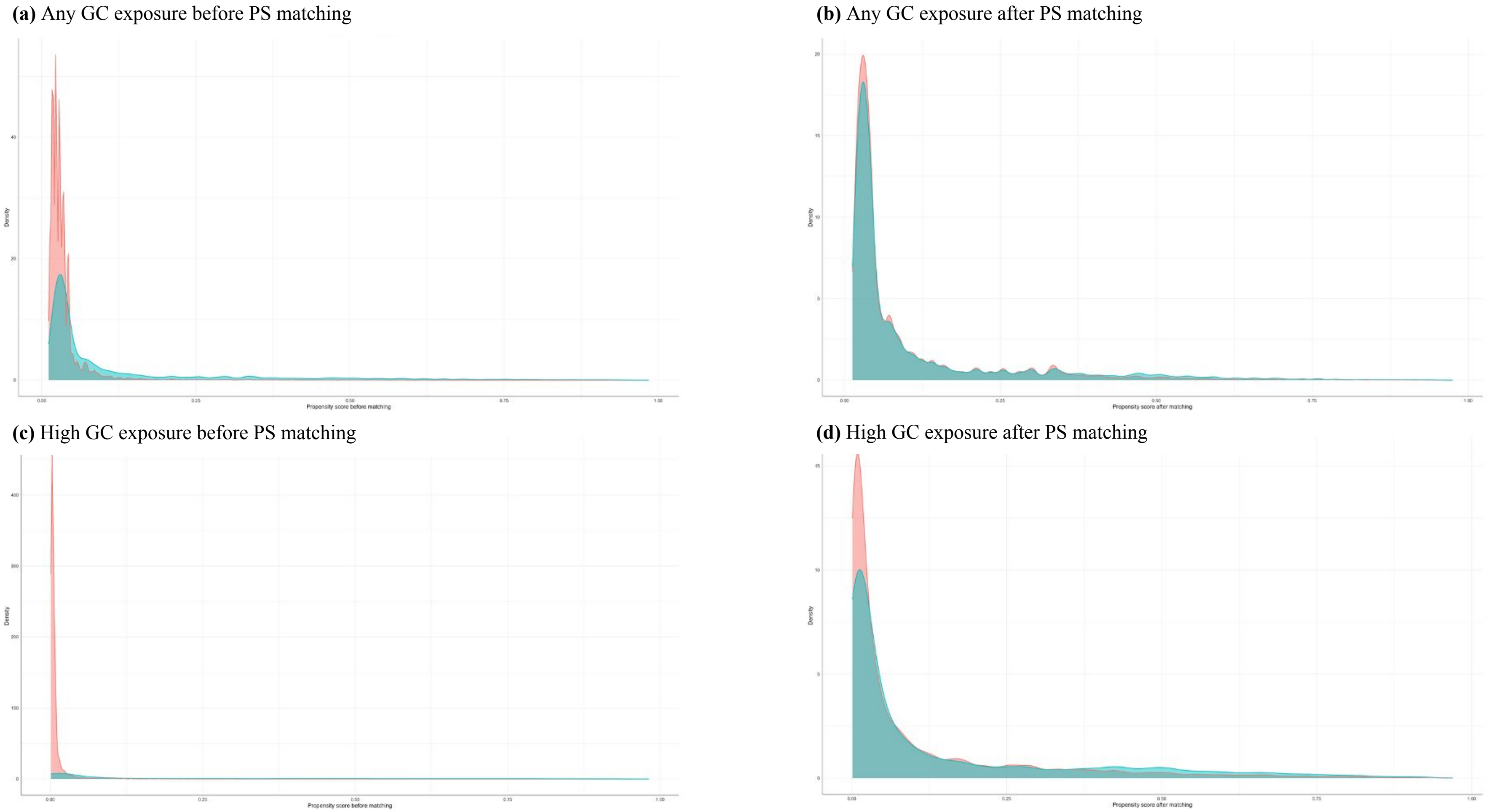

Supplementary Figure S2: **Standardised mean differences between the covariates of individuals with COVID-19 infections in the Swedish population during the period 2015 to COVID-19 infection date (comorbidities) or 1 year before COVID-19 infection date (medications) in the exposed to oral glucocorticoids compared with non-exposed before (red circles) and after (blue triangles) propensity score matching among those with (a) any or (b) high glucocorticoid exposure.** COPD, chronic obstructive pulmonary disease; GC, glucocorticoid. Any prior exposure equals ≥1 prescription of oral glucocorticoids within 12 months before the COVID-19 infection date. High exposure equals ≥2 prescriptions of oral glucocorticoids with a total of ≥750 mg of prednisolone or equivalent within 6 months before COVID-19 infection date. Note the latter group is a subset of any prior exposure.

(a) Any GC exposure **(b)** High GC exposure

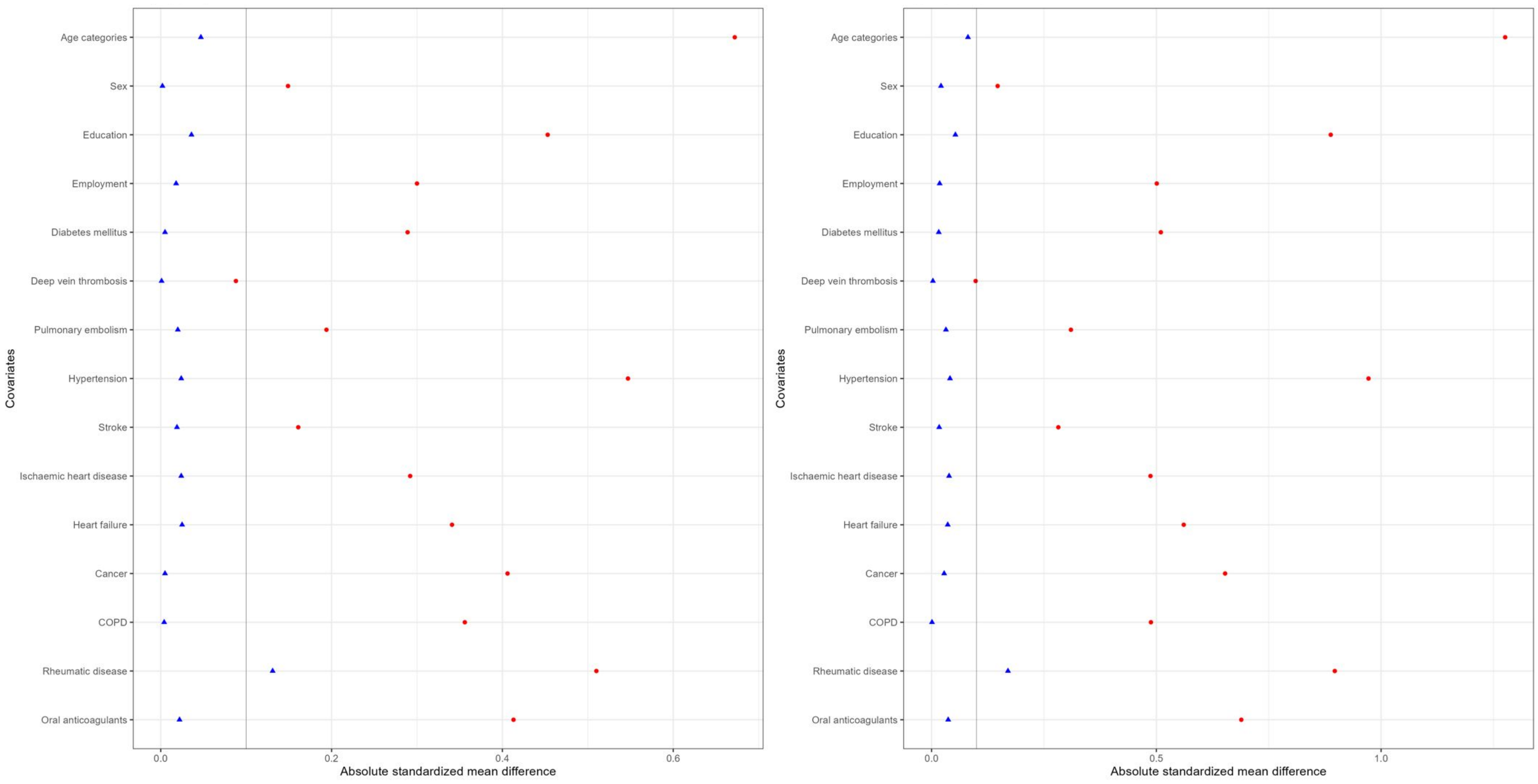

STROBE Statement—Checklist of items that should be included in reports of ***cohort studies***

	Item No	Recommendation
**Title and abstract**	1	(*a*) Indicate the study's design with a commonly used term in the title or the abstract **Page 1**
		(*b*) Provide in the abstract an informative and balanced summary of what was done and what was found **Page 5-6**
**Introduction**		
Background/rationale	2	Explain the scientific background and rationale for the investigation being reported **Page 7-9**
Objectives	3	State specific objectives, including any prespecified hypotheses **Page 9**
**Methods**		
Study design	4	Present key elements of study design early in the paper **Page 9-12**
Setting	5	Describe the setting, locations, and relevant dates, including periods of recruitment, exposure, follow-up, and data collection **Page 9-12**
Participants	6	(*a*) Give the eligibility criteria, and the sources and methods of selection of participants. Describe methods of follow-up **Page 9-12**
		(*b*) For matched studies, give matching criteria and number of exposed and unexposed **Page 12-15**
Variables	7	Clearly define all outcomes, exposures, predictors, potential confounders, and effect modifiers. Give diagnostic criteria, if applicable **Page 10-12**
Data sources/ measurement	8*	For each variable of interest, give sources of data and details of methods of assessment (measurement). Describe comparability of assessment methods if there is more than one group **Page 9-12**
Bias	9	Describe any efforts to address potential sources of bias **Page 12**
Study size	10	Explain how the study size was arrived at **Page 9**
Quantitative variables	11	Explain how quantitative variables were handled in the analyses. If applicable, describe which groupings were chosen and why **Page 12**
Statistical methods	12	(*a*) Describe all statistical methods, including those used to control for confounding **Page 12**
		(*b*) Describe any methods used to examine subgroups and interactions
		(*c*) Explain how missing data were addressed **No missing data reported**
		(*d*) If applicable, explain how loss to follow-up was addressed
		(*e*) Describe any sensitivity analyses **Page 14-15**
**Results**		
Participants	13*	(a) Report numbers of individuals at each stage of study—eg numbers potentially eligible, examined for eligibility, confirmed eligible, included in the study, completing follow-up, and analysed **Page 13**
		(b) Give reasons for non-participation at each stage
		(c) Consider use of a flow diagram
Descriptive data	14*	(a) Give characteristics of study participants (eg demographic, clinical, social) and information on exposures and potential confounders **Table 1**
		(b) Indicate number of participants with missing data for each variable of interest **No missing data reported**
		(c) Summarise follow-up time (eg, average and total amount) **Page 10-11**
Outcome data	15*	Report numbers of outcome events or summary measures over time **Supplementary table S5 and S6**
Main results	16	(*a*) Give unadjusted estimates and, if applicable, confounder-adjusted estimates and

For peer review only

		their precision (eg, 95% confidence interval). Make clear which confounders were adjusted for and why they were included **Supplementary table S6**
		(*b*) Report category boundaries when continuous variables were categorized **Supplementary Table S2**
		(*c*) If relevant, consider translating estimates of relative risk into absolute risk for a meaningful time period
Other analyses	17	Report other analyses done—eg analyses of subgroups and interactions, and sensitivity analyses **Page 14-15**
**Discussion**		
Key results	18	Summarise key results with reference to study objectives **Page 15**
Limitations	19	Discuss limitations of the study, taking into account sources of potential bias or imprecision. Discuss both direction and magnitude of any potential bias **Page 17-18**
Interpretation	20	Give a cautious overall interpretation of results considering objectives, limitations, multiplicity of analyses, results from similar studies, and other relevant evidence **Page 15-19**
Generalisability	21	Discuss the generalisability (external validity) of the study results **Page 18**
**Other information**		
Funding	22	Give the source of funding and the role of the funders for the present study and, if applicable, for the original study on which the present article is based **Page 5**

*Give information separately for exposed and unexposed groups.

Note: An Explanation and Elaboration article discusses each checklist item and gives methodological background and published examples of transparent reporting. The STROBE checklist is best used in conjunction with this article (freely available on the Web sites of PLoS Medicine at http://www.plosmedicine.org/, Annals of Internal Medicine at http://www.annals.org/, and Epidemiology at http://www.epidem.com/). Information on the STROBE Initiative is available at http://www.strobe-statement.org.

For peer review only

For peer review only - http://bmjopen.bmj.com/site/about/guidelines.xhtml

BMJ Open

Impact of chronic oral glucocorticoid treatment on mortality in patients with COVID-19: analysis of a population-based cohort

Journal:	*BMJ Open*
Manuscript ID	bmjopen-2023-080640.R1
Article Type:	Original research
Date Submitted by the Author:	12-Jan-2024
Complete List of Authors:	Einarsdottir, Margret; University of Gothenburg, Department of Internal Medicine and Clinical Nutrition; Sahlgrenska University Hospital, Department of Endocrinology<br>Kibiwott Kirui, Brian; University of Gothenburg, School of Public Health and Community Medicine, Institute of Medicine, Sahlgrenska Academy<br>Li, Huiqi; University of Gothenburg School of Public Health and Community Medicine<br>Olsson, Daniel; University of Gothenburg, Department of Internal Medicine and Clinical Nutrition; AstraZeneca<br>Johannsson, Gudmundur; University of Gothenburg, Department of Internal Medicine and Clinical Nutrition; Sahlgrenska University Hospital, Department of Endocrinology<br>Nyberg, Fredrik; University of Gothenburg School of Public Health and Community Medicine<br>Ragnarsson, Oskar; University of Gothenburg, Department of Internal Medicine and Clinical Nutrition; Sahlgrenska University Hospital, Department of Endocrinology
**Primary Subject Heading**:	Epidemiology
Secondary Subject Heading:	Epidemiology
Keywords:	Mortality, COVID-19, Adrenal disorders < DIABETES & ENDOCRINOLOGY

SCHOLARONE™
Manuscripts

I, the Submitting Author has the right to grant and does grant on behalf of all authors of the Work (as defined in the below author licence), an exclusive licence and/or a non-exclusive licence for contributions from authors who are: i) UK Crown employees; ii) where BMJ has agreed a CC-BY licence shall apply, and/or iii) in accordance with the terms applicable for US Federal Government officers or employees acting as part of their official duties; on a worldwide, perpetual, irrevocable, royalty-free basis to BMJ Publishing Group Ltd ("BMJ") its licensees and where the relevant Journal is co-owned by BMJ to the co-owners of the Journal, to publish the Work in this journal and any other BMJ products and to exploit all rights, as set out in our licence.

The Submitting Author accepts and understands that any supply made under these terms is made by BMJ to the Submitting Author unless you are acting as an employee on behalf of your employer or a postgraduate student of an affiliated institution which is paying any applicable article publishing charge ("APC") for Open Access articles. Where the Submitting Author wishes to make the Work available on an Open Access basis (and intends to pay the relevant APC), the terms of reuse of such Open Access shall be governed by a Creative Commons licence – details of these licences and which Creative Commons licence will apply to this Work are set out in our licence referred to above.

Other than as permitted in any relevant BMJ Author's Self Archiving Policies, I confirm this Work has not been accepted for publication elsewhere, is not being considered for publication elsewhere and does not duplicate material already published. I confirm all authors consent to publication of this Work and authorise the granting of this licence.

For peer review only

Impact of chronic oral glucocorticoid treatment on mortality in patients with COVID-19: analysis of a population-based cohort

Running title: Glucocorticoids, mortality and COVID-19

Authors: Margrét J Einarsdóttir (0000-0002-6010-9599)[1,2*], Brian K Kirui (0000-0002-6656-6029)[3*], Huiqi Li (0000-0002-1127-0829) [3], Daniel S Olsson (0000-0002-9734-0786)[1,2,5], Gudmundur Johannsson (0000-0003-3484-8440)[1,2], Fredrik Nyberg (0000-0003-0892-5668)[3†], Oskar Ragnarsson (0000-0003-0204-9492)[1,2,4†]

*Joint first authors

†Joint senior authors

Affiliations: [1]Department of Internal Medicine and Clinical Nutrition, Institute of Medicine, Sahlgrenska Academy, University of Gothenburg, Gothenburg, Sweden; [2]Department of Endocrinology, Sahlgrenska University Hospital, Gothenburg, Sweden; [3]School of Public Health and Community Medicine, Institute of Medicine, Sahlgrenska Academy, University of Gothenburg, Gothenburg, Sweden; [4]Wallenberg Center for Molecular and Translational Medicine, University of Gothenburg, Gothenburg, Sweden; [5]Cardiovascular, Renal and Metabolism (CVRM), BioPharmaceuticals R&D, AstraZeneca, Gothenburg, Sweden.

Address for correspondence and reprint requests: Oskar Ragnarsson, Department of Internal Medicine and Clinical Nutrition, Institute of Medicine,

For peer review only

Sahlgrenska Academy, University of Gothenburg, 405 30 Gothenburg, Sweden; Tel: +46 70 728 2229; E-mail: oskar.ragnarsson@medic.gu.se

Manuscript category: Original article

Abstract length: 247

Text length: 3350

No. of references: 30

No. of graphics: 4 (1 table and 3 figures)

Supplementary data: Appendix provided as separate file

Author addresses:

Margrét J Einarsdóttir, Department of Internal Medicine and Clinical Nutrition, Institute of Medicine, Sahlgrenska Academy, University of Gothenburg, 405 30 Gothenburg, Sweden; Tel: +46313427056; E-mail: margret.jona.einarsdottir@gu.se

Brian K Kirui, School of Public Health and Community Medicine, Institute of Medicine, Sahlgrenska Academy, University of Gothenburg, Gothenburg, Sweden; Tel: +46700579203; E-mail: brian.kirui@gu.se

Huiqi Li, School of Public Health and Community Medicine, Institute of Medicine, Sahlgrenska Academy, University of Gothenburg, Gothenburg, Sweden; Tel: +46317866251; E-mail: huiqi.li@gu.se

For peer review only

Daniel S Olsson, Department of Internal Medicine and Clinical Nutrition, Institute of Medicine, Sahlgrenska Academy, University of Gothenburg, 405 30 Gothenburg, Sweden; Tel: +46708278671; E-mail: daniel.olsson@gu.se

Gudmundur Johannsson, Department of Internal Medicine and Clinical Nutrition, Institute of Medicine, Sahlgrenska Academy, University of Gothenburg, 405 30 Gothenburg, Sweden; Tel: +46313427052 E-mail: gudmundur.johannsson@medic.gu.se

Fredrik Nyberg, School of Public Health and Community Medicine, Institute of Medicine, Sahlgrenska Academy, University of Gothenburg, Gothenburg, Sweden; Tel: +46 31 7866289; E-mail: fredrik.nyberg.2@gu.se

Oskar Ragnarsson, Department of Internal Medicine and Clinical Nutrition, Institute of Medicine, Sahlgrenska Academy, University of Gothenburg, 405 30 Gothenburg, Sweden; Tel: +46 70 728 2229; E-mail: oskar.ragnarsson@medic.gu.se

For peer review only

Ethics approval statement

Ethical approval for this study was obtained from the Swedish Ethical Review Authority (2020-01800 with subsequent amendments).

Abstract

Objectives:

While glucocorticoid (GC) treatment initiated for COVID-19 reduces mortality, it is unclear whether GC treatment prior to COVID-19 affects mortality. Long-term GC use raises infection and thromboembolic risks. We investigated if patients with oral GC use prior to COVID-19 had increased mortality overall and by selected causes.

Design: Population-based observational cohort study

Settings: Population-based register data in Sweden

Participants: All patients infected with COVID-19 in Sweden from January 2020 to November 2021 (n=1,200,153).

Outcome measures: Any prior oral GC use was defined as ≥1 GC prescription during 12 months before index. High exposure was defined as ≥2 GC prescriptions with a cumulative prednisolone dose ≥750 mg or equivalent during 6 months before index. GC users were compared with COVID-19 patients who had not received GCs within 12 months before index. We used Cox proportional hazard models and 1:2 propensity score matching to estimate HRs and 95% CIs, controlling for the same confounders in all analyses.

Results:

3378 deaths occurred in subjects with any prior GC exposure (n=48,806; 6·9%) and 14,850 among non-exposed (n=1,151,347; 1·3%). Both high (1·98, 1·87–2·09) and any exposure (1·58, 1·52–1·65) to GCs were associated with overall death. Deaths from pulmonary embolism, sepsis, and COVID-19 were associated with high GC

For peer review only

exposure and, similarly but weaker, with any exposure. High exposure to GCs was associated with increased deaths caused by stroke and myocardial infarction.

Conclusion:

Patients on oral GC treatment prior to COVID-19 have increased mortality, particularly from pulmonary embolism, sepsis, and COVID-19.

Key words: glucocorticoids, mortality, adrenal insufficiency, COVID-19

Strengths and limitations of this study

- Utilizes a population-based design
- First study to investigate if oral glucocorticoid exposure prior to COVID-19 affects mortality
- Employs adjusted Cox models and propensity score matching for confounder control. A large number of covariates, and a sizable comparison cohort.
- Observational nature may still introduce residual bias and unmeasured confounding.

For peer review only

INTRODUCTION

Oral glucocorticoids (GCs) are commonly prescribed for a variety of conditions, including inflammatory disorders as well as various allergic, respiratory, skin, and autoimmune conditions (1). In Sweden, 0.5% of the population receive long-term (>2 years) oral GC treatment (1), a treatment that is known to be associated with excess mortality mainly due to myocardial infarction, sepsis, and pulmonary embolism (2). Hypercortisolism is associated with a hypercoagulable state due to increased levels of procoagulant factors and impaired fibrinolysis (3). Patients receiving long-term GC treatment frequently also develop GC-induced adrenal insufficiency that renders them at increased risk of adrenal crisis, and subsequent death, during a severe infection (4).

Thromboembolic events, including pulmonary embolism and stroke, are common complications in patients with COVID-19 (5). Despite anticoagulation treatment, a notable proportion (16·7%) of patients with COVID-19 develop pulmonary embolism (5). COVID-19 patients may also develop arterial thrombosis, which can lead to serious outcomes such as stroke or myocardial infarction (6).

The use of oral GCs in COVID-19 patients has been a topic of research and debate. Current literature on GC therapy initiated for COVID-19 and effect on thromboembolic events warns that GC administration during COVID-19 may increase the risk of thromboembolic events, particularly in critically ill patients or those with pre-existing thrombotic risk factors (7). In May 2020, WHO published clinical guidelines on the management of COVID-19 and advised against the use of

For peer review only

GCs unless indicated for another reason (eg, exacerbation of asthma or chronic obstructive pulmonary disease) (8). At that time, high-dose GC therapy initiated for COVID-19 had been associated with delayed virus clearing, prolonged hospitalisation, and increased use of mechanical ventilation in patients with COVID-19 (9). In July 2020, the RECOVERY trial showed that dexamethasone treatment initiated in COVID-19 patients resulted in lower 28-day mortality among those requiring respiratory support (mechanical ventilation or oxygen alone) (10). Consequently, in September 2020, WHO revised the guidelines and recommended initiation of systemic GC treatment for patients with severe and critical COVID-19 (11). Nevertheless, GC treatment for COVID-19 patients is still controversial. GC therapy initiated for COVID-19 was related to increased death compared with conventional treatment in many observational studies, while beneficial effects on mortality were seen in randomised clinical trials (12). There is also a concern regarding the dose of GC therapy initiated for COVID-19, as higher doses have been shown to increase mortality rates in hypoxic patients not requiring ventilatory support (13). In the aforementioned studies, GC treatment was initiated for the treatment of COVID-19.

There is less known about how already present long-term GC treatment prior to COVID-19 effects outcome and mortality. An international study of patients with rheumatic disease receiving long-term GC treatment showed that prednisolone ≥10 mg/day prior to COVID-19 was associated with an increased hospitalisation rate (14). Chronic systemic GC therapy has also been associated with increased mortality in immunosuppressed patients with COVID-19 (15). Whether patients

For peer review only

receiving GC treatment for other conditions are at increased risk of death after developing COVID-19 has not been systematically studied.

Our hypothesis was that patients on oral GC treatment before being diagnosed with COVID-19 have an increased mortality from several conditions, including pulmonary embolism, myocardial infarction, stroke, sepsis, and COVID-19 itself compared with those who do not have GC treatment before COVID-19. To test this hypothesis, we investigated the association between prior GC treatment and the risk of mortality from these conditions among COVID-19 patients using population-based analysis.

METHODS

Study design, data sources, and study population

This was a register-based cohort study centred on the regularly updated SCIFI-PEARL (Swedish COVID-19 Investigation for Future Insights – a Population Epidemiology Approach using Register Linkage) project database,(16) which currently includes the entire Swedish population from 2015.

The study population consisted of individuals with COVID-19 who met the following criteria before Nov 30, 2021: (a) a positive test for SARS-CoV-2 in the National Database of Notifiable Communicable Diseases (SmiNet); (b) identified in the Swedish National Patient Register (NPR) with the International Classification of Diseases, revision 10 (ICD-10) codes U07.1 or U07.2 from a specialist visit or hospitalisation; or (c) identified in the Cause-of-Death Register with COVID-19 as an underlying or contributing cause of death. For each individual, their earliest date of

For peer review only

meeting any of these COVID-19 diagnosis criteria was considered their index date (baseline) in the study.

Additional data and sources used from the SCIFI-PEARL database included: socio-demographic data and dates of emigration or death from Statistics Sweden (Register of Total Population and the Longitudinal Integrated Database for Health Insurance and Labor Market Studies [LISA]) (17); prescribed medication data from the National Prescribed Drug Register (NPDR) (18); comorbidities from the NPR;(19) and intensive care data from the Swedish Intensive Care Register (SIR). Comprehensive data for each patient were obtained by linking their information across the different registers using the national personal identification number, as previously described (16). Ethical approval for this study was obtained from the Swedish Ethical Review Authority (2020-01800 with subsequent amendments).

Patient and Public Involvement: None

Exposures, outcomes, and follow-up

Data from the NPDR were used to identify oral GC users as well as GC dosage and formulation (capsules, drops, or tablets). Recent or ongoing oral GC treatment was defined in two ways: (a) any prior exposure as ≥1 dispensed prescriptions of oral GCs within 12 months before the index date; and (b) high exposure defined >2 dispensed prescriptions with a cumulative dose of prednisolone (Anatomical Therapeutic Chemical [ATC] code H02AB06) ≥750 mg, or equivalent amount (Supplementary Table S1) of betamethasone (H02AB01), dexamethasone (H02AB02), methylprednisolone (H02AB04), prednisone (H02AB07), hydrocortisone

For peer review only

(H02AB09), cortisone (H02AB10), deflazacort (H02AB13), or a combination of these within 6 months before the index date. All patients belonging to the "high exposure" group were thus also included in the "any prior exposure" group. These exposure definitions target recent use of oral GC, and for the “high exposure” more consistent and recent use, more likely extending into the future follow-up period, thus sharpening the identification of regular users and as such, patients at risk, and ensuring robust data analysis. The non-exposed comparison group included COVID-19 patients who had not received any oral GC prescriptions during the year before their index date.

The primary outcomes were overall death (all-cause mortality) and cause-specific death according to ICD-10 classification due to pulmonary embolism (I26), sepsis (A40, A41, or R57.2), myocardial infarction (I21–I23), stroke (I60–I64, I630–I635, I638, or I639), and COVID-19 (U07.1 or U07.2). Outcomes of cause-specific death were identified using the Cause-of-Death Register with the diagnosis being either the underlying or contributing cause. Secondary outcomes were two measures of severe COVID-19, hospitalisation for COVID-19, and direct admission to an intensive care unit, each defined by U07.1 or U07.2 as primary or secondary diagnosis, respectively, in the NPR or SIR. The different outcomes were all analysed separately. For each outcome, all subjects were followed from the index date (COVID-19 infection date) until the earliest of the following: an event for the specific outcome, emigration, death, or end of the study period (Nov 30, 2021).

Baseline covariates

For peer review only - http://bmjopen.bmj.com/site/about/guidelines.xhtml

The following baseline information was extracted from relevant registers (for details see Table S2): (1) demographic factors from Statistics Sweden including age, sex, education, and employment; (2) comorbidities from the NPR as primary or secondary diagnoses for specialist outpatient or inpatient care from Jan 1, 2015 until the index date; and (3) dispensed prescribed medications from the NPDR during the year before the index date. Based on *a priori* selection, the following covariates were used for confounding adjustment: age, sex, education, employment, and relevant previous medical history (diabetes, deep vein thrombosis, pulmonary embolism, hypertension, stroke, ischaemic heart disease, heart failure, cancer, chronic obstructive pulmonary disease, rheumatologic conditions, and oral anticoagulation therapy). In sensitivity analyses, we considered additional covariates: adrenal insufficiency, rheumatoid arthritis, chronic lower respiratory diseases, other respiratory diseases principally affecting the interstitium, non-infective enteritis and colitis, dermatitis and eczema, inflammatory polyarthropathies, systemic connective tissue disorders (except rheumatoid arthritis), glomerular diseases, organ or tissue transplant, chronic liver disease, alcoholism and other substance abuse, and the following medications: inhaled corticosteroids, statins, angiotensin converting enzyme inhibitors (ACEIs), and opioids, (Table S2).

Statistical analysis

The exposed and non-exposed groups were characterised using descriptive statistics with continuous variables presented as mean and standard deviation (SD), and categorical variables as number (%). Differences between exposed and non-exposed groups were characterised using standardised mean differences (SMDs). Kaplan-Meier curves were used to examine the outcomes over time during the study

For peer review only

period. Cox proportional hazards regression was used to obtain hazard ratios (HRs) with 95% confidence intervals (CIs). In the main analysis, confounding was addressed by adjusting for the selected potential confounders in standard Cox models. In an alternative analysis approach, confounding was addressed by 1:2 matching on a propensity score that included the same set of confounders as in the main regression analysis. The propensity score was estimated by fitting a logistic regression model with the selected covariates as predictors and oral GC exposure as independent variables. A 1:2 matching on the propensity score was then performed and evaluated by assessment of SMD pre- and post-matching for all variables and by visually checking the propensity score overlap. Sensitivity analyses were also conducted incorporating additional potential confounders of interest, in particular greater detail regarding the underlying indications for oral GC. This sensitivity analysis could, however, instead suffer from potential overadjustment in relation to the study hypothesis of understanding the overall increased risk patients in these groups on oral GC faced when infected with COVID-19. Analyses were performed in SAS, and R statistical software version 4.04 and matching was implemented using the MatchIT package in R (20).

For peer review only

RESULTS

Demographic and clinical characteristics

A cohort of 1 200 153 individuals with COVID-19 was identified with 48 806 having any GC exposure the year before index, including 13 493 individuals with high GC exposure during the last 6 months (table 1). The exposed groups were older and included more women, whereas the non-exposed group had a more even sex distribution. Substantial differences between the exposed and non-exposed (SMDs >0·2) were found for age, sex, and multiple comorbidities including cardiovascular, respiratory, cancer, and rheumatic disease (table 1). There were 3378 deaths in patients with any GC exposure (6·9%), of which 2023 deaths occurred in patients with high exposure (15·0%) and 14 850 deaths in the non-exposed group (1·3%) (Table S3).

Association between GC exposure and death

Unadjusted cumulative incidence curves indicated that crude overall and cause-specific mortality was higher among GC users compared with non-users (figures 1 and 2).

In the main Cox regression analyses, the adjusted HR for all-cause mortality was 1·98 (95% CI 1·87–2·09) in the high exposure group and 1·58 (1·52–1·65) in patients with any exposure compared with the non-exposed group (figure 3). High exposure to oral GCs was also associated with significantly increased risks of death from pulmonary embolism, sepsis, stroke, and COVID-19. Any prior exposure was

For peer review only

similarly associated with death due to pulmonary embolism, sepsis, and COVID-19, but not significantly with fatal stroke (figure 3). The association for high exposure with death from myocardial infarction was similar in strength to that with fatal stroke, but marginally not significant (figure 3).

Association between GC exposure and severe COVID-19

Compared with the non-exposed group, the high exposure group demonstrated an elevated risk of hospitalisation for COVID-19 (adjusted HR 1·51, 95% CI 1·46–1·57) as well as intensive care unit admission (1·40, 1·26–1·55) (figure 3). There were also consistent but relatively weaker effects observed in patients with any prior exposure.

Alternative analysis approach with propensity score matching

Propensity score matching resulted in 91 878 non-exposed individuals matched to 47 737 individuals with any exposure and 23 898 individuals matched to 12 943 individuals with high exposure (Table S4). The numbers of outcome events in the matched cohorts are indicated in Table S5. In the matched cohorts, the covariates of the exposed and matched non-exposed groups were quite well balanced (Table S6) with good overlap of the propensity scores (Supplementary Figure S1) and low absolute SMDs between the matched cohorts (Table S5 and S6, Figure S2).

The results obtained from the propensity score matched analysis were, overall, in good agreement with the adjusted Cox model (figure 3). Propensity score matching indicated somewhat stronger effects of GC exposure on pulmonary embolism and myocardial infarction death, and weaker effects on sepsis and stroke death, severe COVID-19, and overall mortality in both high and any exposure categories (figure 3).

For peer review only

In this analysis, high oral GC exposure was not significantly associated with death from stroke but, conversely, it was significantly and more strongly associated with myocardial infarction deaths.

In a post-hoc sensitivity analysis, we sought to address potential confounding due to slight remaining covariate imbalances through adjustment and propensity score matching, utilizing an expanded set of covariates. This led to improved balance across most covariates (Table S7). Overall, the findings closely aligned with the main analysis except that ICU admission for COVID-19 was less common in GC users in this analysis (Table S8).

DISCUSSION

This nationwide population-based cohort study of approximately 1·2 million individuals who had contracted COVID-19 infection showed increased mortality following the infection for 48 806 oral GC users compared with non-users, especially from sepsis, stroke, and pulmonary embolism. Individuals receiving oral GC treatment prior to the infection also had an increased risk of COVID-19-related outcomes including hospitalisation, intensive care unit admission, and death. Furthermore, the results were robust in sensitivity analyses with extended confounding adjustment.

Comparison to other studies

Our study showed an increased risk of death from sepsis after contracting COVID-19 and the risk was greater in the high GC exposure group. Treatment with GCs is known to cause immunosuppression and GC users have increased susceptibility to

For peer review only

infections (21). Similarly, studies on endogenous hypercortisolism and GC users show a high mortality risk due to infection in other settings (2 22). For example, one-third of deaths in patients with Cushing disease is due to infections (22) and GC users have generally increased mortality from sepsis and pneumonia (2). There is also evidence that higher doses of GCs are associated with an increased risk of pneumonia and mortality in COVID-19 patients hospitalised with clinical hypoxia compared with standard low-dose GC treatment (13). Our results are also in line with observational data on patients with rheumatic diseases, showing that prednisolone dose ≥10mg/day prior to COVID-19 was associated with increased hospitalisation for COVID-19 (14).

We also found increased mortality from pulmonary embolism in GC users after contracting COVID-19. A generally increased incidence of thromboembolism in patients with endogenous hypercortisolism is known (23). A recent study showed that patients with Cushing's syndrome have a generally increased incidence of stroke and thromboembolism (23). Studies on GC users have reported similar findings with an approximately two-fold increased risk of both pulmonary embolism and deep vein thrombosis (24). The risk of comparable events after COVID-19 in the current study is similar in magnitude.

GC treatment was not significantly associated with death from stroke in our study, but it was significantly and more strongly associated with myocardial infarction deaths. The association was stronger in the high exposure group compared with the any prior exposure group. Our findings are in line with a population-based, nested, case-control study from the UK on oral GC and risk of cardiovascular and

For peer review only - http://bmjopen.bmj.com/site/about/guidelines.xhtml

cerebrovascular disease based on data from 1988 to 1998 (25). The study showed increased risk of ischaemic heart disease (odds ratio [OR] 1·20, 95% CI 1·11–1·29) in current GC users, but not for increased risk of stroke/transient ischaemic attack (OR 0·95, 95% CI 0·89–1·01) (25).

We used both adjusted Cox models and propensity score matching to control for potential confounders in an effort to obtain unbiased estimates of the impact of oral GC use on severe outcomes. To clarify and understand imbalances in characteristics between the exposure groups before and after propensity score matching, we evaluated SMDs and achieved good balance after matching (26). It may be noted that the estimand for the adjusted Cox model is the conditional average treatment effect for the treated (ATE), ie, the effect if everyone were to be on treatment with oral GCs, conditional on their characteristics, while the estimand for the matched analysis is the average treatment effect for the treated (ATT), ie, the effect among those actually receiving oral GC treatment (27). ATE can be used to decide whether to mandate treatment for all patients, whereas ATT can be used to decide whether to withhold it from patients who would otherwise receive it. Nevertheless, the two methods produced comparable results that are clinically relevant and complementary.

Strengths and limitations of this study

This study has several strengths including a population-level sample, a large number of covariates, and a large comparison cohort which minimizes the risk of selection bias. However, since our study is observational, the analyses may still be subject to residual bias and unmeasured confounding, and thus the results require cautious

For peer review only

interpretation. All GC users have an underlying disease that may increase mortality risk, potentially confounding the results. We mitigated this concern by adjusting for carefully pre-selected covariates, by conducting a sensitivity analysis with expanded covariate selection, and by using two analytical approaches. The results of all analyses were fairly consistent, supporting the robustness of the findings. The sensitivity analyses, however, suggested that oral GC per se may not be the driver for increased COVID-19-related ICU admission, although this is speculative and should be interpreted with caution due to the more limited power for this rare outcome and the risk for overadjustment in these analyses.

With regard to stage of severity and exposure to GC, we assessed both "any" exposure and "high" exposure to address differences in the severity of the underlying condition warranting oral GC treatment. In our study, we relied on a prescription register that only provided information about dispensed prescriptions, and thus adherence to treatment and actual use must be assumed (as is generally the case). Our study exclusively focused on the oral administration of GCs and their impact on COVID-19. While inhaled GCs have been studied in Swedish context (28), our study places a deliberate emphasis on the oral route. Other administration routes for GC, such as topical and parenteral administrations, were intentionally excluded from our investigations, as they are likely less directly pertinent to COVID-19.

Unanswered questions and future research

The causal relationship between GC use and mortality remains a topic yet to be fully elucidated, primarily due to the fact that all long-term GC users have an underlying disease that may heighten mortality risk. Our study provides a comprehensive

For peer review only - http://bmjopen.bmj.com/site/about/guidelines.xhtml

overview of excess mortality in this population. However, it's crucial to highlight that studying each underlying medical condition necessitates specific analytical considerations. To enhance our understanding of the factors contributing to excess mortality, future research should prioritize dissecting the precise impact of prolonged GC use on these underlying conditions. Our mortality estimates essentially represent a weighted average effect over the pandemic period, primarily for power and precision reasons. Larger cohorts in future studies may help reveal wave-specific effects.

Fundamentally, GC treatment suppresses the hypothalamic-pituitary-adrenal axis and can cause GC-induced adrenal insufficiency (4 29). Undiagnosed and untreated GC-induced adrenal insufficiency can lead to a life-threatening adrenal crisis. To prevent adrenal crisis, GC users should know how to increase their GC dose during sick days and during intercurrent illness (30). Awareness of GC-induced adrenal insufficiency is increasing. In 2020, guidance on the prevention of adrenal crisis during the COVID-19 pandemic was published and recommended that GC users increase their daily GC dose during COVID-19 infection and to take GC every 6–12 hours (depending on the type of GC used) (30). GC users who need to be admitted to hospital because of COVID-19 should receive GC intravenously (30).

CONCLUSION

Patients receiving GC treatment prior to COVID-19 infection face excess mortality in the aftermath of their acute COVID-19 infection, particularly from sepsis, pulmonary embolism, and COVID-19. These patients also have a high risk of intensive care unit

For peer review only - http://bmjopen.bmj.com/site/about/guidelines.xhtml

admission and death due to COVID-19, and close monitoring and clinical attention may thus prevent their death.

Acknowledgements: We thank Peter Todd of Tajut Ltd. (Kaiapoi, New Zealand) for third-party editorial support, for which he received financial compensation from ALF funding.

Authors contributions

OR, ME, BK, FN, HL, DSO, and GJ were involved in the conceptualisation and design of the study. FN and HL acquired the data. BK and HL performed statistical analysis. OR, ME, BK, FN, HL, DSO, and GJ were involved with interpretation of the data and initial drafting of the manuscript. All authors participated in the critical appraisal, further interpretation, revision, and approved the final manuscript.

Competing interests statement

GJ has served as consultant for Ascendis Pharma, Astra Zeneca, and Novo Nordisk, and has received lecture fees from Novo Nordisk and Pfizer. DSO has received unrestricted project grants from Pfizer and is an employee at AstraZeneca as of August 8, 2021. FN holds some AstraZeneca shares. BK, ME, HL, and OR have nothing to disclose.

Funding information

ALF agreement (ALFGBG-938453, ALFGBG-971130, ALFGBG-978954, ALFGBG-966066), Swedish Research Council (2020-02828, 2019-01112), and Swedish Heart-Lung Foundation (20210030, 20210581).

Role of the funding sources

The funders of the study had no role in study design, data collection, data analysis, data interpretation, or writing of this manuscript.

For peer review only

Data availability statement

The individual level data used in this study are pseudonymised and sourced from Swedish healthcare registers. Interested researchers can obtain access to the data from the appropriate Swedish public data holders subject to obtaining ethics approval for the research and adhering to all relevant legislation, processes, and data protection protocols.

For peer review only

References

1. Einarsdottir MJ, Ekman P, Trimpou P, et al. High prescription rate of oral glucocorticoids in children and adults: A retrospective cohort study from Western Sweden. *Clin Endocrinol (Oxf)* 2020;92(1):21-28.
2. Einarsdottir MJ, Ekman P, Molin M, et al. High mortality rate in oral glucocorticoid users: a population-based matched cohort study. *Front Endocrinol (Lausanne)* 2022;13:918356.
3. Manetti L, Bogazzi F, Giovannetti C, et al. Changes in coagulation indexes and occurrence of venous thromboembolism in patients with Cushing's syndrome: results from a prospective study before and after surgery. *Eur J Endocrinol* 2010;163(5):783-91.
4. Broersen LH, Pereira AM, Jorgensen JO, et al. Adrenal insufficiency in corticosteroids use: Systematic review and meta-analysis. *J Clin Endocrinol Metab* 2015;100(6):2171-80.
5. Helms J, Tacquard C, Severac F, et al. High risk of thrombosis in patients with severe SARS-CoV-2 infection: a multicenter prospective cohort study. *Intensive Care Med* 2020:1-10.
6. Katsoularis I, Fonseca-Rodríguez O, Farrington P, et al. Risk of acute myocardial infarction and ischaemic stroke following COVID-19 in Sweden: a self-controlled case series and matched cohort study. *Lancet* 2021;398(10300):599-607.
7. Sarfraz A, Sarfraz Z, Razzack AA, et al. Venous Thromboembolism, Corticosteroids and COVID-19: A Systematic Review and Meta-Analysis. *Clin Appl Thromb Hemost* 2021;27:1076029621993573.
8. World Health Organization. Clinical management of COVID-19: interim guidance, 27 May 2020 2020 [Available from: https://apps.who.int/iris/handle/10665/332196 accessed June 14 2023.
9. Li H, Chen C, Hu F, et al. Impact of corticosteroid therapy on outcomes of persons with SARS-CoV-2, SARS-CoV, or MERS-CoV infection: a systematic review and meta-analysis. *Leukemia* 2020;34(6):1503-11.
10. RECOVERY Collaborative Group; Horby P, Lim WS, Emberson JR, et al. Dexamethasone in hospitalized patients with Covid-19. N Engl J Med 2021; 25;384: 693–704.
11. World Health Organization. Corticosteroids for COVID-19, Living guidance 2 September 2020 2020 [Available from: https://www.who.int/publications/i/item/WHO-2019-nCoV-Corticosteroids-2020.1 accessed 8 October 2020.
12. Patel C, Parmar K, Patel D, et al. Effect of corticosteroid therapy on mortality in COVID-19 patients-A systematic review and meta-analysis. *Rev Med Virol* 2022;32(5):e2386.
13. Higher dose corticosteroids in patients admitted to hospital with COVID-19 who are hypoxic but not requiring ventilatory support (RECOVERY): a randomised, controlled, open-label, platform trial. *Lancet* 2023;401(10387):1499-507.
14. Gianfrancesco M, Hyrich KL, Al-Adely S, et al. Characteristics associated with hospitalisation for COVID-19 in people with rheumatic disease: data from the COVID-19 Global Rheumatology Alliance physician-reported registry. *Ann Rheum Dis* 2020;79(7):859-66.
15. Calderón-Parra J, Cuervas-Mons V, Moreno-Torres V, et al. Influence of chronic use of corticosteroids and calcineurin inhibitors on COVID-19 clinical outcomes: analysis of a nationwide registry. *Int J Infect Dis* 2022;116:51-58.
16. Nyberg F, Franzén S, Lindh M, et al. Swedish Covid-19 Investigation for Future Insights - A Population Epidemiology Approach Using Register Linkage (SCIFI-PEARL). *Clin Epidemiol* 2021;13:649-59.

For peer review only

17. Ludvigsson JF, Svedberg P, Olén O, et al. The longitudinal integrated database for health insurance and labour market studies (LISA) and its use in medical research. *Eur J Epidemiol* 2019;34(4):423-37.
18. The Swedish Prescribed Drug Register. Socialstyrelsen. [cited 2023 September 11]. Available from: https://www.socialstyrelsen.se/statistik-och-data/register/lakemedelsregistret/ [
19. The National Patient Register. Socialstyrelsen. [cited 2023 September 8]. Available from: https://www.socialstyrelsen.se/statistik-och-data/register/patientregistret/ [
20. Stuart EA, King G, Imai K, et al. MatchIt: nonparametric preprocessing for parametric causal inference. *Journal of statistical software* 2011
21. Stuck AE, Minder CE, Frey FJ. Risk of infectious complications in patients taking glucocorticosteroids. *Rev Infect Dis* 1989;11(6):954-63.
22. Ragnarsson O, Olsson DS, Papakokkinou E, et al. Overall and Disease-Specific Mortality in Patients With Cushing Disease: A Swedish Nationwide Study. *J Clin Endocrinol Metab* 2019;104(6):2375-84.
23. Papakokkinou E, Olsson DS, Chantzichristos D, et al. Excess morbidity persists in patients with Cushing's disease during long-term remission - a Swedish nationwide study. *J Clin Endocrinol Metab* 2020
24. Johannesdottir SA, Horvath-Puho E, Dekkers OM, et al. Use of glucocorticoids and risk of venous thromboembolism: a nationwide population-based case-control study. *JAMA Intern Med* 2013;173(9):743-52.
25. Souverein PC, Berard A, Van Staa TP, et al. Use of oral glucocorticoids and risk of cardiovascular and cerebrovascular disease in a population based case-control study. *Heart* 2004;90(8):859-65.
26. Austin PC. Balance diagnostics for comparing the distribution of baseline covariates between treatment groups in propensity-score matched samples. *Statistics in medicine* 2009;28(25):3083-107.
27. Austin PC. The use of propensity score methods with survival or time-to-event outcomes: reporting measures of effect similar to those used in randomized experiments. *Statistics in medicine* 2014;33(7):1242-58.
28. Labor M, Kirui BK, Nyberg F, et al. Regular Inhaled Corticosteroids Use May Protect Against Severe COVID-19 Outcome in COPD. *Int J Chron Obstruct Pulmon Dis* 2023;18:1701-12.
29. Schlaghecke R, Kornely E, Santen RT, et al. The effect of long-term glucocorticoid therapy on pituitary-adrenal responses to exogenous corticotropin-releasing hormone. *N Engl J Med* 1992;326(4):226-30.
30. Arlt W, Baldeweg SE, Pearce SHS, et al. Endocrinology in the time of COVID-19: Management of adrenal insufficiency. *Eur J Endocrinol* 2020;183(1):G25-g32.

For peer review only

Table 1: Baseline characteristics of individuals with COVID-19 infection in Sweden from January 2020 to November 2021 without and with glucocorticoid exposure prior to the infection

	Non-exposed (n=1 151 347)	Any prior GC exposure* (n=48 806)		High GC exposure† (n=13 497)	
			SMD *vs* non-exposed		SMD *vs* non-exposed
Age (years)	37·9 (19·2)	51·4 (21·2)	0·666	62·4 (19·7)	1·257
Age category (years)			0·672		1·276
0–9	56 461 (4·9%)	930 (1·9%)		91 (0·7%)	
10–19	171 590 (14·9%)	3056 (6·3%)		284 (2·1%)	
20–29	200 477 (17·4%)	4491 (9·2%)		567 (4·2%)	
30–39	200 414 (17·4%)	5882 (12·1%)		925 (6·9%)	
40–49	205 427 (17·8%)	8045 (16·5%)		1514 (11·2%)	
50–59	167 108 (14·5%)	8993 (18·4%)		2249 (16·7%)	
60–69	79 704 (6·9%)	6396 (13·1%)		188 (16·2%)	
70–79	37 797 (3·3%)	5725 (11·7%)		2680 (19·9%)	
80–89	23 055 (2·0%)	4123 (8·4%)		2302 (17·1%)	
≥90	9314 (0·8%)	1165 (2·4%)		697 (5·2%)	
Sex			0·149		0·147
Male	566 579 (49·2%)	20 407 (41·8%)		5659 (41·9%)	
Female	584 768 (50·8%)	28 399 (58·2%)		7838 (58·1%)	
Birth country			0·105		0·199
Sweden	883 118 (76·7%)	38 515 (78·9%)		10 923 (80·9%)	
Nordic	18 577 (1·6%)	1285 (2·6%)		484 (3·6%)	
Europe	83 455 (7·2%)	3142 (6·4%)		779 (5·8%)	
Other	166 197 (14·4%)	5864 (12·0%)		1311 (9·7%)	
Employment status			0·453		0·888

For peer review only

Employed	838 341 (72·8%)	31 232 (64·0%)		6432 (47·7%)	
Unemployed	172 951 (15·0%)	15 328 (31·4%)		6857 (50·8%)	
Unknown	140 055 (12·2%)	2246 (4·6%)		208 (1·5%)	
Education level			0·300		0·501
Primary	173 194 (15·0%)	9817 (20·1%)		3672 (27·2%)	
Secondary	426 657 (37·1%)	19 950 (40·9%)		5656 (41·9%)	
Higher	376 185 (32·7%)	15 855 (32·5%)		3714 (27·5%)	
Unknown	175 311 (15·2%)	3184 (6·5%)		455 (3·4%)	
Comorbidities					
Pulmonary embolism	7977 (0·7%)	1676 (3·4%)	0·194	853 (6·3%)	0·310
Stroke	12 140 (1·1%)	1679 (3·4%)	0·161	852 (6·3%)	0·282
Deep vein thrombosis	1085 (0·1%)	301 (0·6%)	0·088	97 (0·7%)	0·098
Hypertension	86 847 (7·5%)	13 486 (27·6%)	0·547	6255 (46·3%)	0·972
Ischaemic heart disease	26 541 (2·3%)	4370 (9·0%)	0·291	2147 (15·9%)	0·487
Overall CV disease	149 594 (13·0%)	19 033 (39·0%)	0·621	8293 (61·4%)	1·158
Heart failure	18 043 (1·6%)	4456 (9·1%)	0·341	2347 (17·4%)	0·561
Asthma	37 185 (3·2%)	7841 (16·1%)	0·445	2026 (15·0%)	0·418
COPD	8731 (0·8%)	3856 (7·9%)	0·356	1700 (12·6%)	0·488
Emphysema	793 (0·1%)	392 (0·8%)	0·112	199 (1·5%)	0·161
Diabetes mellitus	42 598 (3·7%)	5478 (11·2%)	0·289	2638 (19·5%)	0·510
Rheumatic disease	8727 (0·8%)	6568 (13·5%)	0·510	4109 (30·4%)	0·897
Cancer	44 717 (3·9%)	7666 (15·7%)	0·406	3511 (26·0%)	0·653
Prescribed medications					
Inhaled corticosteroids	120 572 (10·5%)	17 815 (36·5%)	0·645	4258 (31·5%)	0·536
Oral anticoagulants	57 018 (5·0%)	8693 (17·8%)	0·413	3992 (29·6%)	0·689
Statins	101 469 (8·8%)	11 096 (22·7%)	0·389	4556 (33·8%)	0·640
ACEIs	67 321 (5·8%)	6969 (14·3%)	0·283	2860 (21·2%)	0·460
Opioids	112 707 (9·8%)	12 505 (25·6%)	0·424	4523 (33·5%)	0·601

Data are n (%) or mean (SD). *One or more prescriptions of oral GCs within 12 months before the COVID-19 infection date. †Two or more prescriptions and prednisolone ≥750 mg or equivalent amount of betamethasone, dexamethasone, methylprednisolone, prednisone, hydrocortisone, cortisone, or deflazacort within 6 months before COVID-19 infection date. Note this group is a subset

of any prior exposure. ACEI, angiotensin-converting enzyme inhibitor; COPD, chronic obstructive pulmonary disease; CV, cardiovascular; GC, glucocorticoid; SMD, standardised mean difference.

For peer review only

Figure legends

Figure 1: **Unadjusted cumulative incidence of all-cause (a) and cause-specific (b–f) mortality among COVID-19 patients in Sweden with any prior exposure* to oral glucocorticoids compared to non-exposed, from COVID-19 infection date to Nov 30, 2021**

GC, glucocorticoids. *Any prior GC exposure equals ≥1 prescription of oral GC within 12 months before the COVID-19 infection date.

Figure 2: **Unadjusted cumulative incidence of all-cause (a) and cause-specific (b–f) mortality among COVID-19 patients with any prior exposure* to oral glucocorticoids compared to non-exposed, from COVID-19 infection date to Nov 30, 2021**

GC, glucocorticoids. *High GC exposure equals ≥2 prescriptions with a total of ≥750 mg of prednisolone or equivalent within 6 months before COVID-19 infection date. This group is a subset of any prior exposure see figure 1).

Figure 3: **Adjusted Cox regression analysis and propensity score matched analysis comparing the risk of all-cause and cause-specific mortality between COVID-19 patients with prior exposure to oral glucocorticoids and non-exposed patients**

CI, confidence interval; HR, hazard ratio; ICU, intensive care unit; PS, propensity score. Any prior GC exposure equals ≥1 prescription of oral GC within 12 months before the COVID-19 infection date. High GC exposure equals ≥2 prescriptions with a total of ≥750 mg of prednisolone or equivalent within 6 months before COVID-19

For peer review only

infection date. The latter group is a subset of any prior exposure. *Adjusted for covariates; †Propensity score matched on covariates.

For peer review only

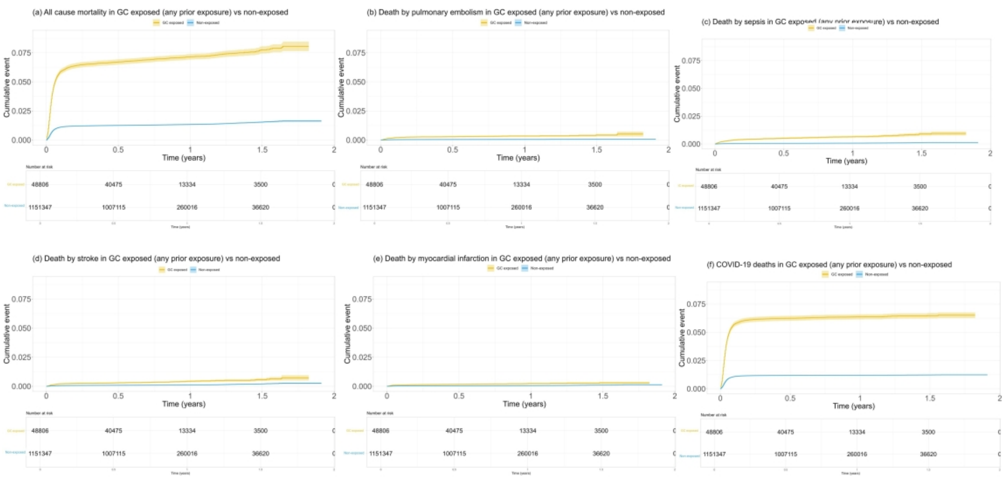

Figure 1: Unadjusted cumulative incidence of all-cause (a) and cause-specific (b–f) mortality among COVID-19 patients in Sweden with any prior exposure* to oral glucocorticoids compared to non-exposed, from COVID-19 infection date to Nov 30, 2021
GC, glucocorticoids. *Any prior GC exposure equals ≥1 prescription of oral GC within 12 months before the COVID-19 infection date.

338x190mm (300 x 300 DPI)

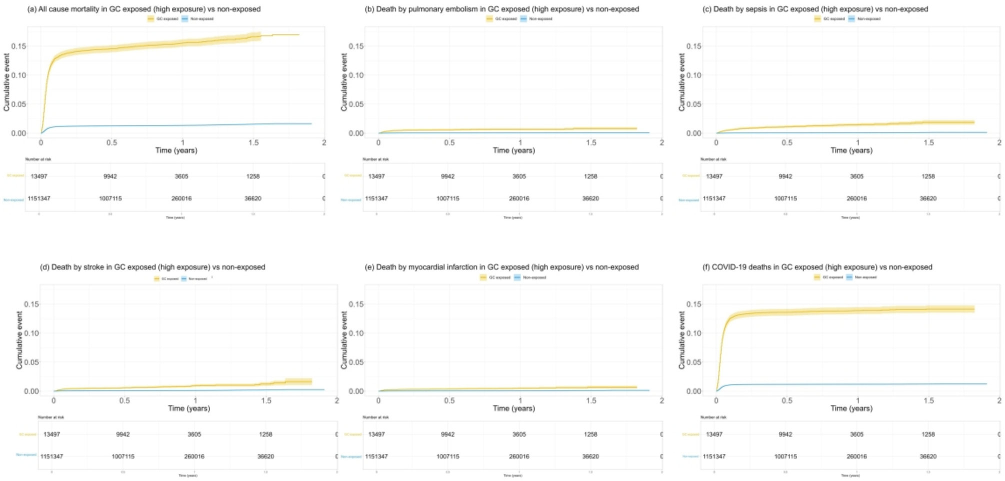

Figure 2: Unadjusted cumulative incidence of all-cause (a) and cause-specific (b–f) mortality among COVID-19 patients with any prior exposure* to oral glucocorticoids compared to non-exposed, from COVID-19 infection date to Nov 30, 2021
GC, glucocorticoids. *High GC exposure equals ≥2 prescriptions with a total of ≥750 mg of prednisolone or equivalent within 6 months before COVID-19 infection date. This group is a subset of any prior exposure see figure 1).

338x190mm (300 x 300 DPI)

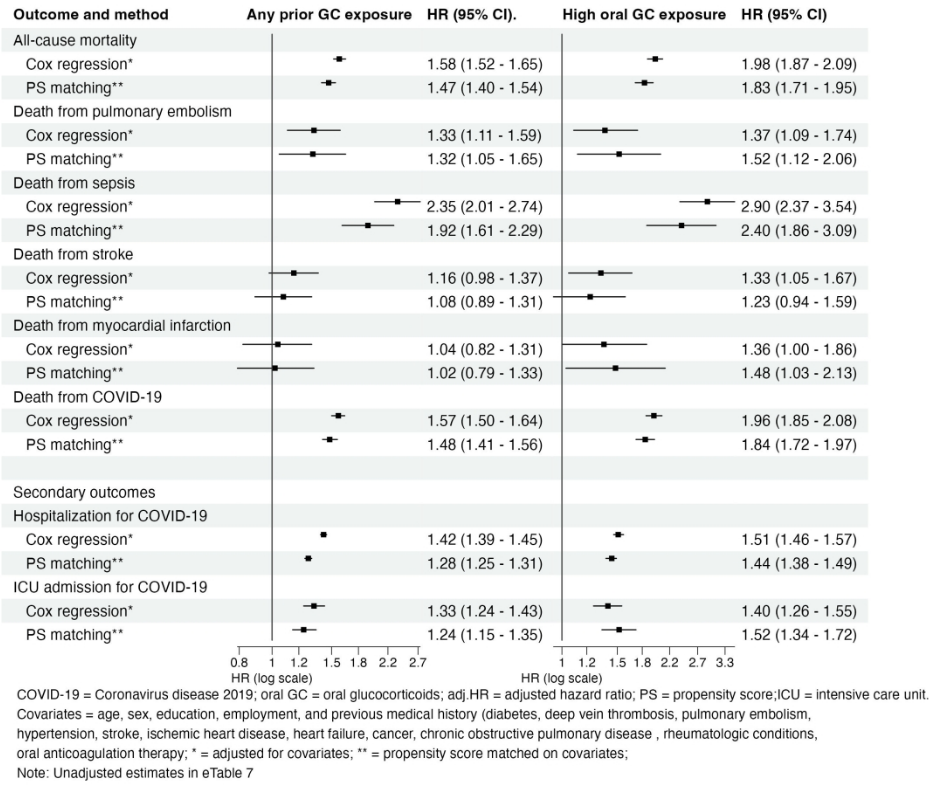

Figure 3: Adjusted Cox regression analysis and propensity score matched analysis comparing the risk of all-cause and cause-specific mortality between COVID-19 patients with prior exposure to oral glucocorticoids and non-exposed patients
CI, confidence interval; HR, hazard ratio; ICU, intensive care unit; PS, propensity score. Any prior GC exposure equals ≥1 prescription of oral GC within 12 months before the COVID-19 infection date. High GC exposure equals ≥2 prescriptions with a total of ≥750 mg of prednisolone or equivalent within 6 months before COVID-19 infection date. The latter group is a subset of any prior exposure. *Adjusted for covariates; †Propensity score matched on covariates.

971x932mm (72 x 72 DPI)

Supplementary appendix

Supplementary Table S1: **Dose equivalents used in the calculation of equivalence to prednisolone............ 2**

Supplementary Table S2: **Covariates selected in the evaluation of the effect of glucocorticoid treatment on death in COVID-19 patients in the Swedish population-based cohort of COVID-19 patients from COVID-19 infection date to Nov 30, 2021.. 3**

Supplementary Table S3: **Outcome events in individuals with COVID-19 infection in the Swedish population during the period 2015 to COVID-19 infection date until Nov 30, 2021 stratified by level of exposure to oral glucocorticoids (non-exposed, high exposure in the 6 months before infection, or any exposure 12 months before infection and overall).. 5**

Supplementary Table S4: **Baseline characteristics of individuals with COVID-19 infection in the Swedish population during the period 2015 to COVID-19 infection date (comorbidities) or 1 year before COVID-19 infection date (medications) until Nov 30, 2021, stratified by level of exposure to oral glucocorticoids (high exposure in the 6 months before infection or any exposure 12 months before infection), with standardized mean differences between exposed and 1:2 propensity score matched non-exposed 6**

Supplementary Table S5: **Outcome events in propensity score matched cohorts of individuals with COVID-19 infection in the Swedish population during the period 2015 to COVID-19 infection date until Nov 30, 2021 stratified by level of exposure to oral glucocorticoids (non-exposed, high exposure in the 6 months before infection, or any exposure 12 months before infection and overall).................................... 8**

Supplementary Table S6: **Standardised mean differences between the 15 a priori selected covariates of individuals with COVID-19 infections in the Swedish population during the period 2015 to COVID-19 infection date (comorbidities) or 1 year before COVID-19 infection date (medications) in those exposed to oral glucocorticoids compared with those non-exposed before and after propensity score matching, main analysis.. 9**

Supplementary Table S7: **Standardised mean differences between the 15 a priori selected plus added‡ covariates in sensitivity analysis of individuals with COVID-19 infections in the Swedish population during the period 2015 to COVID-19 infection date (comorbidities) or 1 year before COVID-19 infection date (medications) in those exposed to oral glucocorticoids compared with those non-exposed before and after propensity score matching... 10**

Supplementary Table S8: **Unadjusted, as well as adjusted from main and sensitivity analysis of the risk of various causes of death and all-cause mortality between COVID-19 patients who had any or high prior exposure to oral glucocorticoids and non-exposed individuals, hazard ratios from Cox regression analyses and propensity score matched analysis with 95% confidence intervals.. 11**

Supplementary Figure S1: **Propensity score balance before (a, c) and after (b, d) performing propensity score matching on all 15 covariates for individuals with COVID-19 infections in the Swedish population between 2015 and the COVID-19 infection date comparing those exposed to oral glucocorticoids (pink) with those who were non-exposed (blue) in those with any (a, b) or high glucocorticoid exposure (c, d).. 13**

Supplementary Figure S2: Standardised mean differences between the covariates of individuals with COVID-19 infections in the Swedish population during the period 2015 to COVID-19 infection date (comorbidities) or 1 year before COVID-19 infection date (medications) in the exposed to oral glucocorticoids compared with non-exposed before (red circles) and after (blue triangles) propensity score matching among those with (a) any or (b) high glucocorticoid exposure. 14

For peer review only

Supplementary Table S1: **Dose equivalents used in the calculation of equivalence to prednisolone**

ATC code	Recommended international non-proprietary drug name	Equivalent dose to 1 mg of prednisolone (mg)	Equivalent dose to 750 mg of prednisolone (mg)*
H02AB01	Betamethasone	8·333	6250·0
H02AB02	Dexamethasone	6·666	5000·0
H02AB04	Methylprednisolone	1·25	937·5
H02AB06	Prednisolone	1	750·0
H02AB07	Prednisone	1	750·0
H02AB09	Hydrocortisone	0·25	187·5
H02AB10	Cortisone	0·2	150·0
H02AB13	Deflazacort	0·666	500·0

ATC, Anatomical Therapeutic Chemical. *The definition of high oral glucocorticoid exposure was ≥2 prescriptions of prednisolone ≥750 mg or equivalent within 6 months before COVID-19 infection date. The corresponding equivalent amount to prednisolone 750 mg or the other oral glucocorticoids is shown on this column.

For peer review only

Supplementary Table S2: **Covariates selected in the evaluation of the effect of glucocorticoid treatment on death in COVID-19 patients in the Swedish population-based cohort of COVID-19 patients from COVID-19 infection date to Nov 30, 2021**

Covariate	Units and categories	Register source; description	Lookback window before COVID-19 infection date (index date)
Sociodemographic characteristics			
Age	As a continuous variable and in 10-year categories	RTB	At Jan 1, 2020
Sex	Male, female	RTB	At Jan 1, 2020
Education	Primary, secondary, higher, or unknown	LISA: primary education included pre-secondary education <9, 9, or >9 years. Secondary educated included 2-year high school education, secondary education <3 years or >3 years, and higher education included those with post-high school education. Unknown includes children with no education information or individuals with no relevant education (eg, immigrants).	At Jan 1, 2020
Employment status	Employed, unemployed, or unknown	LISA: created using the variable "employment" and stratified by age where: "employed" was either employed or with entrepreneurial income, and "unemployed" was the unemployed and without entrepreneurial income and includes pensioners. Unknown includes individuals with no information on employment.	At Jan 1, 2020
Comorbidity	**Units/categories**	**NPR: ICD-10 codes, any mention of condition as primary or secondary diagnosis for a hospitalisation or ≥1 visit at any time**	Jan 1, 2015 to index date
Pulmonary embolism	Yes/No	NPR: I26	Jan 1, 2015 to index date
Stroke	Yes/No	NPR: I61 I63 I649 I691 I693 I694 I698	Jan 1, 2015 to index date
Deep vein thrombosis	Yes/No	NPR: I80–I82	Jan 1, 2015 to index date
Hypertension	Yes/No	NPR: I10–I15	Jan 1, 2015 to index date
Ischaemic heart disease	Yes/No	NPR: I20 –I25	Jan 1, 2015 to index date
Overall cardiovascular disease	Yes/No	NPR: I00–I99	Jan 1, 2015 to index date
Heart failure	Yes/No	NPR: I50	Jan 1, 2015 to index date
Asthma	Yes/No	NPR: J45	Jan 1, 2015 to index date
COPD	Yes/No	NPR: J44	Jan 1, 2015 to index date
Emphysema	Yes/No	NPR: J43	Jan 1, 2015 to index date
Diabetes	Yes/No	NPR: E10, E11, E13, E14	Jan 1, 2015 to index date
Rheumatic disease	Yes/No	NPR: M05, M06, M31, M35	Jan 1, 2015 to index date
Cancer	Yes/No	NPR: C00–C97	Jan 1, 2015 to index date
Covariates for sensitivity analysis*			
Adrenal insufficiency	Yes/No	NPR: E271, E272, E230, E230A, E230B, E230C, E230D, E230E, E230F, E230W, E230X, E893, E310	Jan 1, 2015 to index date
Rheumatoid arthritis	Yes/No	NPR: M050-M053, M058, M058A, M058B, M058C, M058D, M058F, M058G, M058H, M058L, M058M, M058N, M058X, M059, M059L, M059M, M059N, M06-P, M060, M060L, M060M, M060N, M061-M064, M068, M068L, M068M, M068N, M069, M069A, M069B, M069C, M069D, M069F, M069G, M069H, M069X	Jan 1, 2015 to index date
Chronic lower respiratory diseases	Yes/No	NPR: J41-J47	Jan 1, 2015 to index date
Other respiratory diseases principally affecting the interstitium	Yes/No	NPR: J80-J84	Jan 1, 2015 to index date
Non-infective enteritis and colitis,	Yes/No	NPR: K50-K52	Jan 1, 2015 to index date

Dermatitis and eczema	Yes/No	NPR:L20-L30	Jan 1, 2015 to index date
Inflammatory polyarthropathies	Yes/No	NPR: M05-M14	Jan 1, 2015 to index date
Systemic connective tissue disorders (except RA M31 and M35)	Yes/No	NPR: M30-M36 (excluding M35.3, M31.5 and M31.6)	Jan 1, 2015 to index date
Glomerular diseases,	Yes/No	NPR: N01-N08	Jan 1, 2015 to index date
Organ or tissue transplant	Yes/No	NPR: Z940-Z944, Z948	Jan 1, 2015 to index date
Chronic liver disease	Yes/No	NPR: K72-K75	Jan 1, 2015 to index date
Alcoholism and other substance abuse	Yes/No	NPR: F10-F19	Jan 1, 2015 to index date
Prescribed medications	**Units/categories**	**NPDR: ATC codes. At least one filled prescription in lookback window**	
Inhaled corticosteroids*	Yes/No	R03BA, R03AK, R03AL08, R03AL09	1 year before index
Oral anticoagulants	Yes/No	B01AA, B01AF	1 year before index
Statins*	Yes/No	C10AA	1 year before index
ACEIs*	Yes/No	C09A	1 year before index
Opioids*	Yes/No	N02AB	1 year before index

ACEIs, angiotensin-converting enzyme inhibitors; ATC, Anatomical Therapeutic Chemical; BMI, body mass index; COPD, chronic obstructive pulmonary disease; ICD-10, International Classification of Diseases, version 10; LISA, Longitudinal Integrated Database for Health Insurance and Labor Market Studies; NPDR, Swedish National Prescribed Drug Register; NPR, Swedish National Patient Register; RTB, Register of Total Population; SNAR, Swedish National Airways Register; *16 covariates for sensitivity analyses.

For peer review only

Supplementary Table S3: **Outcome events in individuals with COVID-19 infection in the Swedish population during the period 2015 to COVID-19 infection date until Nov 30, 2021 stratified by level of exposure to oral glucocorticoids (non-exposed, high exposure in the 6 months before infection, or any exposure 12 months before infection and overall)**

	No=0/Yes=1	Non-exposed (n=1 151 347)	Any GC exposure* (n=48 806)	High GC exposure† (n=13 497)	Total study population (n=1 200 153)
All-cause mortality					
	0	1 136 497 (98·7%)	45 428 (93·1%)	11 474 (85·0%)	1 181 925 (98·5%)
	1	14 850 (1·3%)	3378 (6·9%)	2023 (15·0%)	18 228 (1·5%)
Death from pulmonary embolism					
	0	1 150 769 (99·9%)	48 654 (99·7%)	13 417 (99·4%)	1 199 423 (99·9%)
	1	578 (0·1%)	152 (0·3%)	80 (0·6%)	730 (0·1%)
Death from sepsis					
	0	1 150 576 (99·9%)	48 520 (99·4%)	13 335 (98·8%)	1 199 096 (99·9%)
	1	771 (0·1%)	286 (0·6%)	162 (1·2%)	1057 (0·1%)
Death from myocardial infarction					
	0	1 150 755 (99·9%)	48 707 (99·8%)	13 440 (99·6%)	1 199 462 (99·9%)
	1	592 (0·1%)	99 (0·2%)	57 (0·4%)	691 (0·1%)
Death from stroke					
	0	1 150 152 (99·9%)	48 625 (99·6%)	13 396 (99·3%)	1 198 777 (99·9%)
	1	1195 (0·1%)	181 (0·4%)	101 (0·7%)	1376 (0·1%)
Death from COVID-19					
	0	1 137 876 (98·8%)	45 781 (93·8%)	11 678 (86·5%)	1 183 657 (98·6%)
	1	13 471 (1·2%)	3025 (6·2%)	1819 (13·5%)	16 496 (1·4%)
Hospitalisation for COVID-19					
	0	1 088 788 (94·6%)	38 383 (78·6%)	8460 (62·7%)	1 127 171 (93·9%)
	1	62 559 (5·4%)	10 423 (21·4%)	5037 (37·3%)	72 982 (6·1%)
ICU admission for COVID-19					
	0	1 144 510 (99·4%)	47 747 (97·8%)	13 004 (96·3%)	1 192 257 (99·3%)
	1	6837 (0·6%)	1059 (2·2%)	493 (3·7%)	7896 (0·7%)

GC, glucocorticoid; ICU, intensive care unit. *One or more prescriptions of oral GCs within 12 months before the COVID-19 infection date. †Two or more prescriptions and prednisolone ≥750 mg or equivalent within 6 months before COVID-19 infection date. Note this group is a subset of any prior exposure.

For peer review only

Supplementary Table S4: **Baseline characteristics of individuals with COVID-19 infection in the Swedish population during the period 2015 to COVID-19 infection date (comorbidities) or 1 year before COVID-19 infection date (medications) until Nov 30, 2021, stratified by level of exposure to oral glucocorticoids (high exposure in the 6 months before infection or any exposure 12 months before infection), with standardized mean differences between exposed and 1:2 propensity score matched non-exposed**

	Any prior GC exposure* (n=47 737)	Non-exposed matched to any prior exposure (n=91 878)	SMD (any prior *vs* matched non-exposed)	High GC exposure† (n=12 943)	Non-exposed matched to the high GC exposed (n=23 898)	SMD (high *vs* matched non-exposed)
Age (years)	50·83 (21·06)	49·95 (20·83)	0·042	61·60 (19·69)	60·17 (19·63)	0·073
Age category (years)			0·047			0·081
0–9	930 (1·9)	1840 (2·0)		91 (0·7)	174 (0·7)	
10–19	3056 (6·4)	6089 (6·6)		284 (2·2)	556 (2·3)	
20–29	4491 (9·4)	8982 (9·8)		567 (4·4)	1128 (4·7)	
30–39	5879 (12·3)	11 699 (12·7)		925 (7·1)	1834 (7·7)	
40–49	8041 (16·8)	15 882 (17·3)		1514 (11·7)	2940 (12·3)	
50–59	8953 (18·8)	17 620 (19·2)		2237 (17·3)	4481 (18·8)	
60–69	6221 (13·0)	11 784 (12·8)		2146 (16·6)	4095 (17·1)	
70–79	5321 (11·1)	9652 (10·5)		2515 (19·4)	4403 (18·4)	
80–89	3719 (7·8)	6374 (6·9)		2021 (15·6)	3243 (13·6)	
≥90	1126 (2·4)	1956 (2·1)		643 (5·0)	1044 (4·4)	
Sex			0·002			0·021
Male	20 132 (42·2)	38 844 (42·3)		5480 (42·3)	10 368 (43·4)	
Female	27 605 (57·8)	53 034 (57·7)		7463 (57·7)	13 530 (56·6)	
Birth country			0·087			0·137
Sweden	37 607 (78·8)	69 317 (75·4)		10 450 (80·7)	18 124 (75·8)	
Nordic	1223 (2·6)	2201 (2·4)		456 (3·5)	756 (3·2)	
Europe	3084 (6·5)	6953 (7·6)		744 (5·7)	1768 (7·4)	
Other	5823 (12·2)	13 407 (14·6)		1293 (10·0)	3250 (13·6)	
Employment status			0·036			0·053
Employed	31 069 (65·1)	61 146 (66·6)		6398 (49·4)	12 421 (52·0)	
Unemployed	14 422 (30·2)	26 260 (28·6)		6337 (49·0)	11 073 (46·3)	
Unknown	2246 (4·7)	4472 (4·9)		208 (1·6)	404 (1·7)	
Education level			0·018			0·018
Primary	9397 (19·7)	17 445 (19·0)		3408 (26·3)	6114 (25·6)	
Secondary	19 470 (40·8)	37 752 (41·1)		5466 (42·2)	10 160 (42·5)	
Higher	15 699 (32·9)	30 487 (33·2)		3628 (28·0)	6819 (28·5)	
Unknown	3171 (6·6)	6194 (6·7)		441 (3·4)	805 (3·4)	
Comorbidities						
Pulmonary embolism	1536 (3·2)	2643 (2·9)	0·020	768 (5·9)	1243 (5·2)	0·032
Stroke	1606 (3·4)	2780 (3·0)	0·019	793 (6·1)	1366 (5·7)	0·017
Deep vein thrombosis	278 (0·6)	542 (0·6)	0·001	87 (0·7)	154 (0·6)	0·003
Hypertension	12 570 (26·3)	23 230 (25·3)	0·024	5753 (44·4)	10 133 (42·4)	0·041
Ischaemic heart disease	4054 (8·5)	7209 (7·8)	0·024	1907 (14·7)	3201 (13·4)	0·039
Overall CV disease	18 019 (37·7)	31 289 (34·1)	0·077	7751 (59·9)	12 982 (54·3)	0·113
Heart failure	3989 (8·4)	7060 (7·7)	0·025	2033 (15·7)	3442 (14·4)	0·036
Asthma	7625 (16·0)	4507 (4·9)	0·368	1935 (15·0)	1591 (6·7)	0·270
COPD	3403 (7·1)	6444 (7·0)	0·004	1500 (11·6)	2769 (11·6)	0·001

For peer review only

Emphysema	354 (0·7)	404 (0·4)	0·039	185 (1·4)	190 (0·8)	0·061
Diabetes mellitus	5098 (10·7)	9671 (10·5)	0·005	2431 (18·8)	4336 (18·1)	0·016
Rheumatic disease	5523 (11·6)	7075 (7·7)	0·131	3557 (27·5)	4843 (20·3)	0·170
Cancer	7171 (15·0)	13 624 (14·8)	0·005	3219 (24·9)	6235 (26·1)	0·028
Prescribed medications						
Inhaled corticosteroids	17 363 (36·4)	14 069 (15·3)	0·496	4061 (31·4)	4602 (19·3)	0·281
Oral anticoagulants	8017 (16·8)	14 677 (16·0)	0·022	3617 (27·9)	6288 (26·3)	0·037
Statins	10 571 (22·1)	19 209 (20·9)	0·030	4240 (32·8)	7728 (32·3)	0·009
ACEIs	6601 (13·8)	12 781 (13·9)	0·002	2658 (20·5)	5130 (21·5)	0·023
Opioids	12 026 (25·2)	14 517 (15·8)	0·234	4284 (33·1)	5064 (21·2)	0·270

Data are n (%) or mean (SD). ACEI, angiotensin-converting enzyme inhibitor; COPD, chronic obstructive pulmonary disease; CV, cardiovascular; GC, glucocorticoid; SMD, standardised mean difference.
*One or more prescriptions of oral GCs within 12 months before the COVID-19 infection date. †Two or more prescriptions and prednisolone ≥750 mg or equivalent within 6 months before COVID-19 infection date. Note this group is a subset of any prior exposure.

For peer review only

Supplementary Table S5: **Outcome events in propensity score matched cohorts of individuals with COVID-19 infection in the Swedish population during the period 2015 to COVID-19 infection date until Nov 30, 2021 stratified by level of exposure to oral glucocorticoids (non-exposed, high exposure in the 6 months before infection, or any exposure 12 months before infection and overall)**

	No=0/Yes=1	Any GC exposure and matched non-exposed			High GC exposure and matched non-exposed		
		Any GC exposure* (n=47 737)	Matched non-exposed (n=91 878)	Total any exposure and matched non-exposed (n=139 615)	High GC exposure† (n=12 943)	Matched non-exposed (n=23 898)	Total high exposure and matched non-exposed (n=36 841)
All-cause mortality							
	0	44 573 (93·4%)	87 973 (95·7%)	132 546 (94·9%)	11 054 (85·4%)	22 024 (92·2%)	33 078 (89·8%)
	1	3164 (6·6%)	3905 (4·3%)	7069 (5·1%)	1889 (14·6%)	1874 (7·8%)	3763 (10·2%)
Death from pulmonary embolism							
	0	47 598 (99·7%)	91 691 (99·8%)	139 289 (99·8%)	12 867 (99·4%)	23 804 (99·6%)	36 671 (99·5%)
	1	139 (0·3%)	187 (0·2%)	326 (0·2%)	76 (0·6%)	94 (0·4%)	170 (0·5%)
Death from sepsis							
	0	47 467 (99·4%)	91 627 (99·7%)	139 094 (99·6%)	12 799 (98·9%)	23 786 (99·5%)	36 585 (99·3%)
	1	270 (0·6%)	251 (0·3%)	521 (0·4%)	144 (1·1%)	112 (0·5%)	256 (0·7%)
Death from myocardial infarction							
	0	47 646 (99·8%)	91 718 (99·8%)	139 364 (99·8%)	12 889 (99·6%)	23 830 (99·7%)	36 719 (99·7%)
	1	91 (0·2%)	160 (0·2%)	251 (0·2%)	54 (0·4%)	68 (0·3%)	122 (0·3%)
Death from stroke							
	0	475 63 (99·6%)	91 592 (99·7%)	139 155 (99·7%)	12 845 (99·2%)	23 752 (99·4%)	36 597 (99·3%)
	1	174 (0·4%)	286 (0·3%)	460 (0·3%)	98 (0·8%)	146 (0·6%)	244 (0·7%)
Death from COVID-19							
	0	44 904 (94·1%)	88 387 (96·2%)	133 291 (95·5%)	11 240 (86·8%)	22 219 (93·0%)	33459 (90·8%)
	1	2833 (5·9%)	3491 (3·8%)	6324 (4·5%)	1703 (13·2%)	1679 (7·0%)	3382 (9·2%)
Hospitalisation for COVID-19							
	0	37 981 (79·6%)	77 727 (84·6%)	115 708 (82·9%)	8252 (63·8%)	17 810 (74·5%)	26 062 (70·7%)
	1	9756 (20·4%)	14 151 (15·4%)	23 907 (17·1%)	4691 (36·2%)	6088 (25·5%)	10 779 (29·3%)
ICU admission for COVID-19							
	0	46 736 (97·9%)	90 383 (98·4%)	137 119 (98·2%)	12 470 (96·3%)	23 320 (97·6%)	35 790 (97·1%)
	1	1001 (2·1%)	1495 (1·6%)	2496 (1·8%)	473 (3·7%)	578 (2·4%)	1051 (2·9%)

GC, glucocorticoid; ICU, intensive care unit. *One or more prescriptions of oral GCs within 12 months before the COVID-19 infection date. †Two or more prescriptions and prednisolone ≥750 mg of or equivalent within 6 months before COVID-19 infection date. Note this group is a subset of any prior exposure.

Supplementary Table S6: **Standardised mean differences between the 15 a priori selected covariates of individuals with COVID-19 infections in the Swedish population during the period 2015 to COVID-19 infection date (comorbidities) or 1 year before COVID-19 infection date (medications) in those exposed to oral glucocorticoids compared with those non-exposed before and after propensity score matching, main analysis**

Variable	Any prior GC exposure*		High GC exposure†	
	SMD before PS matching	SMD after PS matching	SMD before PS matching	SMD after PS matching
Age categories	0·672	0·047	1·276	0·081
Sex	0·149	0·002	0·147	0·021
Education	0·300	0·018	0·501	0·018
Employment	0·453	0·036	0·888	0·053
Pulmonary embolism	0·194	0·020	0·310	0·032
Stroke	0·161	0·019	0·282	0·017
Deep vein thrombosis	0·088	0·001	0·098	0·003
Hypertension	0·547	0·024	0·972	0·041
Ischaemic heart disease	0·292	0·024	0·487	0·039
Heart failure	0·341	0·025	0·561	0·036
COPD	0·356	0·004	0·488	0·001
Diabetes mellitus	0·289	0·005	0·510	0·016
Rheumatic disease	0·510	0·131	0·897	0·170
Cancer	0·406	0·005	0·653	0·028
Oral anticoagulants	0·413	0·022	0·689	0·037

COPD, chronic obstructive pulmonary disease; GC, glucocorticoid; PS, propensity score; SMD, standardised mean difference. *One or more prescriptions of oral GCs within 12 months before the COVID-19 infection date. †Two or more prescriptions and a total of prednisolone ≥750 mg or equivalent within 6 months before COVID-19 infection date. Note this group is a subset of any prior exposure.

For peer review only

Supplementary Table S7: **Standardised mean differences between the 15 a priori selected plus added‡ covariates in sensitivity analysis of individuals with COVID-19 infections in the Swedish population during the period 2015 to COVID-19 infection date (comorbidities) or 1 year before COVID-19 infection date (medications) in those exposed to oral glucocorticoids compared with those non-exposed before and after propensity score matching**

Variable	Any prior GC exposure*		High GC exposure†	
	SMD before PS matching	SMD after PS matching	SMD before PS matching	SMD after PS matching
Age categories	0.672	0.052	1.276	0.046
Sex	0.149	0.006	0.147	0.019
Birth country	0.105	0.065	0.199	0.092
Employment status	0.453	0.048	0.888	0.035
Education level	0.300	0.023	0.501	0.017
Pulmonary embolism	0.194	0.034	0.310	0.026
Stroke	0.161	0.026	0.282	0.013
Deep vein thrombosis	0.088	0.005	0.098	0.006
Hypertension	0.547	0.053	0.972	0.045
Ischaemic heart disease	0.292	0.043	0.487	0.027
Heart failure	0.341	0.060	0.561	0.048
COPD	0.357	0.047	0.488	0.006
Diabetes mellitus	0·289	0.068	0·510	0.105
Rheumatic disease	0·510	0.039	0·897	0.021
Cancer	0·406	0.151	0·653	0.185
Oral anticoagulants	0.413	0.046	0.689	0.035
Added covariates‡				
Adrenal insufficiency	0.168	0.031	0.285	0.023
Rheumatoid arthritis	0.344	0.086	0.539	0.078
Chronic lower respiratory diseases	0.559	0.009	0.618	0.043
Other respiratory diseases principally affecting the interstitium	0.195	0.037	0.308	0.031
Non-infective enteritis and colitis,	0.255	0.020	0.322	0.056
Dermatitis and eczema	0.262	0.016	0.287	0.023
Inflammatory polyarthropathies	0.471	0.072	0.718	0.049
Systemic connective tissue disorders (except rheumatoid arthritis ICD-coded M31 and M35)	0.234	0.044	0.362	0.029
Glomerular diseases,	0.160	0.048	0.247	0.068
Chronic liver disease	0.140	0.070	0.213	0.009
Organ or tissue transplant	0.234	0.107	0.358	0.111
Alcoholism and other substance abuse	0.118	0.014	0.156	0.004
Opioids	0.424	0.016	0.601	0.008
Inhaled corticosteroids	0.645	0.059	0.535	0.071
Statins	0.389	0.027	0.640	0.014
ACEIs	0.283	0.016	0.460	0.001

COPD, chronic obstructive pulmonary disease; GC, glucocorticoid; PS, propensity score; SMD, standardised mean difference; ICD, 10th edition of the International Classification of Diseases; ACEIs, angiotensin converting enzyme inhibitors. *One or more prescriptions of oral GCs within 12 months before the COVID-19 infection date. †Two or more prescriptions and a total of prednisolone ≥750 mg or equivalent within 6 months before COVID-19 infection date. Note this group is a subset of any prior exposure. ‡ 16 other covariates of interest for oral GC users.

For peer review only

Supplementary Table S8: **Unadjusted, as well as adjusted from main and sensitivity analysis of the risk of various causes of death and all-cause mortality between COVID-19 patients who had any or high prior exposure to oral glucocorticoids and non-exposed individuals, hazard ratios from Cox regression analyses and propensity score matched analysis with 95% confidence intervals.**

**Outcomes and method**	**Any prior GC exposure***		**High GC exposure†**	
	**HR (95% CI)**	**No. of events**	**HR (95% CI)**	**No. of events**
All-cause mortality				
Unadjusted Cox regression	5·46 (5·26–5·67)	18 228	12·33 (11·77–12·92)	16 873
Adjusted Cox regression‡	1·58 (1·52–1·65)	18 228	1·98 (1·87–2·09)	16 873
Cox with extended adjustments‡‡ [Sensitivity analysis]	1.55 [1.48-1.61]	18 228	1.85 [1.75-1.96]	16 873
PS matching¶	1·47 (1·40–1·54)	7 069	1·83 (1·71–1·95)	3 763
PS matching with additional covariates¶¶[Sensitivity analysis]	1.53 [1.46-1.61]	6 673	1.67 [1.57-1.79]	3 886
Death from pulmonary embolism				
Unadjusted Cox regression	6·29 (5·26–7·52)	730	12·68 (10·04–16·03)	658
Adjusted Cox regression‡	1·33 (1·11–1·59)	730	1·37 (1·09–1·74)	658
Cox with extended adjustments‡‡ [Sensitivity analysis]	1.38 [1.16-1.65]	730	1.35 [1.07-1.71]	658
PS matching¶	1·32 (1·05–1·65)	326	1·52 (1·12–2·06)	170
PS matching with additional covariates¶¶[Sensitivity analysis]	1.54 [1.21-1.95]	290	1.52 [1.11-2.09]	160
Death from sepsis				
Unadjusted Cox regression	8·67 (7·57–9·94)	1 057	18·72 (15·79–22·19)	933
Adjusted Cox regression‡	2·35 (2·01–2·74)	1 057	2·90 (2·37–3·54)	933
Cox with extended adjustments‡‡ [Sensitivity analysis]	2.10 [1.79-2.47]	1 057	2.43 [1.96-3.00]	933
PS matching¶	1·92 (1·61–2·29)	521	2·40 (1·86–3·09)	256
PS matching with additional covariates¶¶[Sensitivity analysis]	1.87 [1.56-2.25]	492	1.90 [1.49-2.42]	276
Death from stroke				
Unadjusted Cox regression	3·47 (2·96–4·05)	1 376	7·22 (5·89–8·85)	1 296
Adjusted Cox regression‡	1·16 (0·98–1·37)	1 376	1·33 (1·05–1·67)	1 296
Cox with extended adjustments‡‡ [Sensitivity analysis]	1.14 [0.96-1.36]	1 376	1.23 [0.97-1.56]	1 296
PS matching¶	1·08 (0·89–1·31)	460	1·23 (0·94–1·59)	244
PS matching with additional covariates¶¶[Sensitivity analysis]	1.01 [0.83-1.24]	443	0.99 [0.76-1.28]	263
Death from myocardial infarction				
Unadjusted Cox regression	3·84 (3·10–4·75)	691	8·23 (6·27–10·82)	649
Adjusted Cox regression‡	1·04 (0·82–1·31)	691	1·36 (1·00–1·86)	649
Cox with extended adjustments‡‡ [Sensitivity analysis]	1.08 [0.85-1.38]	691	1.41 [1.02-1.94]	649
PS matching¶	1·02 (0·79–1·33)	251	1·48 (1·03–2·13)	122
PS matching with additional covariates¶¶[Sensitivity analysis]	1.05 [0.80-1.37]	239	1.24 [0.86-1.78]	125
Death from COVID–19				
Unadjusted Cox regression	5·43 (5·22–5·65)	16 496	12·34 (11·75–12·96)	15 290
Adjusted Cox regression‡	1·57 (1·50–1·64)	16 496	1·96 (1·85–2·08)	15 290
Cox with extended adjustments‡‡ [Sensitivity analysis]	1.53 [1.46-1.60]	16 496	1.83 [1.73-1.94]	15 290
PS matching¶	1·48 (1·41–1·56)	6 324	1·84 (1·72–1·97)	3 382
PS matching with additional covariates¶¶[Sensitivity analysis]	1.54 [1.46-1.62]	5 989	1.69 [1.58-1.81]	3 489
Secondary outcomes				
Hospitalisation for COVID–19				
Unadjusted Cox regression	4·34 (4·25–4·43)	72 982	8·52 (8·28–8·77)	67 596
Adjusted Cox regression‡	1·42 (1·39–1·45)	72 982	1·51 (1·46–1·57)	67 596
Cox with extended adjustments‡‡ [Sensitivity analysis]	1.20 [1.17-1.23]	72 982	1.25 [1.20-1.29]	67 596
PS matching¶	1·28 (1·25–1·31)	23 907	1·44 (1·38–1·49)	10 779

For peer review only

PS matching with additional covariates[¶¶][Sensitivity analysis]	1.19 [1.16-1.22]	23 814	1.29 [1.24-1.34]	11 037
ICU admission for COVID–19				
Unadjusted Cox regression	3·72 (3·49–3·97)	7 896	6·41 (5·85–7·03)	7 330
Adjusted Cox regression[‡]	1·33 (1·24–1·43)	7 896	1·40 (1·26–1·55)	7 330
Cox with extended adjustments[‡‡] [Sensitivity analysis]	0.90 [0.84-0.97]	7 896	0.80 [0.72-0.89]	7 330
PS matching[¶]	1·24 (1·15–1·35)	2 496	1·52 (1·34–1·72)	1 051
PS matching with additional covariates[¶¶][Sensitivity analysis]	0.99 [0.92-1.07]	2 703	1.00 [0.89-1.13]	1 206

CI, confidence interval; GC, glucocorticoid; HR, hazard ratio; PS, propensity score. *One or more prescriptions of oral GCs within 12 months before the COVID-19 infection date. [†]Two or more prescriptions and prednisolone ≥750 mg or equivalent within 6 months before COVID-19 infection date. Note high exposure is a subset of any prior exposure. [‡]Adjusted for initial a priori selected covariates: age, sex, education, employment, and previous medical history (diabetes, deep vein thrombosis, pulmonary embolism, hypertension, stroke, ischaemic heart disease, heart failure, cancer, chronic obstructive pulmonary disease, rheumatologic conditions, and oral anticoagulation therapy. [‡‡] Adjusted for additional covariates on top of initial (initial covariates + adrenal insufficiency, rheumatoid arthritis, chronic lower respiratory diseases, other respiratory diseases principally affecting the interstitium, non-infective enteritis and colitis, dermatitis and eczema, inflammatory polyarthropathies, systemic connective tissue disorders (except rheumatoid arthritis), glomerular diseases, organ or tissue transplant , chronic liver disease, medications: inhaled corticosteroids, statins, aceis, opioids, alcoholism and other substance abuse). [¶]1:2 matched on a PS score based on 15 initial a priori selected covariates: age, sex, education, employment, and previous medical history (diabetes, deep vein thrombosis, pulmonary embolism, hypertension, stroke, ischaemic heart disease, heart failure, cancer, chronic obstructive pulmonary disease, rheumatologic conditions, oral anticoagulation therapy). [¶¶] 1:2 matched on a PS score based on 31 covariates: initial 15 + adrenal insufficiency, rheumatoid arthritis, chronic lower respiratory diseases, other respiratory diseases principally affecting the interstitium, non-infective enteritis and colitis, dermatitis and eczema, inflammatory polyarthropathies, systemic connective tissue disorders (except rheumatoid arthritis), glomerular diseases, organ or tissue transplant , chronic liver disease, medications: inhaled corticosteroids, statins, aceis, opioids, alcoholism and other substance abuse.

For peer review only

Supplementary Figure S1: **Propensity score balance before (a, c) and after (b, d) performing propensity score matching on all 15 covariates for individuals with COVID-19 infections in the Swedish population between 2015 and the COVID-19 infection date comparing those exposed to oral glucocorticoids (pink) with those who were non-exposed (blue) in those with any (a, b) or high glucocorticoid exposure (c, d)**

GC, glucocorticoid; PS, propensity score. Any prior GC exposure equals ≥1 prescription of oral GC within 12 months before the COVID-19 infection date. High GC exposure equals ≥2 prescriptions with a total of ≥750 mg of prednisolone or equivalent. The latter group is a subset of any prior exposure. Note: The figure includes PS density plots and histograms illustrating the distribution of PS values. Prior to matching, the PS density plots and histograms represent the distribution of PS values for both exposed and non-exposed individuals. Subsequently, a 1:2 matching scheme was employed using a caliper of 0.2 where each exposed individual was paired with two non-exposed individuals based on their similarity in PS values. The PS density plots and histograms after matching reflect the distribution of PS values for the matched groups, demonstrating the achieved balance in the covariates. The 15 covariates used in the logistic model to estimate the propensity score can be found in Supplementary Table S7.

(a) Any GC exposure before PS matching

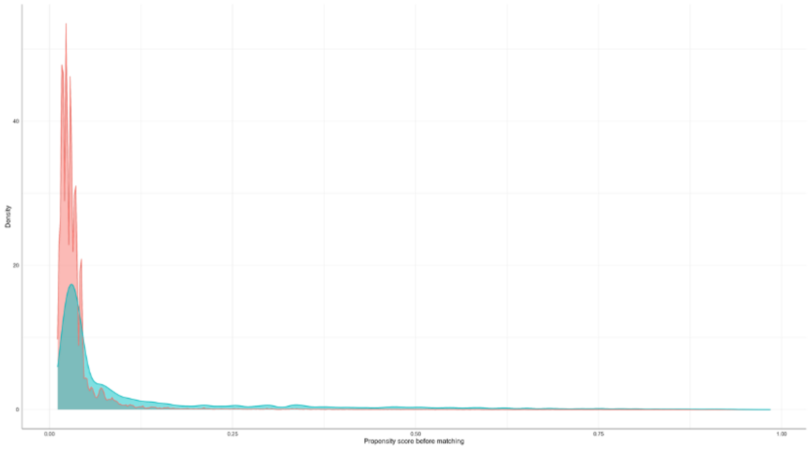

(b) Any GC exposure after PS matching

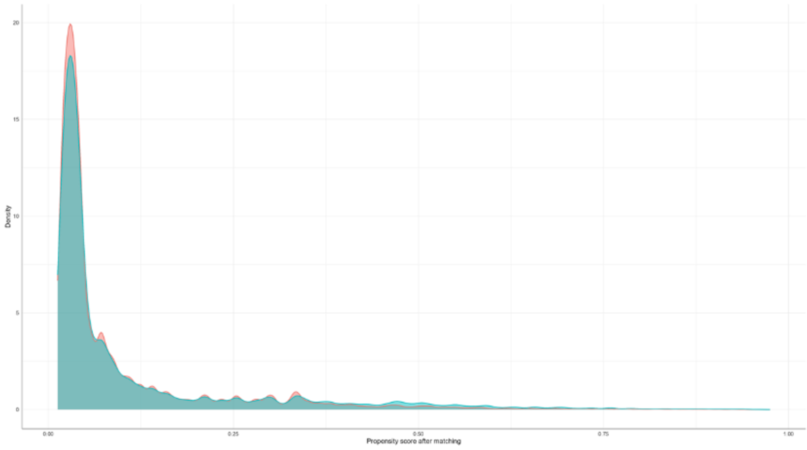

(c) High GC exposure before PS matching

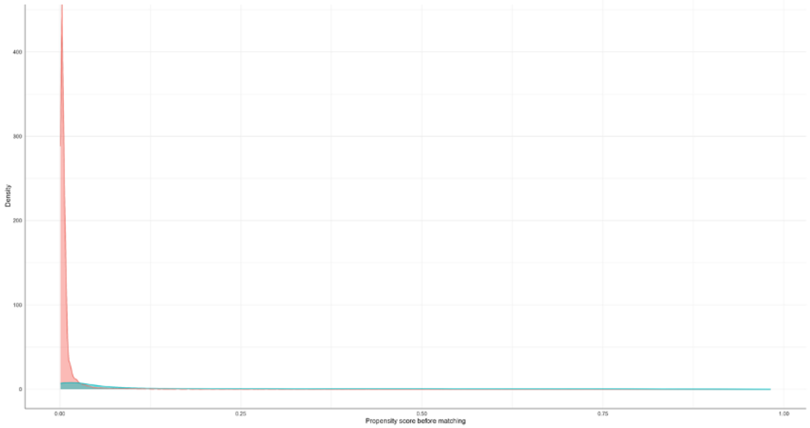

(d) High GC exposure after PS matching

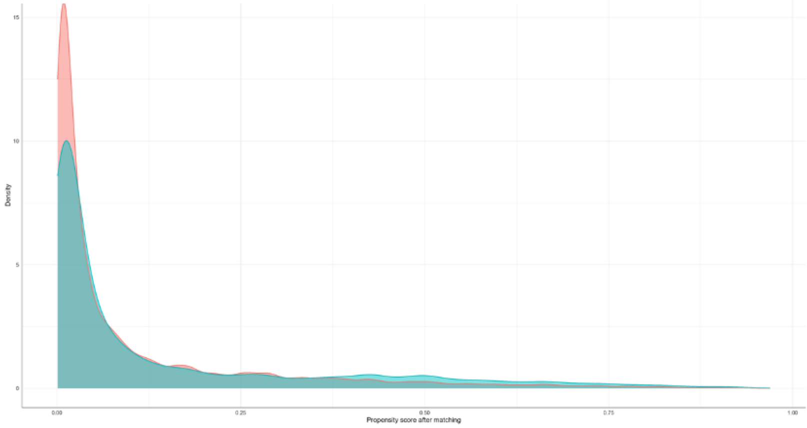

Supplementary Figure S2: Standardised mean differences between the covariates of individuals with COVID-19 infections in the Swedish population during the period 2015 to COVID-19 infection date (comorbidities) or 1 year before COVID-19 infection date (medications) in the exposed to oral glucocorticoids compared with non-exposed before (red circles) and after (blue triangles) propensity score matching among those with (a) any or (b) high glucocorticoid exposure. COPD, chronic obstructive pulmonary disease; GC, glucocorticoid. Any prior exposure equals ≥1 prescription of oral glucocorticoids within 12 months before the COVID-19 infection date. High exposure equals ≥2 prescriptions of oral glucocorticoids with a total of ≥750 mg of prednisolone or equivalent within 6 months before COVID-19 infection date. Note the latter group is a subset of any prior exposure.

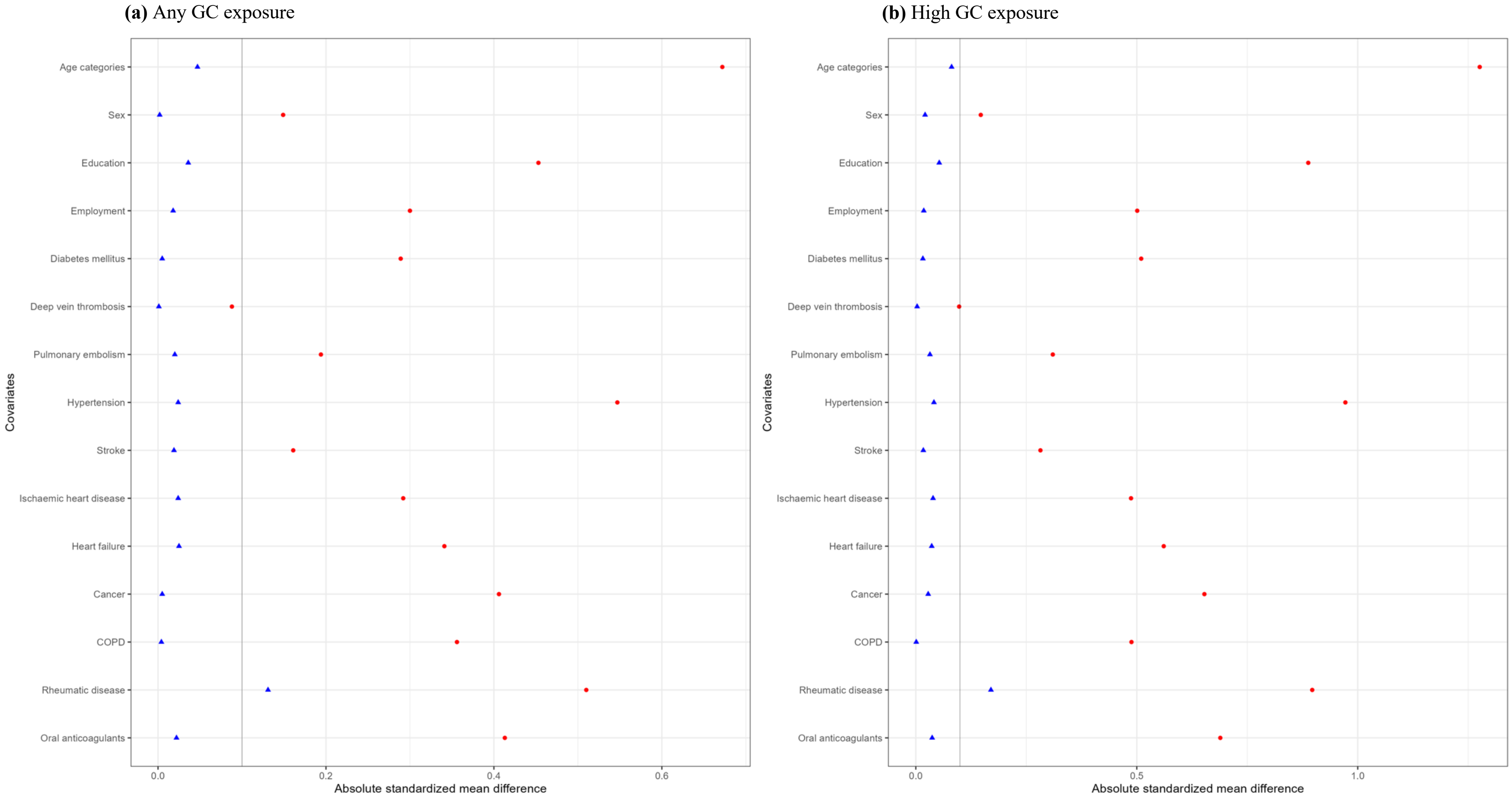

STROBE Statement—Checklist of items that should be included in reports of ***cohort studies***

	Item No	Recommendation
**Title and abstract**	1	(*a*) Indicate the study's design with a commonly used term in the title or the abstract **Page 1**
		(*b*) Provide in the abstract an informative and balanced summary of what was done and what was found **Page 5-6**
**Introduction**		
Background/rationale	2	Explain the scientific background and rationale for the investigation being reported **Page 7-9**
Objectives	3	State specific objectives, including any prespecified hypotheses **Page 9**
**Methods**		
Study design	4	Present key elements of study design early in the paper **Page 9-12**
Setting	5	Describe the setting, locations, and relevant dates, including periods of recruitment, exposure, follow-up, and data collection **Page 9-12**
Participants	6	(*a*) Give the eligibility criteria, and the sources and methods of selection of participants. Describe methods of follow-up **Page 9-12**
		(*b*) For matched studies, give matching criteria and number of exposed and unexposed **Page 12-15**
Variables	7	Clearly define all outcomes, exposures, predictors, potential confounders, and effect modifiers. Give diagnostic criteria, if applicable **Page 10-12**
Data sources/ measurement	8*	For each variable of interest, give sources of data and details of methods of assessment (measurement). Describe comparability of assessment methods if there is more than one group **Page 9-12**
Bias	9	Describe any efforts to address potential sources of bias **Page 12**
Study size	10	Explain how the study size was arrived at **Page 9**
Quantitative variables	11	Explain how quantitative variables were handled in the analyses. If applicable, describe which groupings were chosen and why **Page 12**
Statistical methods	12	(*a*) Describe all statistical methods, including those used to control for confounding **Page 12**
		(*b*) Describe any methods used to examine subgroups and interactions
		(*c*) Explain how missing data were addressed **No missing data reported**
		(*d*) If applicable, explain how loss to follow-up was addressed
		(*e*) Describe any sensitivity analyses **Page 14-15**
**Results**		
Participants	13*	(a) Report numbers of individuals at each stage of study—eg numbers potentially eligible, examined for eligibility, confirmed eligible, included in the study, completing follow-up, and analysed **Page 13**
		(b) Give reasons for non-participation at each stage
		(c) Consider use of a flow diagram
Descriptive data	14*	(a) Give characteristics of study participants (eg demographic, clinical, social) and information on exposures and potential confounders **Table 1**
		(b) Indicate number of participants with missing data for each variable of interest **No missing data reported**
		(c) Summarise follow-up time (eg, average and total amount) **Page 10-11**
Outcome data	15*	Report numbers of outcome events or summary measures over time **Supplementary table S5 and S6**
Main results	16	(*a*) Give unadjusted estimates and, if applicable, confounder-adjusted estimates and

For peer review only

For peer review only - http://bmjopen.bmj.com/site/about/guidelines.xhtml

		their precision (eg, 95% confidence interval). Make clear which confounders were adjusted for and why they were included **Supplementary table S6**
		(*b*) Report category boundaries when continuous variables were categorized **Supplementary Table S2**
		(*c*) If relevant, consider translating estimates of relative risk into absolute risk for a meaningful time period
Other analyses	17	Report other analyses done—eg analyses of subgroups and interactions, and sensitivity analyses **Page 14-15**
**Discussion**		
Key results	18	Summarise key results with reference to study objectives **Page 15**
Limitations	19	Discuss limitations of the study, taking into account sources of potential bias or imprecision. Discuss both direction and magnitude of any potential bias **Page 17-18**
Interpretation	20	Give a cautious overall interpretation of results considering objectives, limitations, multiplicity of analyses, results from similar studies, and other relevant evidence **Page 15-19**
Generalisability	21	Discuss the generalisability (external validity) of the study results **Page 18**
**Other information**		
Funding	22	Give the source of funding and the role of the funders for the present study and, if applicable, for the original study on which the present article is based **Page 5**

*Give information separately for exposed and unexposed groups.

Note: An Explanation and Elaboration article discusses each checklist item and gives methodological background and published examples of transparent reporting. The STROBE checklist is best used in conjunction with this article (freely available on the Web sites of PLoS Medicine at http://www.plosmedicine.org/, Annals of Internal Medicine at http://www.annals.org/, and Epidemiology at http://www.epidem.com/). Information on the STROBE Initiative is available at http://www.strobe-statement.org.

For peer review only

BMJ Open

Impact of chronic oral glucocorticoid treatment on mortality in patients with COVID-19: analysis of a population-based cohort

Journal:	*BMJ Open*
Manuscript ID	bmjopen-2023-080640.R2
Article Type:	Original research
Date Submitted by the Author:	04-Mar-2024
Complete List of Authors:	Einarsdottir, Margret; University of Gothenburg, Department of Internal Medicine and Clinical Nutrition; Sahlgrenska University Hospital, Department of Endocrinology<br>Kibiwott Kirui, Brian; University of Gothenburg, School of Public Health and Community Medicine, Institute of Medicine, Sahlgrenska Academy<br>Li, Huiqi; University of Gothenburg School of Public Health and Community Medicine<br>Olsson, Daniel; University of Gothenburg, Department of Internal Medicine and Clinical Nutrition; AstraZeneca<br>Johannsson, Gudmundur; University of Gothenburg, Department of Internal Medicine and Clinical Nutrition; Sahlgrenska University Hospital, Department of Endocrinology<br>Nyberg, Fredrik; University of Gothenburg School of Public Health and Community Medicine<br>Ragnarsson, Oskar; University of Gothenburg, Department of Internal Medicine and Clinical Nutrition; Sahlgrenska University Hospital, Department of Endocrinology
**Primary Subject Heading**:	Epidemiology
Secondary Subject Heading:	Epidemiology
Keywords:	Mortality, COVID-19, Adrenal disorders < DIABETES & ENDOCRINOLOGY

SCHOLARONE™
Manuscripts

I, the Submitting Author has the right to grant and does grant on behalf of all authors of the Work (as defined in the below author licence), an exclusive licence and/or a non-exclusive licence for contributions from authors who are: i) UK Crown employees; ii) where BMJ has agreed a CC-BY licence shall apply, and/or iii) in accordance with the terms applicable for US Federal Government officers or employees acting as part of their official duties; on a worldwide, perpetual, irrevocable, royalty-free basis to BMJ Publishing Group Ltd ("BMJ") its licensees and where the relevant Journal is co-owned by BMJ to the co-owners of the Journal, to publish the Work in this journal and any other BMJ products and to exploit all rights, as set out in our licence.

The Submitting Author accepts and understands that any supply made under these terms is made by BMJ to the Submitting Author unless you are acting as an employee on behalf of your employer or a postgraduate student of an affiliated institution which is paying any applicable article publishing charge ("APC") for Open Access articles. Where the Submitting Author wishes to make the Work available on an Open Access basis (and intends to pay the relevant APC), the terms of reuse of such Open Access shall be governed by a Creative Commons licence – details of these licences and which Creative Commons licence will apply to this Work are set out in our licence referred to above.

Other than as permitted in any relevant BMJ Author's Self Archiving Policies, I confirm this Work has not been accepted for publication elsewhere, is not being considered for publication elsewhere and does not duplicate material already published. I confirm all authors consent to publication of this Work and authorise the granting of this licence.

For peer review only

Impact of chronic oral glucocorticoid treatment on mortality in patients with COVID-19: analysis of a population-based cohort

Running title: Glucocorticoids, mortality and COVID-19

Authors: Margrét J Einarsdóttir (0000-0002-6010-9599)[1,2]*, Brian K Kirui (0000-0002-6656-6029)[3]*, Huiqi Li (0000-0002-1127-0829) [3], Daniel S Olsson (0000-0002-9734-0786)[1,2,5], Gudmundur Johannsson (0000-0003-3484-8440)[1,2], Fredrik Nyberg (0000-0003-0892-5668)[3†], Oskar Ragnarsson (0000-0003-0204-9492)[1,2,4†]

*Joint first authors

†Joint senior authors

Affiliations: [1]Department of Internal Medicine and Clinical Nutrition, Institute of Medicine, Sahlgrenska Academy, University of Gothenburg, Gothenburg, Sweden; [2]Department of Endocrinology, Sahlgrenska University Hospital, Gothenburg, Sweden; [3]School of Public Health and Community Medicine, Institute of Medicine, Sahlgrenska Academy, University of Gothenburg, Gothenburg, Sweden; [4]Wallenberg Center for Molecular and Translational Medicine, University of Gothenburg, Gothenburg, Sweden; [5]Cardiovascular, Renal and Metabolism (CVRM), BioPharmaceuticals R&D, AstraZeneca, Gothenburg, Sweden.

Address for correspondence and reprint requests: Oskar Ragnarsson, Department of Internal Medicine and Clinical Nutrition, Institute of Medicine,

For peer review only

Sahlgrenska Academy, University of Gothenburg, 405 30 Gothenburg, Sweden; Tel: +46 70 728 2229; E-mail: oskar.ragnarsson@medic.gu.se

Manuscript category: Original article

Abstract length: 247

Text length: 3376

No. of references: 32

No. of graphics: 4 (1 table and 3 figures)

Supplementary data: Appendix provided as separate file

Author addresses:

Margrét J Einarsdóttir, Department of Internal Medicine and Clinical Nutrition, Institute of Medicine, Sahlgrenska Academy, University of Gothenburg, 405 30 Gothenburg, Sweden; Tel: +46313427056; E-mail: margret.jona.einarsdottir@gu.se

Brian K Kirui, School of Public Health and Community Medicine, Institute of Medicine, Sahlgrenska Academy, University of Gothenburg, Gothenburg, Sweden; Tel: +46700579203; E-mail: brian.kirui@gu.se

Huiqi Li, School of Public Health and Community Medicine, Institute of Medicine, Sahlgrenska Academy, University of Gothenburg, Gothenburg, Sweden; Tel: +46317866251; E-mail: huiqi.li@gu.se

For peer review only

Daniel S Olsson, Department of Internal Medicine and Clinical Nutrition, Institute of Medicine, Sahlgrenska Academy, University of Gothenburg, 405 30 Gothenburg, Sweden; Tel: +46708278671; E-mail: daniel.olsson@gu.se

Gudmundur Johannsson, Department of Internal Medicine and Clinical Nutrition, Institute of Medicine, Sahlgrenska Academy, University of Gothenburg, 405 30 Gothenburg, Sweden; Tel: +46313427052 E-mail: gudmundur.johannsson@medic.gu.se

Fredrik Nyberg, School of Public Health and Community Medicine, Institute of Medicine, Sahlgrenska Academy, University of Gothenburg, Gothenburg, Sweden; Tel: +46 31 7866289; E-mail: fredrik.nyberg.2@gu.se

Oskar Ragnarsson, Department of Internal Medicine and Clinical Nutrition, Institute of Medicine, Sahlgrenska Academy, University of Gothenburg, 405 30 Gothenburg, Sweden; Tel: +46 70 728 2229; E-mail: oskar.ragnarsson@medic.gu.se

Ethics approval statement

Ethical approval for this study was obtained from the Swedish Ethical Review Authority (2020-01800 with subsequent amendments).

For peer review only

Abstract

Objectives:

While glucocorticoid (GC) treatment initiated for COVID-19 reduces mortality, it is unclear whether GC treatment prior to COVID-19 affects mortality. Long-term GC use raises infection and thromboembolic risks. We investigated if patients with oral GC use prior to COVID-19 had increased mortality overall and by selected causes.

Design: Population-based observational cohort study

Settings: Population-based register data in Sweden

Participants: All patients infected with COVID-19 in Sweden from January 2020 to November 2021 (n=1,200,153).

Outcome measures: Any prior oral GC use was defined as ≥1 GC prescription during 12 months before index. High exposure was defined as ≥2 GC prescriptions with a cumulative prednisolone dose ≥750 mg or equivalent during 6 months before index. GC users were compared with COVID-19 patients who had not received GCs within 12 months before index. We used Cox proportional hazard models and 1:2 propensity score matching to estimate HRs and 95% CIs, controlling for the same confounders in all analyses.

Results:

3378 deaths occurred in subjects with any prior GC exposure (n=48,806; 6·9%) and 14,850 among non-exposed (n=1,151,347; 1·3%). Both high (1·98, 1·87–2·09) and any exposure (1·58, 1·52–1·65) to GCs were associated with overall death. Deaths from pulmonary embolism, sepsis, and COVID-19 were associated with high GC

For peer review only

exposure and, similarly but weaker, with any exposure. High exposure to GCs was associated with increased deaths caused by stroke and myocardial infarction.

Conclusion:

Patients on oral GC treatment prior to COVID-19 have increased mortality, particularly from pulmonary embolism, sepsis, and COVID-19.

Key words: glucocorticoids, mortality, adrenal insufficiency, COVID-19

Strengths and limitations of this study

- Utilizes a population-based design
- Employs adjusted Cox models and propensity score matching for confounder control.
- A large number of covariates, and a sizable comparison cohort were used
- Observational nature may still introduce residual bias and unmeasured confounding

For peer review only

INTRODUCTION

Oral glucocorticoids (GCs) are commonly prescribed for a variety of conditions, including inflammatory disorders as well as various allergic, respiratory, skin, and autoimmune conditions (1). In Sweden, 0.5% of the population receive long-term (>2 years) oral GC treatment (1), a treatment that is known to be associated with excess mortality mainly due to myocardial infarction, sepsis, and pulmonary embolism (2). Hypercortisolism is associated with a hypercoagulable state due to increased levels of procoagulant factors and impaired fibrinolysis (3). Patients receiving long-term GC treatment frequently also develop GC-induced adrenal insufficiency that renders them at increased risk of adrenal crisis, and subsequent death, during a severe infection (4).

Thromboembolic events, including pulmonary embolism and stroke, are common complications in patients with COVID-19 (5). Despite anticoagulation treatment, a notable proportion (16·7%) of patients with COVID-19 develop pulmonary embolism (5). COVID-19 patients may also develop arterial thrombosis, which can lead to serious outcomes such as stroke or myocardial infarction (6).

The use of oral GCs in COVID-19 patients has been a topic of research and debate. Current literature on GC therapy initiated for COVID-19 and effect on thromboembolic events warns that GC administration during COVID-19 may increase the risk of thromboembolic events, particularly in critically ill patients or those with pre-existing thrombotic risk factors (7). In May 2020, WHO published clinical guidelines on the management of COVID-19 and advised against the use of

For peer review only

GCs unless indicated for another reason (eg, exacerbation of asthma or chronic obstructive pulmonary disease) (8). At that time, high-dose GC therapy initiated for COVID-19 had been associated with delayed virus clearing, prolonged hospitalisation, and increased use of mechanical ventilation in patients with COVID-19 (9). In July 2020, the RECOVERY trial showed that dexamethasone treatment initiated in COVID-19 patients resulted in lower 28-day mortality among those requiring respiratory support (mechanical ventilation or oxygen alone) (10). Consequently, in September 2020, WHO revised the guidelines and recommended initiation of systemic GC treatment for patients with severe and critical COVID-19 (11). Nevertheless, GC treatment for COVID-19 patients is still controversial. GC therapy initiated for COVID-19 was related to increased death compared with conventional treatment in many observational studies, while beneficial effects on mortality were seen in randomised clinical trials (12). There is also a concern regarding the dose of GC therapy initiated for COVID-19, as higher doses have been shown to increase mortality rates in hypoxic patients not requiring ventilatory support (13). In the aforementioned studies, GC treatment was initiated for the treatment of COVID-19.

There is less known about how already present long-term GC treatment prior to COVID-19 effects outcome and mortality. An international study of patients with rheumatic disease receiving long-term GC treatment showed that prednisolone ≥10 mg/day prior to COVID-19 was associated with an increased hospitalisation rate (14). Chronic systemic GC therapy has also been associated with increased mortality in immunosuppressed patients with COVID-19 (15). Whether patients

For peer review only - http://bmjopen.bmj.com/site/about/guidelines.xhtml

receiving GC treatment for other conditions are at increased risk of death after developing COVID-19 has not been systematically studied.

Our hypothesis was that patients on oral GC treatment before being diagnosed with COVID-19 have an increased mortality from several conditions, including pulmonary embolism, myocardial infarction, stroke, sepsis, and COVID-19 itself compared with those who do not have GC treatment before COVID-19. To test this hypothesis, we investigated the association between prior GC treatment and the risk of mortality from these conditions among COVID-19 patients using population-based analysis.

METHODS

Study design, data sources, and study population

This was a register-based cohort study centred on the regularly updated SCIFI-PEARL (Swedish COVID-19 Investigation for Future Insights – a Population Epidemiology Approach using Register Linkage) project database,(16) which currently includes the entire Swedish population from 2015.

The study population consisted of individuals with COVID-19 who met the following criteria before Nov 30, 2021: (a) a positive test for SARS-CoV-2 in the National Database of Notifiable Communicable Diseases (SmiNet); (b) identified in the Swedish National Patient Register (NPR) with the International Classification of Diseases, revision 10 (ICD-10) codes U07.1 or U07.2 from a specialist visit or hospitalisation; or (c) identified in the Cause-of-Death Register with COVID-19 as an underlying or contributing cause of death. For each individual, their earliest date of

For peer review only

meeting any of these COVID-19 diagnosis criteria was considered their index date (baseline) in the study.

Additional data and sources used from the SCIFI-PEARL database included: socio-demographic data and dates of emigration or death from Statistics Sweden (Register of Total Population and the Longitudinal Integrated Database for Health Insurance and Labor Market Studies [LISA]) (17); prescribed medication data from the National Prescribed Drug Register (NPDR) (18); comorbidities from the NPR;(19) and intensive care data from the Swedish Intensive Care Register (SIR). Comprehensive data for each patient were obtained by linking their information across the different registers using the national personal identification number, as previously described (16). Ethical approval for this study was obtained from the Swedish Ethical Review Authority (2020-01800 with subsequent amendments).

Patient and Public Involvement: None

Exposures, outcomes, and follow-up

Data from the NPDR were used to identify oral GC users as well as GC dosage and formulation (capsules, drops, or tablets). Recent or ongoing oral GC treatment was defined in two ways: (a) any prior exposure as ≥1 dispensed prescriptions of oral GCs within 12 months before the index date; and (b) high exposure defined >2 dispensed prescriptions with a cumulative dose of prednisolone (Anatomical Therapeutic Chemical [ATC] code H02AB06) ≥750 mg, or equivalent amount (Supplementary Table S1) of betamethasone (H02AB01), dexamethasone (H02AB02), methylprednisolone (H02AB04), prednisone (H02AB07), hydrocortisone

For peer review only - http://bmjopen.bmj.com/site/about/guidelines.xhtml

(H02AB09), cortisone (H02AB10), deflazacort (H02AB13), or a combination of these within 6 months before the index date. All patients belonging to the "high exposure" group were thus also included in the "any prior exposure" group. These exposure definitions target recent use of oral GC, and for the “high exposure” more consistent and recent use, more likely extending into the future follow-up period, thus sharpening the identification of regular users and as such, patients at risk, and ensuring robust data analysis. The non-exposed comparison group included COVID-19 patients who had not received any oral GC prescriptions during the year before their index date. High exposure was defined as prednisolone ≥750 mg within 6 months before the index date, i.e. approximately ≥4-5 mg of prednisolone per day which is supraphysiological dose of GC (20). Doses below 5 mg of prednisolone per day have not been associated with an increased risk of death (21).

The primary outcomes were overall death (all-cause mortality) and cause-specific death according to ICD-10 classification due to pulmonary embolism (I26), sepsis (A40, A41, or R57.2), myocardial infarction (I21–I23), stroke (I60–I64, I630–I635, I638, or I639), and COVID-19 (U07.1 or U07.2). Outcomes of cause-specific death were identified using the Cause-of-Death Register with the diagnosis being either the underlying or contributing cause. Secondary outcomes were two measures of severe COVID-19, hospitalisation for COVID-19, and direct admission to an intensive care unit, each defined by U07.1 or U07.2 as primary or secondary diagnosis, respectively, in the NPR or SIR. The different outcomes were all analysed separately. For each outcome, all subjects were followed from the index date (COVID-19 infection date) until the earliest of the following: an event for the specific outcome, emigration, death, or end of the study period (Nov 30, 2021).

For peer review only - http://bmjopen.bmj.com/site/about/guidelines.xhtml

Baseline covariates

The following baseline information was extracted from relevant registers (for details see Table S2): (1) demographic factors from Statistics Sweden including age, sex, education, and employment; (2) comorbidities from the NPR as primary or secondary diagnoses for specialist outpatient or inpatient care from Jan 1, 2015 until the index date; and (3) dispensed prescribed medications from the NPDR during the year before the index date. Based on *a priori* selection, the following covariates were used for confounding adjustment: age, sex, education, employment, and relevant previous medical history (diabetes, deep vein thrombosis, pulmonary embolism, hypertension, stroke, ischaemic heart disease, heart failure, cancer, chronic obstructive pulmonary disease, rheumatologic conditions, and oral anticoagulation therapy). In sensitivity analyses, we considered additional covariates: adrenal insufficiency, rheumatoid arthritis, chronic lower respiratory diseases, other respiratory diseases principally affecting the interstitium, non-infective enteritis and colitis, dermatitis and eczema, inflammatory polyarthropathies, systemic connective tissue disorders (except rheumatoid arthritis), glomerular diseases, organ or tissue transplant, chronic liver disease, alcoholism and other substance abuse, and the following medications: inhaled corticosteroids, statins, angiotensin converting enzyme inhibitors (ACEIs), and opioids, (Table S2).

Statistical analysis

The exposed and non-exposed groups were characterised using descriptive statistics with continuous variables presented as mean and standard deviation (SD), and categorical variables as number (%). Differences between exposed and non-

For peer review only

exposed groups were characterised using standardised mean differences (SMDs). Kaplan-Meier curves were used to examine the outcomes over time during the study period. Cox proportional hazards regression was used to obtain hazard ratios (HRs) with 95% confidence intervals (CIs). In the main analysis, confounding was addressed by adjusting for the selected potential confounders in standard Cox models. In an alternative analysis approach, confounding was addressed by 1:2 matching on a propensity score that included the same set of confounders as in the main regression analysis. The propensity score was estimated by fitting a logistic regression model with the selected covariates as predictors and oral GC exposure as independent variables. A 1:2 matching on the propensity score was then performed and evaluated by assessment of SMD pre- and post-matching for all variables and by visually checking the propensity score overlap. Sensitivity analyses were also conducted incorporating additional potential confounders of interest, in particular greater detail regarding the underlying indications for oral GC. This sensitivity analysis could, however, instead suffer from potential overadjustment in relation to the study hypothesis of understanding the overall increased risk patients in these groups on oral GC faced when infected with COVID-19. Analyses were performed in SAS, and R statistical software version 4.04 and matching was implemented using the MatchIT package in R (22).

For peer review only

RESULTS

Demographic and clinical characteristics

A cohort of 1 200 153 individuals with COVID-19 was identified with 48 806 having any GC exposure the year before index, including 13 493 individuals with high GC exposure during the last 6 months (table 1). The exposed groups were older and included more women, whereas the non-exposed group had a more even sex distribution. Substantial differences between the exposed and non-exposed (SMDs >0·2) were found for age, sex, and multiple comorbidities including cardiovascular, respiratory, cancer, and rheumatic disease (table 1). There were 3378 deaths in patients with any GC exposure (6·9%), of which 2023 deaths occurred in patients with high exposure (15·0%) and 14 850 deaths in the non-exposed group (1·3%) (Table S3).

Association between GC exposure and death

Unadjusted cumulative incidence curves indicated that crude overall and cause-specific mortality was higher among GC users compared with non-users (figures 1 and 2).

In the main Cox regression analyses, the adjusted HR for all-cause mortality was 1·98 (95% CI 1·87–2·09) in the high exposure group and 1·58 (1·52–1·65) in patients with any exposure compared with the non-exposed group (figure 3). High exposure to oral GCs was also associated with significantly increased risks of death from pulmonary embolism, sepsis, stroke, and COVID-19. Any prior exposure was

For peer review only

similarly associated with death due to pulmonary embolism, sepsis, and COVID-19, but not significantly with fatal stroke (figure 3). The association for high exposure with death from myocardial infarction was similar in strength to that with fatal stroke, but marginally not significant (figure 3).

Association between GC exposure and severe COVID-19

Compared with the non-exposed group, the high exposure group demonstrated an elevated risk of hospitalisation for COVID-19 (adjusted HR 1·51, 95% CI 1·46–1·57) as well as intensive care unit admission (1·40, 1·26–1·55) (figure 3). There were also consistent but relatively weaker effects observed in patients with any prior exposure.

Alternative analysis approach with propensity score matching

Propensity score matching resulted in 91 878 non-exposed individuals matched to 47 737 individuals with any exposure and 23 898 individuals matched to 12 943 individuals with high exposure (Table S4). The numbers of outcome events in the matched cohorts are indicated in Table S5. In the matched cohorts, the covariates of the exposed and matched non-exposed groups were quite well balanced (Table S6) with good overlap of the propensity scores (Supplementary Figure S1) and low absolute SMDs between the matched cohorts (Table S5 and S6, Figure S2).

The results obtained from the propensity score matched analysis were, overall, in good agreement with the adjusted Cox model (figure 3). Propensity score matching indicated somewhat stronger effects of GC exposure on pulmonary embolism and myocardial infarction death, and weaker effects on sepsis and stroke death, severe COVID-19, and overall mortality in both high and any exposure categories (figure 3).

For peer review only

In this analysis, high oral GC exposure was not significantly associated with death from stroke but, conversely, it was significantly and more strongly associated with myocardial infarction deaths.

In a post-hoc sensitivity analysis, we sought to address potential confounding due to slight remaining covariate imbalances through adjustment and propensity score matching, utilizing an expanded set of covariates. This led to improved balance across most covariates (Table S7). Overall, the findings closely aligned with the main analysis except that ICU admission for COVID-19 was less common in GC users in this analysis (Table S8).

DISCUSSION

This nationwide population-based cohort study of approximately 1·2 million individuals who had contracted COVID-19 infection showed increased mortality following the infection for 48 806 oral GC users compared with non-users, especially from sepsis, stroke, and pulmonary embolism. Individuals receiving oral GC treatment prior to the infection also had an increased risk of COVID-19-related outcomes including hospitalisation, intensive care unit admission, and death. Furthermore, the results were robust in sensitivity analyses with extended confounding adjustment.

Comparison to other studies

Our study showed an increased risk of death from sepsis after contracting COVID-19 and the risk was greater in the high GC exposure group. Treatment with GCs is known to cause immunosuppression and GC users have increased susceptibility to

For peer review only

infections (23). Similarly, studies on endogenous hypercortisolism and GC users show a high mortality risk due to infection in other settings (2 24). For example, one-third of deaths in patients with Cushing disease is due to infections (24) and GC users have generally increased mortality from sepsis and pneumonia (2). There is also evidence that higher doses of GCs are associated with an increased risk of pneumonia and mortality in COVID-19 patients hospitalised with clinical hypoxia compared with standard low-dose GC treatment (13). Our results are also in line with observational data on patients with rheumatic diseases, showing that prednisolone dose ≥10mg/day prior to COVID-19 was associated with increased hospitalisation for COVID-19 (14).

We also found increased mortality from pulmonary embolism in GC users after contracting COVID-19. A generally increased incidence of thromboembolism in patients with endogenous hypercortisolism is known (25). A recent study showed that patients with Cushing's syndrome have a generally increased incidence of stroke and thromboembolism (25). Studies on GC users have reported similar findings with an approximately two-fold increased risk of both pulmonary embolism and deep vein thrombosis (26). The risk of comparable events after COVID-19 in the current study is similar in magnitude.

GC treatment was not significantly associated with death from stroke in our study, but it was significantly and more strongly associated with myocardial infarction deaths. The association was stronger in the high exposure group compared with the any prior exposure group. Our findings are in line with a population-based, nested, case-control study from the UK on oral GC and risk of cardiovascular and

For peer review only

cerebrovascular disease based on data from 1988 to 1998 (27). The study showed increased risk of ischaemic heart disease (odds ratio [OR] 1·20, 95% CI 1·11–1·29) in current GC users, but not for increased risk of stroke/transient ischaemic attack (OR 0·95, 95% CI 0·89–1·01) (27).

We used both adjusted Cox models and propensity score matching to control for potential confounders in an effort to obtain unbiased estimates of the impact of oral GC use on severe outcomes. To clarify and understand imbalances in characteristics between the exposure groups before and after propensity score matching, we evaluated SMDs and achieved good balance after matching (28). It may be noted that the estimand for the adjusted Cox model is the conditional average treatment effect for the treated (ATE), ie, the effect if everyone were to be on treatment with oral GCs, conditional on their characteristics, while the estimand for the matched analysis is the average treatment effect for the treated (ATT), ie, the effect among those actually receiving oral GC treatment (29). ATE can be used to decide whether to mandate treatment for all patients, whereas ATT can be used to decide whether to withhold it from patients who would otherwise receive it. Nevertheless, the two methods produced comparable results that are clinically relevant and complementary.

For peer review only

Strengths and limitations of this study

This study has several strengths including a population-level sample, a large number of covariates, and a large comparison cohort which minimizes the risk of selection bias. However, since our study is observational, the analyses may still be subject to residual bias and unmeasured confounding, and thus the results require cautious

interpretation. All GC users have an underlying disease that may increase mortality risk, potentially confounding the results. We mitigated this concern by adjusting for carefully pre-selected covariates, by conducting a sensitivity analysis with expanded covariate selection, and by using two analytical approaches. The results of all analyses were fairly consistent, supporting the robustness of the findings. The sensitivity analyses, however, suggested that oral GC per se may not be the driver for increased COVID-19-related ICU admission, although this is speculative and should be interpreted with caution due to the more limited power for this rare outcome and the risk for overadjustment in these analyses.

With regard to stage of severity and exposure to GC, we assessed both "any" exposure and "high" exposure to address differences in the severity of the underlying condition warranting oral GC treatment. In our study, we relied on a prescription register that only provided information about dispensed prescriptions, and thus adherence to treatment and actual use must be assumed (as is generally the case). Our study exclusively focused on the oral administration of GCs and their impact on COVID-19. While inhaled GCs have been studied in Swedish context (30), our study places a deliberate emphasis on the oral route. Other administration routes for GC, such as topical and parenteral administrations, were intentionally excluded from our investigations, as they are likely less directly pertinent to COVID-19.

Unanswered questions and future research

The causal relationship between GC use and mortality remains a topic yet to be fully elucidated, primarily due to the fact that all long-term GC users have an underlying disease that may heighten mortality risk. Our study provides a comprehensive

For peer review only

overview of excess mortality in this population. However, it's crucial to highlight that studying each underlying medical condition necessitates specific analytical considerations. To enhance our understanding of the factors contributing to excess mortality, future research should prioritize dissecting the precise impact of prolonged GC use on these underlying conditions. Our mortality estimates essentially represent a weighted average effect over the pandemic period, primarily for power and precision reasons. Larger cohorts in future studies may help reveal wave-specific effects.

Fundamentally, GC treatment suppresses the hypothalamic-pituitary-adrenal axis and can cause GC-induced adrenal insufficiency (4 31). Undiagnosed and untreated GC-induced adrenal insufficiency can lead to a life-threatening adrenal crisis. To prevent adrenal crisis, GC users should know how to increase their GC dose during sick days and during intercurrent illness (32). Awareness of GC-induced adrenal insufficiency is increasing. In 2020, guidance on the prevention of adrenal crisis during the COVID-19 pandemic was published and recommended that GC users increase their daily GC dose during COVID-19 infection and to take GC every 6–12 hours (depending on the type of GC used) (32). GC users who need to be admitted to hospital because of COVID-19 should receive GC intravenously (32).

CONCLUSION

Patients receiving GC treatment prior to COVID-19 infection face excess mortality in the aftermath of their acute COVID-19 infection, particularly from sepsis, pulmonary embolism, and COVID-19. These patients also have a high risk of intensive care unit

For peer review only

admission and death due to COVID-19, and close monitoring and clinical attention may thus prevent their death.

Acknowledgements: We thank Peter Todd of Tajut Ltd. (Kaiapoi, New Zealand) for third-party editorial support, for which he received financial compensation from ALF funding.

Authors contributions

OR, ME, BK, FN, HL, DSO, and GJ were involved in the conceptualisation and design of the study. FN and HL acquired the data. BK and HL performed statistical analysis. OR, ME, BK, FN, HL, DSO, and GJ were involved with interpretation of the data and initial drafting of the manuscript. All authors participated in the critical appraisal, further interpretation, revision, and approved the final manuscript.

Competing interests statement

GJ has served as consultant for Ascendis Pharma, Astra Zeneca, and Novo Nordisk, and has received lecture fees from Novo Nordisk and Pfizer. DSO has received unrestricted project grants from Pfizer and is an employee at AstraZeneca as of August 8, 2021. FN holds some AstraZeneca shares. BK, ME, HL, and OR have nothing to disclose.

Funding information

ALF agreement (ALFGBG-938453, ALFGBG-971130, ALFGBG-978954, ALFGBG-966066), Swedish Research Council (2020-02828, 2019-01112), and Swedish Heart-Lung Foundation (20210030, 20210581).

Role of the funding sources

The funders of the study had no role in study design, data collection, data analysis, data interpretation, or writing of this manuscript.

For peer review only

Data availability statement

The individual level data used in this study are pseudonymised and sourced from Swedish healthcare registers. Interested researchers can obtain access to the data from the appropriate Swedish public data holders subject to obtaining ethics approval for the research and adhering to all relevant legislation, processes, and data protection protocols.

For peer review only

References

1. Einarsdottir MJ, Ekman P, Trimpou P, et al. High prescription rate of oral glucocorticoids in children and adults: A retrospective cohort study from Western Sweden. *Clin Endocrinol (Oxf)* 2020;92(1):21-28.
2. Einarsdottir MJ, Ekman P, Molin M, et al. High mortality rate in oral glucocorticoid users: a population-based matched cohort study. *Front Endocrinol (Lausanne)* 2022;13:918356.
3. Manetti L, Bogazzi F, Giovannetti C, et al. Changes in coagulation indexes and occurrence of venous thromboembolism in patients with Cushing's syndrome: results from a prospective study before and after surgery. *Eur J Endocrinol* 2010;163(5):783-91.
4. Broersen LH, Pereira AM, Jorgensen JO, et al. Adrenal insufficiency in corticosteroids use: Systematic review and meta-analysis. *J Clin Endocrinol Metab* 2015;100(6):2171-80.
5. Helms J, Tacquard C, Severac F, et al. High risk of thrombosis in patients with severe SARS-CoV-2 infection: a multicenter prospective cohort study. *Intensive Care Med* 2020:1-10.
6. Katsoularis I, Fonseca-Rodríguez O, Farrington P, et al. Risk of acute myocardial infarction and ischaemic stroke following COVID-19 in Sweden: a self-controlled case series and matched cohort study. *Lancet* 2021;398(10300):599-607.
7. Sarfraz A, Sarfraz Z, Razzack AA, et al. Venous Thromboembolism, Corticosteroids and COVID-19: A Systematic Review and Meta-Analysis. *Clin Appl Thromb Hemost* 2021;27:1076029621993573.
8. World Health Organization. Clinical management of COVID-19: interim guidance, 27 May 2020 2020 [Available from: https://apps.who.int/iris/handle/10665/332196 accessed June 14 2023.
9. Li H, Chen C, Hu F, et al. Impact of corticosteroid therapy on outcomes of persons with SARS-CoV-2, SARS-CoV, or MERS-CoV infection: a systematic review and meta-analysis. *Leukemia* 2020;34(6):1503-11.
10. RECOVERY Collaborative Group; Horby P, Lim WS, Emberson JR, et al. Dexamethasone in hospitalized patients with Covid-19. N Engl J Med 2021; 25;384: 693–704.
11. World Health Organization. Corticosteroids for COVID-19, Living guidance 2 September 2020 2020 [Available from: https://www.who.int/publications/i/item/WHO-2019-nCoV-Corticosteroids-2020.1 accessed 8 October 2020.
12. Patel C, Parmar K, Patel D, et al. Effect of corticosteroid therapy on mortality in COVID-19 patients-A systematic review and meta-analysis. *Rev Med Virol* 2022;32(5):e2386.
13. Higher dose corticosteroids in patients admitted to hospital with COVID-19 who are hypoxic but not requiring ventilatory support (RECOVERY): a randomised, controlled, open-label, platform trial. *Lancet* 2023;401(10387):1499-507.
14. Gianfrancesco M, Hyrich KL, Al-Adely S, et al. Characteristics associated with hospitalisation for COVID-19 in people with rheumatic disease: data from the COVID-19 Global Rheumatology Alliance physician-reported registry. *Ann Rheum Dis* 2020;79(7):859-66.
15. Calderón-Parra J, Cuervas-Mons V, Moreno-Torres V, et al. Influence of chronic use of corticosteroids and calcineurin inhibitors on COVID-19 clinical outcomes: analysis of a nationwide registry. *Int J Infect Dis* 2022;116:51-58.
16. Nyberg F, Franzén S, Lindh M, et al. Swedish Covid-19 Investigation for Future Insights - A Population Epidemiology Approach Using Register Linkage (SCIFI-PEARL). *Clin Epidemiol* 2021;13:649-59.

For peer review only - http://bmjopen.bmj.com/site/about/guidelines.xhtml

17. Ludvigsson JF, Svedberg P, Olén O, et al. The longitudinal integrated database for health insurance and labour market studies (LISA) and its use in medical research. *Eur J Epidemiol* 2019;34(4):423-37.

18. The Swedish Prescribed Drug Register. Socialstyrelsen. [cited 2023 September 11]. Available from: https://www.socialstyrelsen.se/statistik-och-data/register/lakemedelsregistret/ [

19. The National Patient Register. Socialstyrelsen. [cited 2023 September 8]. Available from: https://www.socialstyrelsen.se/statistik-och-data/register/patientregistret/

20. Borresen SW, Klose M, Glintborg D, et al. Approach to the patient with glucocorticoid-induced adrenal insufficiency. *J Clin Endocrinol Metab* 2022

21. Movahedi M, Costello R, Lunt M, et al. Oral glucocorticoid therapy and all-cause and cause-specific mortality in patients with rheumatoid arthritis: a retrospective cohort study. *Eur J Epidemiol* 2016;31(10):1045-55.

22. Stuart EA, King G, Imai K, et al. MatchIt: nonparametric preprocessing for parametric causal inference. *Journal of statistical software* 2011

23. Stuck AE, Minder CE, Frey FJ. Risk of infectious complications in patients taking glucocorticosteroids. *Rev Infect Dis* 1989;11(6):954-63.

24. Ragnarsson O, Olsson DS, Papakokkinou E, et al. Overall and Disease-Specific Mortality in Patients With Cushing Disease: A Swedish Nationwide Study. *J Clin Endocrinol Metab* 2019;104(6):2375-84.

25. Papakokkinou E, Olsson DS, Chantzichristos D, et al. Excess morbidity persists in patients with Cushing's disease during long-term remission - a Swedish nationwide study. *J Clin Endocrinol Metab* 2020

26. Johannesdottir SA, Horvath-Puho E, Dekkers OM, et al. Use of glucocorticoids and risk of venous thromboembolism: a nationwide population-based case-control study. *JAMA Intern Med* 2013;173(9):743-52.

27. Souverein PC, Berard A, Van Staa TP, et al. Use of oral glucocorticoids and risk of cardiovascular and cerebrovascular disease in a population based case-control study. *Heart* 2004;90(8):859-65.

28. Austin PC. Balance diagnostics for comparing the distribution of baseline covariates between treatment groups in propensity-score matched samples. *Statistics in medicine* 2009;28(25):3083-107.

29. Austin PC. The use of propensity score methods with survival or time-to-event outcomes: reporting measures of effect similar to those used in randomized experiments. *Statistics in medicine* 2014;33(7):1242-58.

30. Labor M, Kirui BK, Nyberg F, et al. Regular Inhaled Corticosteroids Use May Protect Against Severe COVID-19 Outcome in COPD. *Int J Chron Obstruct Pulmon Dis* 2023;18:1701-12.

31. Schlaghecke R, Kornely E, Santen RT, et al. The effect of long-term glucocorticoid therapy on pituitary-adrenal responses to exogenous corticotropin-releasing hormone. *N Engl J Med* 1992;326(4):226-30.

32. Arlt W, Baldeweg SE, Pearce SHS, et al. Endocrinology in the time of COVID-19: Management of adrenal insufficiency. *Eur J Endocrinol* 2020;183(1):G25-g32.

For peer review only

Table 1: Baseline characteristics of individuals with COVID-19 infection in Sweden from January 2020 to November 2021 without and with glucocorticoid exposure prior to the infection

	Non-exposed (n=1 151 347)	Any prior GC exposure* (n=48 806)		High GC exposure† (n=13 497)	
			SMD *vs* non-exposed		SMD *vs* non-exposed
Age (years)	37·9 (19·2)	51·4 (21·2)	0·666	62·4 (19·7)	1·257
Age category (years)			0·672		1·276
0–9	56 461 (4·9%)	930 (1·9%)		91 (0·7%)	
10–19	171 590 (14·9%)	3056 (6·3%)		284 (2·1%)	
20–29	200 477 (17·4%)	4491 (9·2%)		567 (4·2%)	
30–39	200 414 (17·4%)	5882 (12·1%)		925 (6·9%)	
40–49	205 427 (17·8%)	8045 (16·5%)		1514 (11·2%)	
50–59	167 108 (14·5%)	8993 (18·4%)		2249 (16·7%)	
60–69	79 704 (6·9%)	6396 (13·1%)		188 (16·2%)	
70–79	37 797 (3·3%)	5725 (11·7%)		2680 (19·9%)	
80–89	23 055 (2·0%)	4123 (8·4%)		2302 (17·1%)	
≥90	9314 (0·8%)	1165 (2·4%)		697 (5·2%)	
Sex			0·149		0·147
Male	566 579 (49·2%)	20 407 (41·8%)		5659 (41·9%)	
Female	584 768 (50·8%)	28 399 (58·2%)		7838 (58·1%)	
Birth country			0·105		0·199
Sweden	883 118 (76·7%)	38 515 (78·9%)		10 923 (80·9%)	
Nordic	18 577 (1·6%)	1285 (2·6%)		484 (3·6%)	
Europe except Nordics	83 455 (7·2%)	3142 (6·4%)		779 (5·8%)	
Other	166 197 (14·4%)	5864 (12·0%)		1311 (9·7%)	
Employment status			0·453		0·888
Employed	838 341 (72·8%)	31 232 (64·0%)		6432 (47·7%)	
Unemployed	172 951 (15·0%)	15 328 (31·4%)		6857 (50·8%)	
Unknown	140 055 (12·2%)	2246 (4·6%)		208 (1·5%)	

For Peer Review only

Education level			0·300		0·501
Primary	173 194 (15·0%)	9817 (20·1%)		3672 (27·2%)	
Secondary	426 657 (37·1%)	19 950 (40·9%)		5656 (41·9%)	
Higher	376 185 (32·7%)	15 855 (32·5%)		3714 (27·5%)	
Unknown	175 311 (15·2%)	3184 (6·5%)		455 (3·4%)	
Comorbidities					
Pulmonary embolism	7977 (0·7%)	1676 (3·4%)	0·194	853 (6·3%)	0·310
Stroke	12 140 (1·1%)	1679 (3·4%)	0·161	852 (6·3%)	0·282
Deep vein thrombosis	1085 (0·1%)	301 (0·6%)	0·088	97 (0·7%)	0·098
Hypertension	86 847 (7·5%)	13 486 (27·6%)	0·547	6255 (46·3%)	0·972
Ischaemic heart disease	26 541 (2·3%)	4370 (9·0%)	0·291	2147 (15·9%)	0·487
Overall CV disease	149 594 (13·0%)	19 033 (39·0%)	0·621	8293 (61·4%)	1·158
Heart failure	18 043 (1·6%)	4456 (9·1%)	0·341	2347 (17·4%)	0·561
Asthma	37 185 (3·2%)	7841 (16·1%)	0·445	2026 (15·0%)	0·418
COPD	8731 (0·8%)	3856 (7·9%)	0·356	1700 (12·6%)	0·488
Emphysema	793 (0·1%)	392 (0·8%)	0·112	199 (1·5%)	0·161
Diabetes mellitus	42 598 (3·7%)	5478 (11·2%)	0·289	2638 (19·5%)	0·510
Rheumatic disease	8727 (0·8%)	6568 (13·5%)	0·510	4109 (30·4%)	0·897
Cancer	44 717 (3·9%)	7666 (15·7%)	0·406	3511 (26·0%)	0·653
Prescribed medications					
Inhaled corticosteroids	120 572 (10·5%)	17 815 (36·5%)	0·645	4258 (31·5%)	0·536
Oral anticoagulants	57 018 (5·0%)	8693 (17·8%)	0·413	3992 (29·6%)	0·689
Statins	101 469 (8·8%)	11 096 (22·7%)	0·389	4556 (33·8%)	0·640
ACEIs	67 321 (5·8%)	6969 (14·3%)	0·283	2860 (21·2%)	0·460
Opioids	112 707 (9·8%)	12 505 (25·6%)	0·424	4523 (33·5%)	0·601

Data are n (%) or mean (SD). *One or more prescriptions of oral GCs within 12 months before the COVID-19 infection date. †Two or more prescriptions and prednisolone ≥750 mg or equivalent amount of betamethasone, dexamethasone, methylprednisolone, prednisone, hydrocortisone, cortisone, or deflazacort within 6 months before COVID-19 infection date. Note this group is a subset of any prior exposure. ACEI, angiotensin-converting enzyme inhibitor; COPD, chronic obstructive pulmonary disease; CV, cardiovascular; GC, glucocorticoid; SMD, standardised mean difference.

For peer review only

Figure legends

Figure 1: **Unadjusted cumulative incidence of all-cause (a) and cause-specific (b–f) mortality among COVID-19 patients in Sweden with any prior exposure* to oral glucocorticoids compared to non-exposed, from COVID-19 infection date to Nov 30, 2021**

GC, glucocorticoids. *Any prior GC exposure equals ≥1 prescription of oral GC within 12 months before the COVID-19 infection date.

Figure 2: **Unadjusted cumulative incidence of all-cause (a) and cause-specific (b–f) mortality among COVID-19 patients with any prior exposure* to oral glucocorticoids compared to non-exposed, from COVID-19 infection date to Nov 30, 2021**

GC, glucocorticoids. *High GC exposure equals ≥2 prescriptions with a total of ≥750 mg of prednisolone or equivalent within 6 months before COVID-19 infection date. This group is a subset of any prior exposure see figure 1).

Figure 3: **Adjusted Cox regression analysis and propensity score matched analysis comparing the risk of all-cause and cause-specific mortality between COVID-19 patients with prior exposure to oral glucocorticoids and non-exposed patients**

CI, confidence interval; HR, hazard ratio; ICU, intensive care unit; PS, propensity score. Any prior GC exposure equals ≥1 prescription of oral GC within 12 months before the COVID-19 infection date. High GC exposure equals ≥2 prescriptions with a total of ≥750 mg of prednisolone or equivalent within 6 months before COVID-19

For peer review only

infection date. The latter group is a subset of any prior exposure. *Adjusted for covariates; †Propensity score matched on covariates.

For peer review only

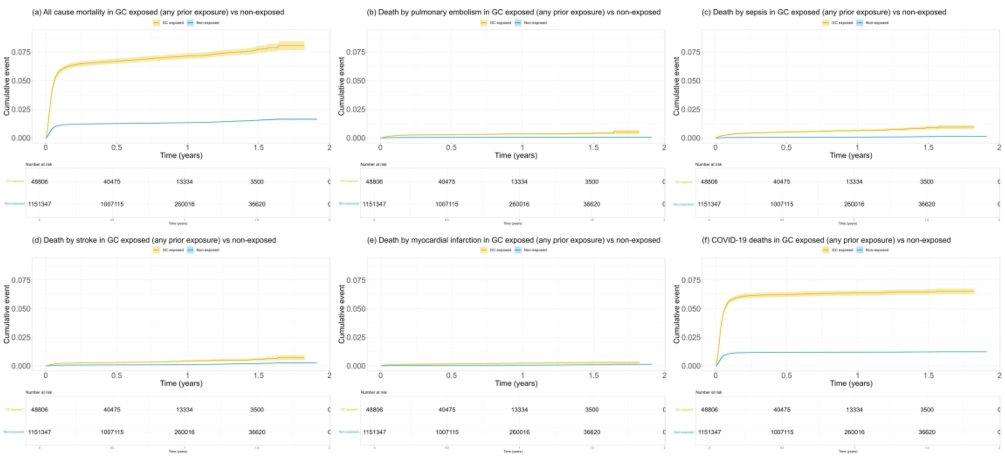

Figure 1: Unadjusted cumulative incidence of all-cause (a) and cause-specific (b–f) mortality among COVID-19 patients in Sweden with any prior exposure* to oral glucocorticoids compared to non-exposed, from COVID-19 infection date to Nov 30, 2021
GC, glucocorticoids. *Any prior GC exposure equals ≥1 prescription of oral GC within 12 months before the COVID-19 infection date.

338x154mm (300 x 300 DPI)

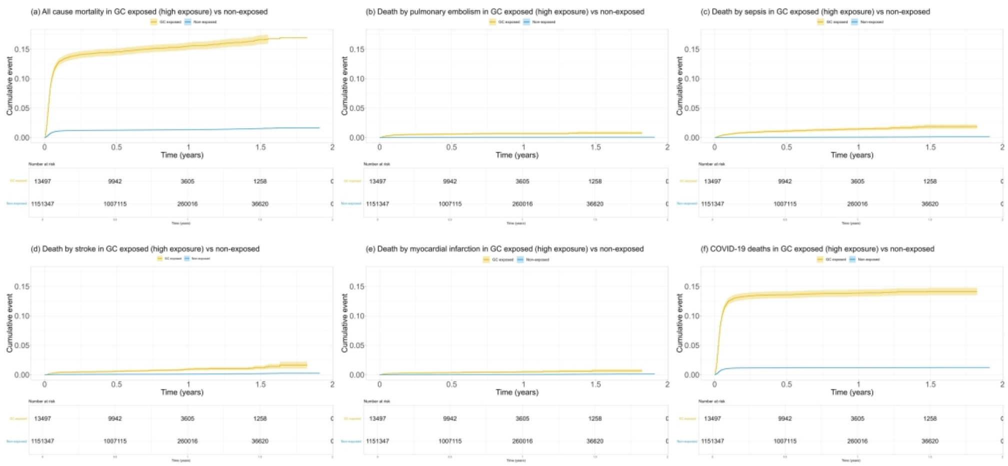

Figure 2: Unadjusted cumulative incidence of all-cause (a) and cause-specific (b–f) mortality among COVID-19 patients with any prior exposure* to oral glucocorticoids compared to non-exposed, from COVID-19 infection date to Nov 30, 2021
GC, glucocorticoids. *High GC exposure equals ≥2 prescriptions with a total of ≥750 mg of prednisolone or equivalent within 6 months before COVID-19 infection date. This group is a subset of any prior exposure see figure 1).

338x154mm (300 x 300 DPI)

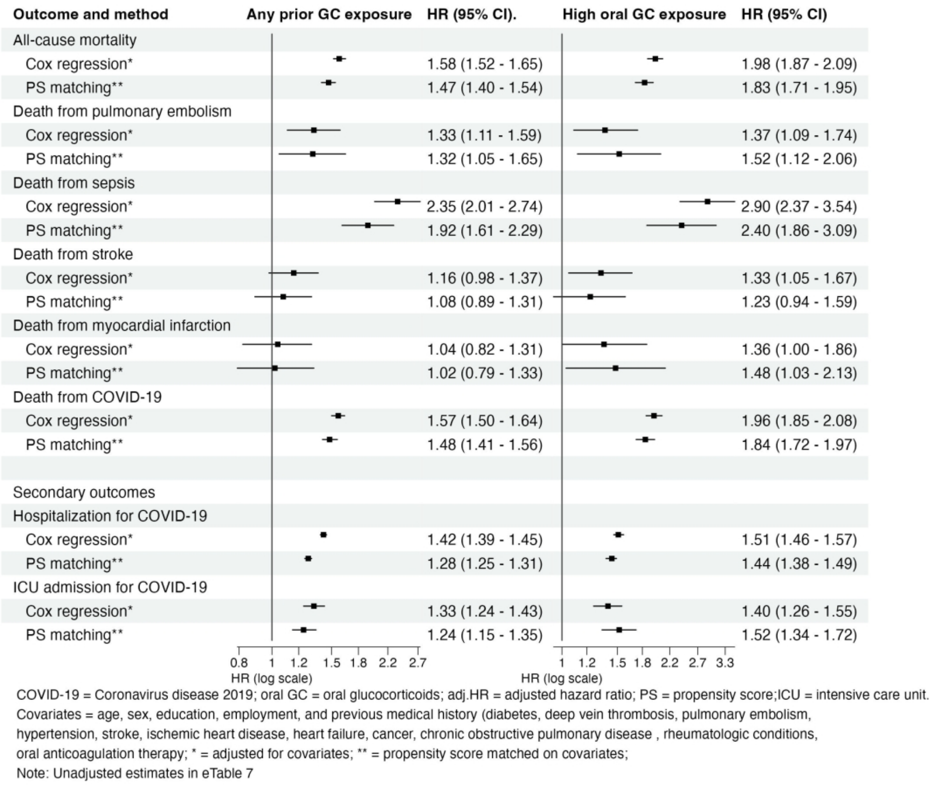

Figure 3: Adjusted Cox regression analysis and propensity score matched analysis comparing the risk of all-cause and cause-specific mortality between COVID-19 patients with prior exposure to oral glucocorticoids and non-exposed patients

CI, confidence interval; HR, hazard ratio; ICU, intensive care unit; PS, propensity score. Any prior GC exposure equals ≥1 prescription of oral GC within 12 months before the COVID-19 infection date. High GC exposure equals ≥2 prescriptions with a total of ≥750 mg of prednisolone or equivalent within 6 months before COVID-19 infection date. The latter group is a subset of any prior exposure. *Adjusted for covariates; †Propensity score matched on covariates.

971x932mm (72 x 72 DPI)

Supplementary appendix

Supplementary Table S1: **Dose equivalents used in the calculation of equivalence to prednisolone............ 2**

Supplementary Table S2: **Covariates selected in the evaluation of the effect of glucocorticoid treatment on death in COVID-19 patients in the Swedish population-based cohort of COVID-19 patients from COVID-19 infection date to Nov 30, 2021... 3**

Supplementary Table S3: **Outcome events in individuals with COVID-19 infection in the Swedish population during the period 2015 to COVID-19 infection date until Nov 30, 2021 stratified by level of exposure to oral glucocorticoids (non-exposed, high exposure in the 6 months before infection, or any exposure 12 months before infection and overall)... 5**

Supplementary Table S4: **Baseline characteristics of individuals with COVID-19 infection in the Swedish population during the period 2015 to COVID-19 infection date (comorbidities) or 1 year before COVID-19 infection date (medications) until Nov 30, 2021, stratified by level of exposure to oral glucocorticoids (high exposure in the 6 months before infection or any exposure 12 months before infection), with standardized mean differences between exposed and 1:2 propensity score matched non-exposed 6**

Supplementary Table S5: **Outcome events in propensity score matched cohorts of individuals with COVID-19 infection in the Swedish population during the period 2015 to COVID-19 infection date until Nov 30, 2021 stratified by level of exposure to oral glucocorticoids (non-exposed, high exposure in the 6 months before infection, or any exposure 12 months before infection and overall).................................... 8**

Supplementary Table S6: **Standardised mean differences between the 15 a priori selected covariates of individuals with COVID-19 infections in the Swedish population during the period 2015 to COVID-19 infection date (comorbidities) or 1 year before COVID-19 infection date (medications) in those exposed to oral glucocorticoids compared with those non-exposed before and after propensity score matching, main analysis.. 9**

Supplementary Table S7: **Standardised mean differences between the 15 a priori selected plus added‡ covariates in sensitivity analysis of individuals with COVID-19 infections in the Swedish population during the period 2015 to COVID-19 infection date (comorbidities) or 1 year before COVID-19 infection date (medications) in those exposed to oral glucocorticoids compared with those non-exposed before and after propensity score matching.. 10**

Supplementary Table S8: **Unadjusted, as well as adjusted from main and sensitivity analysis of the risk of various causes of death and all-cause mortality between COVID-19 patients who had any or high prior exposure to oral glucocorticoids and non-exposed individuals, hazard ratios from Cox regression analyses and propensity score matched analysis with 95% confidence intervals.. 11**

Supplementary Figure S1: **Propensity score balance before (a, c) and after (b, d) performing propensity score matching on all 15 covariates for individuals with COVID-19 infections in the Swedish population between 2015 and the COVID-19 infection date comparing those exposed to oral glucocorticoids (pink) with those who were non-exposed (blue) in those with any (a, b) or high glucocorticoid exposure (c, d) .. 13**

Supplementary Figure S2: Standardised mean differences between the covariates of individuals with COVID-19 infections in the Swedish population during the period 2015 to COVID-19 infection date (comorbidities) or 1 year before COVID-19 infection date (medications) in the exposed to oral glucocorticoids compared with non-exposed before (red circles) and after (blue triangles) propensity score matching among those with (a) any or (b) high glucocorticoid exposure. 14

For peer review only

Supplementary Table S1: **Dose equivalents used in the calculation of equivalence to prednisolone**

ATC code	Recommended international non-proprietary drug name	Equivalent dose to 1 mg of prednisolone (mg)	Equivalent dose to 750 mg of prednisolone (mg)*
H02AB01	Betamethasone	8·333	6250·0
H02AB02	Dexamethasone	6·666	5000·0
H02AB04	Methylprednisolone	1·25	937·5
H02AB06	Prednisolone	1	750·0
H02AB07	Prednisone	1	750·0
H02AB09	Hydrocortisone	0·25	187·5
H02AB10	Cortisone	0·2	150·0
H02AB13	Deflazacort	0·666	500·0

ATC, Anatomical Therapeutic Chemical. *The definition of high oral glucocorticoid exposure was ≥2 prescriptions of prednisolone ≥750 mg or equivalent within 6 months before COVID-19 infection date. The corresponding equivalent amount to prednisolone 750 mg or the other oral glucocorticoids is shown on this column.

For peer review only

Supplementary Table S2: **Covariates selected in the evaluation of the effect of glucocorticoid treatment on death in COVID-19 patients in the Swedish population-based cohort of COVID-19 patients from COVID-19 infection date to Nov 30, 2021**

**Covariate**	**Units and categories**	**Register source; description**	**Lookback window before COVID-19 infection date (index date)**
Sociodemographic characteristics			
Age	As a continuous variable and in 10-year categories	RTB	At Jan 1, 2020
Sex	Male, female	RTB	At Jan 1, 2020
Education	Primary, secondary, higher, or unknown	LISA: primary education included pre-secondary education <9, 9, or >9 years. Secondary educated included 2-year high school education, secondary education <3 years or >3 years, and higher education included those with post-high school education. Unknown includes children with no education information or individuals with no relevant education (eg, immigrants).	At Jan 1, 2020
Employment status	Employed, unemployed, or unknown	LISA: created using the variable "employment" and stratified by age where: "employed" was either employed or with entrepreneurial income, and "unemployed" was the unemployed and without entrepreneurial income and includes pensioners. Unknown includes individuals with no information on employment.	At Jan 1, 2020
Comorbidity	**Units/categories**	**NPR: ICD-10 codes, any mention of condition as primary or secondary diagnosis for a hospitalisation or ≥1 visit at any time**	Jan 1, 2015 to index date
Pulmonary embolism	Yes/No	NPR: I26	Jan 1, 2015 to index date
Stroke	Yes/No	NPR: I61 I63 I649 I691 I693 I694 I698	Jan 1, 2015 to index date
Deep vein thrombosis	Yes/No	NPR: I80–I82	Jan 1, 2015 to index date
Hypertension	Yes/No	NPR: I10–I15	Jan 1, 2015 to index date
Ischaemic heart disease	Yes/No	NPR: I20 –I25	Jan 1, 2015 to index date
Overall cardiovascular disease	Yes/No	NPR: I00–I99	Jan 1, 2015 to index date
Heart failure	Yes/No	NPR: I50	Jan 1, 2015 to index date
Asthma	Yes/No	NPR: J45	Jan 1, 2015 to index date
COPD	Yes/No	NPR: J44	Jan 1, 2015 to index date
Emphysema	Yes/No	NPR: J43	Jan 1, 2015 to index date
Diabetes	Yes/No	NPR: E10, E11, E13, E14	Jan 1, 2015 to index date
Rheumatic disease	Yes/No	NPR: M05, M06, M31, M35	Jan 1, 2015 to index date
Cancer	Yes/No	NPR: C00–C97	Jan 1, 2015 to index date
Covariates for sensitivity analysis*			
Adrenal insufficiency	Yes/No	NPR: E271, E272, E230, E230A, E230B, E230C, E230D, E230E, E230F, E230W, E230X, E893, E310	Jan 1, 2015 to index date
Rheumatoid arthritis	Yes/No	NPR: M050-M053, M058, M058A, M058B, M058C, M058D, M058F, M058G, M058H, M058L, M058M, M058N, M058X, M059, M059L, M059M, M059N, M06-P, M060, M060L, M060M, M060N, M061-M064, M068, M068L, M068M, M068N, M069, M069A, M069B, M069C, M069D, M069F, M069G, M069H, M069X	Jan 1, 2015 to index date
Chronic lower respiratory diseases	Yes/No	NPR: J41-J47	Jan 1, 2015 to index date
Other respiratory diseases principally affecting the interstitium	Yes/No	NPR: J80-J84	Jan 1, 2015 to index date
Non-infective enteritis and colitis,	Yes/No	NPR: K50-K52	Jan 1, 2015 to index date

For peer review only

Dermatitis and eczema	Yes/No	NPR:L20-L30	Jan 1, 2015 to index date
Inflammatory polyarthropathies	Yes/No	NPR: M05-M14	Jan 1, 2015 to index date
Systemic connective tissue disorders (except RA M31 and M35)	Yes/No	NPR: M30-M36 (excluding M35.3, M31.5 and M31.6)	Jan 1, 2015 to index date
Glomerular diseases,	Yes/No	NPR: N01-N08	Jan 1, 2015 to index date
Organ or tissue transplant	Yes/No	NPR: Z940-Z944, Z948	Jan 1, 2015 to index date
Chronic liver disease	Yes/No	NPR: K72-K75	Jan 1, 2015 to index date
Alcoholism and other substance abuse	Yes/No	NPR: F10-F19	Jan 1, 2015 to index date
Prescribed medications	**Units/categories**	**NPDR: ATC codes. At least one filled prescription in lookback window**	
Inhaled corticosteroids*	Yes/No	R03BA, R03AK, R03AL08, R03AL09	1 year before index
Oral anticoagulants	Yes/No	B01AA, B01AF	1 year before index
Statins*	Yes/No	C10AA	1 year before index
ACEIs*	Yes/No	C09A	1 year before index
Opioids*	Yes/No	N02AB	1 year before index

ACEIs, angiotensin-converting enzyme inhibitors; ATC, Anatomical Therapeutic Chemical; BMI, body mass index; COPD, chronic obstructive pulmonary disease; ICD-10, International Classification of Diseases, version 10; LISA, Longitudinal Integrated Database for Health Insurance and Labor Market Studies; NPDR, Swedish National Prescribed Drug Register; NPR, Swedish National Patient Register; RTB, Register of Total Population; SNAR, Swedish National Airways Register; *16 covariates for sensitivity analyses.

For peer review only

***Supplementary Table S3:* Outcome events in individuals with COVID-19 infection in the Swedish population during the period 2015 to COVID-19 infection date until Nov 30, 2021 stratified by level of exposure to oral glucocorticoids (non-exposed, high exposure in the 6 months before infection, or any exposure 12 months before infection and overall)**

	No=0/Yes=1	Non-exposed (n=1 151 347)	Any GC exposure* (n=48 806)	High GC exposure† (n=13 497)	Total study population (n=1 200 153)
All-cause mortality					
	0	1 136 497 (98·7%)	45 428 (93·1%)	11 474 (85·0%)	1 181 925 (98·5%)
	1	14 850 (1·3%)	3378 (6·9%)	2023 (15·0%)	18 228 (1·5%)
Death from pulmonary embolism					
	0	1 150 769 (99·9%)	48 654 (99·7%)	13 417 (99·4%)	1 199 423 (99·9%)
	1	578 (0·1%)	152 (0·3%)	80 (0·6%)	730 (0·1%)
Death from sepsis					
	0	1 150 576 (99·9%)	48 520 (99·4%)	13 335 (98·8%)	1 199 096 (99·9%)
	1	771 (0·1%)	286 (0·6%)	162 (1·2%)	1057 (0·1%)
Death from myocardial infarction					
	0	1 150 755 (99·9%)	48 707 (99·8%)	13 440 (99·6%)	1 199 462 (99·9%)
	1	592 (0·1%)	99 (0·2%)	57 (0·4%)	691 (0·1%)
Death from stroke					
	0	1 150 152 (99·9%)	48 625 (99·6%)	13 396 (99·3%)	1 198 777 (99·9%)
	1	1195 (0·1%)	181 (0·4%)	101 (0·7%)	1376 (0·1%)
Death from COVID-19					
	0	1 137 876 (98·8%)	45 781 (93·8%)	11 678 (86·5%)	1 183 657 (98·6%)
	1	13 471 (1·2%)	3025 (6·2%)	1819 (13·5%)	16 496 (1·4%)
Hospitalisation for COVID-19					
	0	1 088 788 (94·6%)	38 383 (78·6%)	8460 (62·7%)	1 127 171 (93·9%)
	1	62 559 (5·4%)	10 423 (21·4%)	5037 (37·3%)	72 982 (6·1%)
ICU admission for COVID-19					
	0	1 144 510 (99·4%)	47 747 (97·8%)	13 004 (96·3%)	1 192 257 (99·3%)
	1	6837 (0·6%)	1059 (2·2%)	493 (3·7%)	7896 (0·7%)

GC, glucocorticoid; ICU, intensive care unit. *One or more prescriptions of oral GCs within 12 months before the COVID-19 infection date. †Two or more prescriptions and prednisolone ≥750 mg or equivalent within 6 months before COVID-19 infection date. Note this group is a subset of any prior exposure.

For peer review only

Supplementary Table S4: **Baseline characteristics of individuals with COVID-19 infection in the Swedish population during the period 2015 to COVID-19 infection date (comorbidities) or 1 year before COVID-19 infection date (medications) until Nov 30, 2021, stratified by level of exposure to oral glucocorticoids (high exposure in the 6 months before infection or any exposure 12 months before infection), with standardized mean differences between exposed and 1:2 propensity score matched non-exposed**

	Any prior GC exposure* (n=47 737)	Non-exposed matched to any prior exposure (n=91 878)	SMD (any prior *vs* matched non-exposed)	High GC exposure† (n=12 943)	Non-exposed matched to the high GC exposed (n=23 898)	SMD (high *vs* matched non-exposed)
Age (years)	50·83 (21·06)	49·95 (20·83)	0·042	61·60 (19·69)	60·17 (19·63)	0·073
Age category (years)			0·047			0·081
0–9	930 (1·9)	1840 (2·0)		91 (0·7)	174 (0·7)	
10–19	3056 (6·4)	6089 (6·6)		284 (2·2)	556 (2·3)	
20–29	4491 (9·4)	8982 (9·8)		567 (4·4)	1128 (4·7)	
30–39	5879 (12·3)	11 699 (12·7)		925 (7·1)	1834 (7·7)	
40–49	8041 (16·8)	15 882 (17·3)		1514 (11·7)	2940 (12·3)	
50–59	8953 (18·8)	17 620 (19·2)		2237 (17·3)	4481 (18·8)	
60–69	6221 (13·0)	11 784 (12·8)		2146 (16·6)	4095 (17·1)	
70–79	5321 (11·1)	9652 (10·5)		2515 (19·4)	4403 (18·4)	
80–89	3719 (7·8)	6374 (6·9)		2021 (15·6)	3243 (13·6)	
≥90	1126 (2·4)	1956 (2·1)		643 (5·0)	1044 (4·4)	
Sex			0·002			0·021
Male	20 132 (42·2)	38 844 (42·3)		5480 (42·3)	10 368 (43·4)	
Female	27 605 (57·8)	53 034 (57·7)		7463 (57·7)	13 530 (56·6)	
Birth country			0·087			0·137
Sweden	37 607 (78·8)	69 317 (75·4)		10 450 (80·7)	18 124 (75·8)	
Nordic	1223 (2·6)	2201 (2·4)		456 (3·5)	756 (3·2)	
Europe except Nordics	3084 (6·5)	6953 (7·6)		744 (5·7)	1768 (7·4)	
Other	5823 (12·2)	13 407 (14·6)		1293 (10·0)	3250 (13·6)	
Employment status			0·036			0·053
Employed	31 069 (65·1)	61 146 (66·6)		6398 (49·4)	12 421 (52·0)	
Unemployed	14 422 (30·2)	26 260 (28·6)		6337 (49·0)	11 073 (46·3)	
Unknown	2246 (4·7)	4472 (4·9)		208 (1·6)	404 (1·7)	
Education level			0·018			0·018
Primary	9397 (19·7)	17 445 (19·0)		3408 (26·3)	6114 (25·6)	
Secondary	19 470 (40·8)	37 752 (41·1)		5466 (42·2)	10 160 (42·5)	
Higher	15 699 (32·9)	30 487 (33·2)		3628 (28·0)	6819 (28·5)	
Unknown	3171 (6·6)	6194 (6·7)		441 (3·4)	805 (3·4)	
Comorbidities						
Pulmonary embolism	1536 (3·2)	2643 (2·9)	0·020	768 (5·9)	1243 (5·2)	0·032
Stroke	1606 (3·4)	2780 (3·0)	0·019	793 (6·1)	1366 (5·7)	0·017
Deep vein thrombosis	278 (0·6)	542 (0·6)	0·001	87 (0·7)	154 (0·6)	0·003
Hypertension	12 570 (26·3)	23 230 (25·3)	0·024	5753 (44·4)	10 133 (42·4)	0·041
Ischaemic heart disease	4054 (8·5)	7209 (7·8)	0·024	1907 (14·7)	3201 (13·4)	0·039
Overall CV disease	18 019 (37·7)	31 289 (34·1)	0·077	7751 (59·9)	12 982 (54·3)	0·113
Heart failure	3989 (8·4)	7060 (7·7)	0·025	2033 (15·7)	3442 (14·4)	0·036
Asthma	7625 (16·0)	4507 (4·9)	0·368	1935 (15·0)	1591 (6·7)	0·270
COPD	3403 (7·1)	6444 (7·0)	0·004	1500 (11·6)	2769 (11·6)	0·001

For peer review only

Emphysema	354 (0·7)	404 (0·4)	0·039	185 (1·4)	190 (0·8)	0·061
Diabetes mellitus	5098 (10·7)	9671 (10·5)	0·005	2431 (18·8)	4336 (18·1)	0·016
Rheumatic disease	5523 (11·6)	7075 (7·7)	0·131	3557 (27·5)	4843 (20·3)	0·170
Cancer	7171 (15·0)	13 624 (14·8)	0·005	3219 (24·9)	6235 (26·1)	0·028
Prescribed medications						
Inhaled corticosteroids	17 363 (36·4)	14 069 (15·3)	0·496	4061 (31·4)	4602 (19·3)	0·281
Oral anticoagulants	8017 (16·8)	14 677 (16·0)	0·022	3617 (27·9)	6288 (26·3)	0·037
Statins	10 571 (22·1)	19 209 (20·9)	0·030	4240 (32·8)	7728 (32·3)	0·009
ACEIs	6601 (13·8)	12 781 (13·9)	0·002	2658 (20·5)	5130 (21·5)	0·023
Opioids	12 026 (25·2)	14 517 (15·8)	0·234	4284 (33·1)	5064 (21·2)	0·270

Data are n (%) or mean (SD). ACEI, angiotensin-converting enzyme inhibitor; COPD, chronic obstructive pulmonary disease; CV, cardiovascular; GC, glucocorticoid; SMD, standardised mean difference.
*One or more prescriptions of oral GCs within 12 months before the COVID-19 infection date. †Two or more prescriptions and prednisolone ≥750 mg or equivalent within 6 months before COVID-19 infection date. Note this group is a subset of any prior exposure.

For peer review only

Supplementary Table S5: **Outcome events in propensity score matched cohorts of individuals with COVID-19 infection in the Swedish population during the period 2015 to COVID-19 infection date until Nov 30, 2021 stratified by level of exposure to oral glucocorticoids (non-exposed, high exposure in the 6 months before infection, or any exposure 12 months before infection and overall)**

	No=0/Yes=1	Any GC exposure and matched non-exposed			High GC exposure and matched non-exposed		
		Any GC exposure* (n=47 737)	Matched non-exposed (n=91 878)	Total any exposure and matched non-exposed (n=139 615)	High GC exposure† (n=12 943)	Matched non-exposed (n=23 898)	Total high exposure and matched non-exposed (n=36 841)
All-cause mortality							
	0	44 573 (93·4%)	87 973 (95·7%)	132 546 (94·9%)	11 054 (85·4%)	22 024 (92·2%)	33 078 (89·8%)
	1	3164 (6·6%)	3905 (4·3%)	7069 (5·1%)	1889 (14·6%)	1874 (7·8%)	3763 (10·2%)
Death from pulmonary embolism							
	0	47 598 (99·7%)	91 691 (99·8%)	139 289 (99·8%)	12 867 (99·4%)	23 804 (99·6%)	36 671 (99·5%)
	1	139 (0·3%)	187 (0·2%)	326 (0·2%)	76 (0·6%)	94 (0·4%)	170 (0·5%)
Death from sepsis							
	0	47 467 (99·4%)	91 627 (99·7%)	139 094 (99·6%)	12 799 (98·9%)	23 786 (99·5%)	36 585 (99·3%)
	1	270 (0·6%)	251 (0·3%)	521 (0·4%)	144 (1·1%)	112 (0·5%)	256 (0·7%)
Death from myocardial infarction							
	0	47 646 (99·8%)	91 718 (99·8%)	139 364 (99·8%)	12 889 (99·6%)	23 830 (99·7%)	36 719 (99·7%)
	1	91 (0·2%)	160 (0·2%)	251 (0·2%)	54 (0·4%)	68 (0·3%)	122 (0·3%)
Death from stroke							
	0	475 63 (99·6%)	91 592 (99·7%)	139 155 (99·7%)	12 845 (99·2%)	23 752 (99·4%)	36 597 (99·3%)
	1	174 (0·4%)	286 (0·3%)	460 (0·3%)	98 (0·8%)	146 (0·6%)	244 (0·7%)
Death from COVID-19							
	0	44 904 (94·1%)	88 387 (96·2%)	133 291 (95·5%)	11 240 (86·8%)	22 219 (93·0%)	33459 (90·8%)
	1	2833 (5·9%)	3491 (3·8%)	6324 (4·5%)	1703 (13·2%)	1679 (7·0%)	3382 (9·2%)
Hospitalisation for COVID-19							
	0	37 981 (79·6%)	77 727 (84·6%)	115 708 (82·9%)	8252 (63·8%)	17 810 (74·5%)	26 062 (70·7%)
	1	9756 (20·4%)	14 151 (15·4%)	23 907 (17·1%)	4691 (36·2%)	6088 (25·5%)	10 779 (29·3%)
ICU admission for COVID-19							
	0	46 736 (97·9%)	90 383 (98·4%)	137 119 (98·2%)	12 470 (96·3%)	23 320 (97·6%)	35 790 (97·1%)
	1	1001 (2·1%)	1495 (1·6%)	2496 (1·8%)	473 (3·7%)	578 (2·4%)	1051 (2·9%)

GC, glucocorticoid; ICU, intensive care unit. *One or more prescriptions of oral GCs within 12 months before the COVID-19 infection date. †Two or more prescriptions and prednisolone ≥750 mg of or equivalent within 6 months before COVID-19 infection date. Note this group is a subset of any prior exposure.

For peer review only

Supplementary Table S6: **Standardised mean differences between the 15 a priori selected covariates of individuals with COVID-19 infections in the Swedish population during the period 2015 to COVID-19 infection date (comorbidities) or 1 year before COVID-19 infection date (medications) in those exposed to oral glucocorticoids compared with those non-exposed before and after propensity score matching, main analysis**

Variable	Any prior GC exposure*		High GC exposure†	
	SMD before PS matching	SMD after PS matching	SMD before PS matching	SMD after PS matching
Age categories	0·672	0·047	1·276	0·081
Sex	0·149	0·002	0·147	0·021
Education	0·300	0·018	0·501	0·018
Employment	0·453	0·036	0·888	0·053
Pulmonary embolism	0·194	0·020	0·310	0·032
Stroke	0·161	0·019	0·282	0·017
Deep vein thrombosis	0·088	0·001	0·098	0·003
Hypertension	0·547	0·024	0·972	0·041
Ischaemic heart disease	0·292	0·024	0·487	0·039
Heart failure	0·341	0·025	0·561	0·036
COPD	0·356	0·004	0·488	0·001
Diabetes mellitus	0·289	0·005	0·510	0·016
Rheumatic disease	0·510	0·131	0·897	0·170
Cancer	0·406	0·005	0·653	0·028
Oral anticoagulants	0·413	0·022	0·689	0·037

COPD, chronic obstructive pulmonary disease; GC, glucocorticoid; PS, propensity score; SMD, standardised mean difference. *One or more prescriptions of oral GCs within 12 months before the COVID-19 infection date. †Two or more prescriptions and a total of prednisolone ≥750 mg or equivalent within 6 months before COVID-19 infection date. Note this group is a subset of any prior exposure.

For peer review only

Supplementary Table S7: **Standardised mean differences between the 15 a priori selected plus added‡ covariates in sensitivity analysis of individuals with COVID-19 infections in the Swedish population during the period 2015 to COVID-19 infection date (comorbidities) or 1 year before COVID-19 infection date (medications) in those exposed to oral glucocorticoids compared with those non-exposed before and after propensity score matching**

Variable	Any prior GC exposure*		High GC exposure†	
	SMD before PS matching	SMD after PS matching	SMD before PS matching	SMD after PS matching
Age categories	0.672	0.052	1.276	0.046
Sex	0.149	0.006	0.147	0.019
Birth country	0.105	0.065	0.199	0.092
Employment status	0.453	0.048	0.888	0.035
Education level	0.300	0.023	0.501	0.017
Pulmonary embolism	0.194	0.034	0.310	0.026
Stroke	0.161	0.026	0.282	0.013
Deep vein thrombosis	0.088	0.005	0.098	0.006
Hypertension	0.547	0.053	0.972	0.045
Ischaemic heart disease	0.292	0.043	0.487	0.027
Heart failure	0.341	0.060	0.561	0.048
COPD	0.357	0.047	0.488	0.006
Diabetes mellitus	0·289	0.068	0·510	0.105
Rheumatic disease	0·510	0.039	0·897	0.021
Cancer	0·406	0.151	0·653	0.185
Oral anticoagulants	0.413	0.046	0.689	0.035
Added covariates‡				
Adrenal insufficiency	0.168	0.031	0.285	0.023
Rheumatoid arthritis	0.344	0.086	0.539	0.078
Chronic lower respiratory diseases	0.559	0.009	0.618	0.043
Other respiratory diseases principally affecting the interstitium	0.195	0.037	0.308	0.031
Non-infective enteritis and colitis,	0.255	0.020	0.322	0.056
Dermatitis and eczema	0.262	0.016	0.287	0.023
Inflammatory polyarthropathies	0.471	0.072	0.718	0.049
Systemic connective tissue disorders (except rheumatoid arthritis ICD-coded M31 and M35)	0.234	0.044	0.362	0.029
Glomerular diseases,	0.160	0.048	0.247	0.068
Chronic liver disease	0.140	0.070	0.213	0.009
Organ or tissue transplant	0.234	0.107	0.358	0.111
Alcoholism and other substance abuse	0.118	0.014	0.156	0.004
Opioids	0.424	0.016	0.601	0.008
Inhaled corticosteroids	0.645	0.059	0.535	0.071
Statins	0.389	0.027	0.640	0.014
ACEIs	0.283	0.016	0.460	0.001

COPD, chronic obstructive pulmonary disease; GC, glucocorticoid; PS, propensity score; SMD, standardised mean difference; ICD, 10th edition of the International Classification of Diseases; ACEIs, angiotensin converting enzyme inhibitors. *One or more prescriptions of oral GCs within 12 months before the COVID-19 infection date. †Two or more prescriptions and a total of prednisolone ≥750 mg or equivalent within 6 months before COVID-19 infection date. Note this group is a subset of any prior exposure. ‡ 16 other covariates of interest for oral GC users.

For peer review only

Supplementary Table S8: **Unadjusted, as well as adjusted from main and sensitivity analysis of the risk of various causes of death and all-cause mortality between COVID-19 patients who had any or high prior exposure to oral glucocorticoids and non-exposed individuals, hazard ratios from Cox regression analyses and propensity score matched analysis with 95% confidence intervals.**

**Outcomes and method**	**Any prior GC exposure***		**High GC exposure†**	
	**HR (95% CI)**	**No. of events**	**HR (95% CI)**	**No. of events**
All-cause mortality				
Unadjusted Cox regression	5·46 (5·26–5·67)	18 228	12·33 (11·77–12·92)	16 873
Adjusted Cox regression‡	1·58 (1·52–1·65)	18 228	1·98 (1·87–2·09)	16 873
Cox with extended adjustments‡‡ [Sensitivity analysis]	1.55 [1.48-1.61]	18 228	1.85 [1.75-1.96]	16 873
PS matching¶	1·47 (1·40–1·54)	7 069	1·83 (1·71–1·95)	3 763
PS matching with additional covariates¶¶[Sensitivity analysis]	1.53 [1.46-1.61]	6 673	1.67 [1.57-1.79]	3 886
Death from pulmonary embolism				
Unadjusted Cox regression	6·29 (5·26–7·52)	730	12·68 (10·04–16·03)	658
Adjusted Cox regression‡	1·33 (1·11–1·59)	730	1·37 (1·09–1·74)	658
Cox with extended adjustments‡‡ [Sensitivity analysis]	1.38 [1.16-1.65]	730	1.35 [1.07-1.71]	658
PS matching¶	1·32 (1·05–1·65)	326	1·52 (1·12–2·06)	170
PS matching with additional covariates¶¶[Sensitivity analysis]	1.54 [1.21-1.95]	290	1.52 [1.11-2.09]	160
Death from sepsis				
Unadjusted Cox regression	8·67 (7·57–9·94)	1 057	18·72 (15·79–22·19)	933
Adjusted Cox regression‡	2·35 (2·01–2·74)	1 057	2·90 (2·37–3·54)	933
Cox with extended adjustments‡‡ [Sensitivity analysis]	2.10 [1.79-2.47]	1 057	2.43 [1.96-3.00]	933
PS matching¶	1·92 (1·61–2·29)	521	2·40 (1·86–3·09)	256
PS matching with additional covariates¶¶[Sensitivity analysis]	1.87 [1.56-2.25]	492	1.90 [1.49-2.42]	276
Death from stroke				
Unadjusted Cox regression	3·47 (2·96–4·05)	1 376	7·22 (5·89–8·85)	1 296
Adjusted Cox regression‡	1·16 (0·98–1·37)	1 376	1·33 (1·05–1·67)	1 296
Cox with extended adjustments‡‡ [Sensitivity analysis]	1.14 [0.96-1.36]	1 376	1.23 [0.97-1.56]	1 296
PS matching¶	1·08 (0·89–1·31)	460	1·23 (0·94–1·59)	244
PS matching with additional covariates¶¶[Sensitivity analysis]	1.01 [0.83-1.24]	443	0.99 [0.76-1.28]	263
Death from myocardial infarction				
Unadjusted Cox regression	3·84 (3·10–4·75)	691	8·23 (6·27–10·82)	649
Adjusted Cox regression‡	1·04 (0·82–1·31)	691	1·36 (1·00–1·86)	649
Cox with extended adjustments‡‡ [Sensitivity analysis]	1.08 [0.85-1.38]	691	1.41 [1.02-1.94]	649
PS matching¶	1·02 (0·79–1·33)	251	1·48 (1·03–2·13)	122
PS matching with additional covariates¶¶[Sensitivity analysis]	1.05 [0.80-1.37]	239	1.24 [0.86-1.78]	125
Death from COVID–19				
Unadjusted Cox regression	5·43 (5·22–5·65)	16 496	12·34 (11·75–12·96)	15 290
Adjusted Cox regression‡	1·57 (1·50–1·64)	16 496	1·96 (1·85–2·08)	15 290
Cox with extended adjustments‡‡ [Sensitivity analysis]	1.53 [1.46-1.60]	16 496	1.83 [1.73-1.94]	15 290
PS matching¶	1·48 (1·41–1·56)	6 324	1·84 (1·72–1·97)	3 382
PS matching with additional covariates¶¶[Sensitivity analysis]	1.54 [1.46-1.62]	5 989	1.69 [1.58-1.81]	3 489
Secondary outcomes				
Hospitalisation for COVID–19				
Unadjusted Cox regression	4·34 (4·25–4·43)	72 982	8·52 (8·28–8·77)	67 596
Adjusted Cox regression‡	1·42 (1·39–1·45)	72 982	1·51 (1·46–1·57)	67 596
Cox with extended adjustments‡‡ [Sensitivity analysis]	1.20 [1.17-1.23]	72 982	1.25 [1.20-1.29]	67 596
PS matching¶	1·28 (1·25–1·31)	23 907	1·44 (1·38–1·49)	10 779

For peer review only

PS matching with additional covariates¶¶[Sensitivity analysis]	1.19 [1.16-1.22]	23 814	1.29 [1.24-1.34]	11 037
ICU admission for COVID–19				
Unadjusted Cox regression	3·72 (3·49–3·97)	7 896	6·41 (5·85–7·03)	7 330
Adjusted Cox regression‡	1·33 (1·24–1·43)	7 896	1·40 (1·26–1·55)	7 330
Cox with extended adjustments‡‡ [Sensitivity analysis]	0.90 [0.84-0.97]	7 896	0.80 [0.72-0.89]	7 330
PS matching¶	1·24 (1·15–1·35)	2 496	1·52 (1·34–1·72)	1 051
PS matching with additional covariates¶¶[Sensitivity analysis]	0.99 [0.92-1.07]	2 703	1.00 [0.89-1.13]	1 206

CI, confidence interval; GC, glucocorticoid; HR, hazard ratio; PS, propensity score. *One or more prescriptions of oral GCs within 12 months before the COVID-19 infection date. †Two or more prescriptions and prednisolone ≥750 mg or equivalent within 6 months before COVID-19 infection date. Note high exposure is a subset of any prior exposure. ‡Adjusted for initial a priori selected covariates: age, sex, education, employment, and previous medical history (diabetes, deep vein thrombosis, pulmonary embolism, hypertension, stroke, ischaemic heart disease, heart failure, cancer, chronic obstructive pulmonary disease, rheumatologic conditions, and oral anticoagulation therapy. ‡‡ Adjusted for additional covariates on top of initial (initial covariates + adrenal insufficiency, rheumatoid arthritis, chronic lower respiratory diseases, other respiratory diseases principally affecting the interstitium, non-infective enteritis and colitis, dermatitis and eczema, inflammatory polyarthropathies, systemic connective tissue disorders (except rheumatoid arthritis), glomerular diseases, organ or tissue transplant , chronic liver disease, medications: inhaled corticosteroids, statins, aceis, opioids, alcoholism and other substance abuse). ¶1:2 matched on a PS score based on 15 initial a priori selected covariates: age, sex, education, employment, and previous medical history (diabetes, deep vein thrombosis, pulmonary embolism, hypertension, stroke, ischaemic heart disease, heart failure, cancer, chronic obstructive pulmonary disease, rheumatologic conditions, oral anticoagulation therapy). ¶¶ 1:2 matched on a PS score based on 31 covariates: initial 15 + adrenal insufficiency, rheumatoid arthritis, chronic lower respiratory diseases, other respiratory diseases principally affecting the interstitium, non-infective enteritis and colitis, dermatitis and eczema, inflammatory polyarthropathies, systemic connective tissue disorders (except rheumatoid arthritis), glomerular diseases, organ or tissue transplant , chronic liver disease, medications: inhaled corticosteroids, statins, aceis, opioids, alcoholism and other substance abuse.

For peer review only

Supplementary Figure S1: **Propensity score balance before (a, c) and after (b, d) performing propensity score matching on all 15 covariates for individuals with COVID-19 infections in the Swedish population between 2015 and the COVID-19 infection date comparing those exposed to oral glucocorticoids (pink) with those who were non-exposed (blue) in those with any (a, b) or high glucocorticoid exposure (c, d)**

GC, glucocorticoid; PS, propensity score. Any prior GC exposure equals ≥1 prescription of oral GC within 12 months before the COVID-19 infection date. High GC exposure equals ≥2 prescriptions with a total of ≥750 mg of prednisolone or equivalent. The latter group is a subset of any prior exposure. Note: The figure includes PS density plots and histograms illustrating the distribution of PS values. Prior to matching, the PS density plots and histograms represent the distribution of PS values for both exposed and non-exposed individuals. Subsequently, a 1:2 matching scheme was employed using a caliper of 0.2 where each exposed individual was paired with two non-exposed individuals based on their similarity in PS values. The PS density plots and histograms after matching reflect the distribution of PS values for the matched groups, demonstrating the achieved balance in the covariates. The 15 covariates used in the logistic model to estimate the propensity score can be found in Supplementary Table S7.

(a) Any GC exposure before PS matching

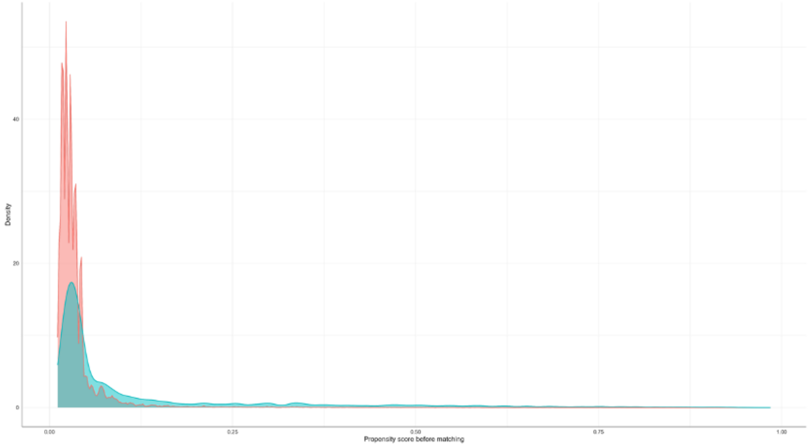

(b) Any GC exposure after PS matching

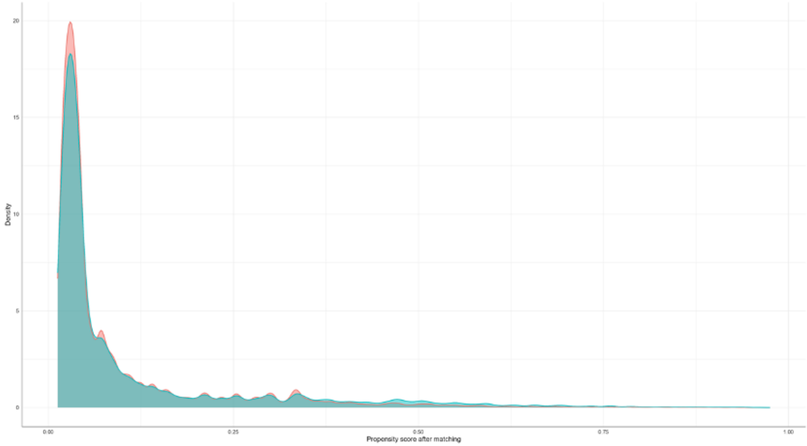

(c) High GC exposure before PS matching

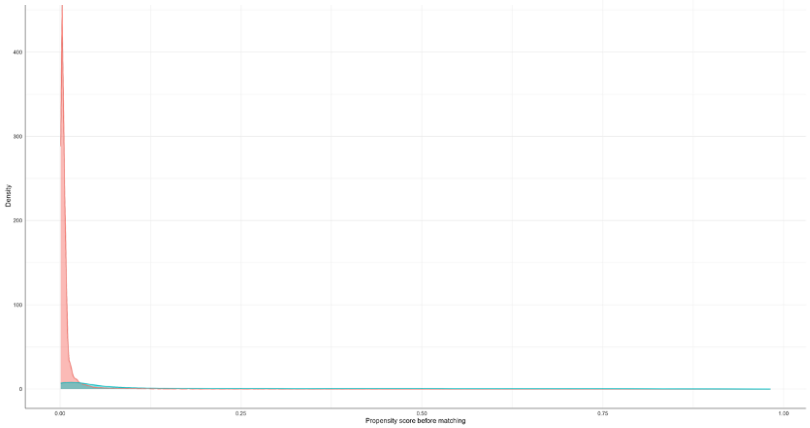

(d) High GC exposure after PS matching

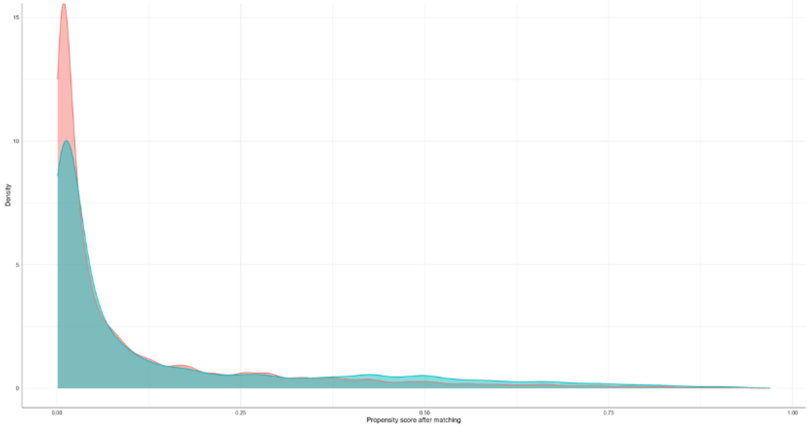

Supplementary Figure S2: Standardised mean differences between the covariates of individuals with COVID-19 infections in the Swedish population during the period 2015 to COVID-19 infection date (comorbidities) or 1 year before COVID-19 infection date (medications) in the exposed to oral glucocorticoids compared with non-exposed before (red circles) and after (blue triangles) propensity score matching among those with (a) any or (b) high glucocorticoid exposure. COPD, chronic obstructive pulmonary disease; GC, glucocorticoid. Any prior exposure equals ≥1 prescription of oral glucocorticoids within 12 months before the COVID-19 infection date. High exposure equals ≥2 prescriptions of oral glucocorticoids with a total of ≥750 mg of prednisolone or equivalent within 6 months before COVID-19 infection date. Note the latter group is a subset of any prior exposure.

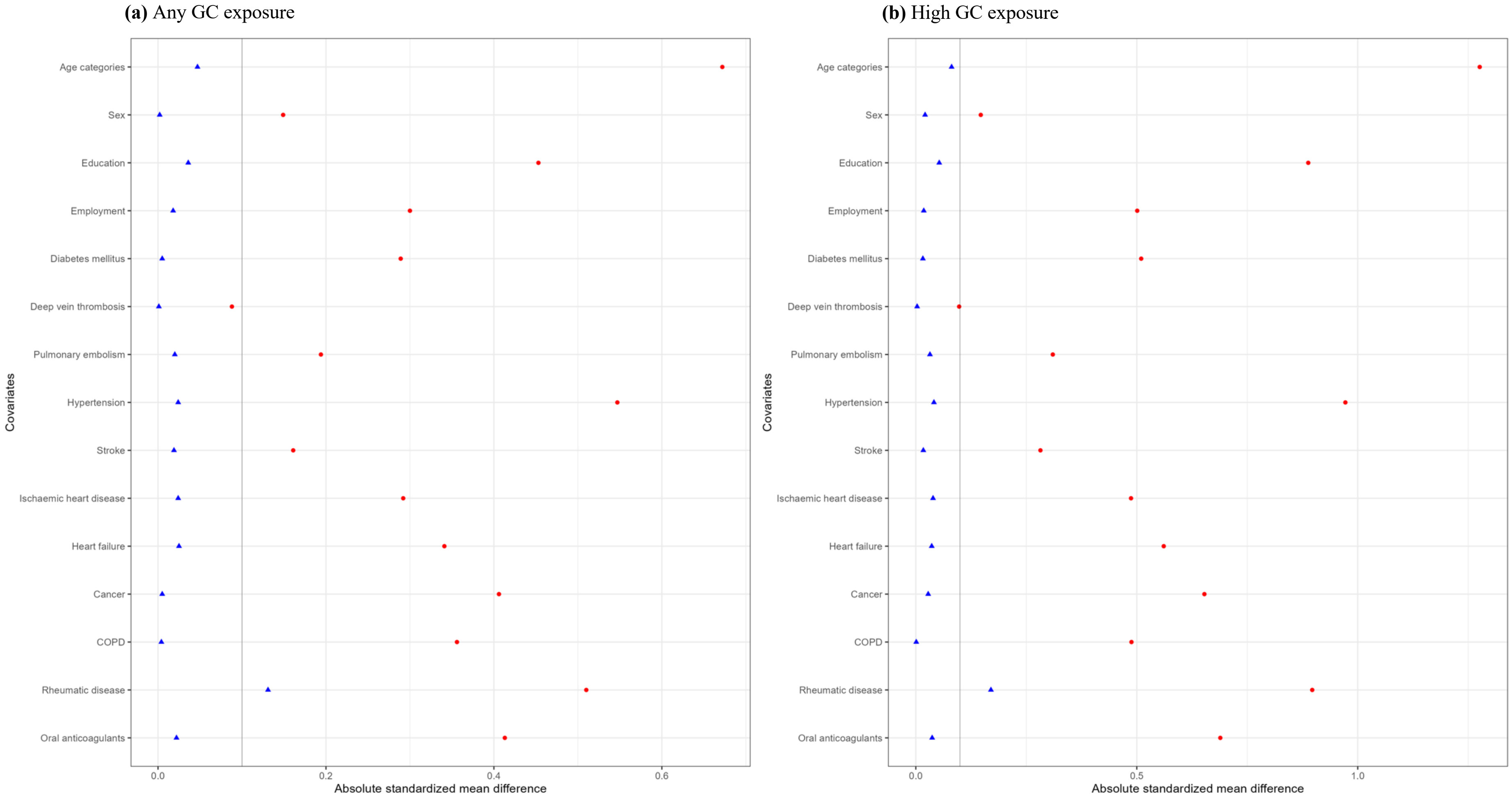

STROBE Statement—Checklist of items that should be included in reports of ***cohort studies***

	Item No	Recommendation
**Title and abstract**	1	(*a*) Indicate the study's design with a commonly used term in the title or the abstract **Page 1**
		(*b*) Provide in the abstract an informative and balanced summary of what was done and what was found **Page 5-6**
**Introduction**		
Background/rationale	2	Explain the scientific background and rationale for the investigation being reported **Page 7-9**
Objectives	3	State specific objectives, including any prespecified hypotheses **Page 9**
**Methods**		
Study design	4	Present key elements of study design early in the paper **Page 9-12**
Setting	5	Describe the setting, locations, and relevant dates, including periods of recruitment, exposure, follow-up, and data collection **Page 9-12**
Participants	6	(*a*) Give the eligibility criteria, and the sources and methods of selection of participants. Describe methods of follow-up **Page 9-12**
		(*b*) For matched studies, give matching criteria and number of exposed and unexposed **Page 12-15**
Variables	7	Clearly define all outcomes, exposures, predictors, potential confounders, and effect modifiers. Give diagnostic criteria, if applicable **Page 10-12**
Data sources/ measurement	8*	For each variable of interest, give sources of data and details of methods of assessment (measurement). Describe comparability of assessment methods if there is more than one group **Page 9-12**
Bias	9	Describe any efforts to address potential sources of bias **Page 12**
Study size	10	Explain how the study size was arrived at **Page 9**
Quantitative variables	11	Explain how quantitative variables were handled in the analyses. If applicable, describe which groupings were chosen and why **Page 12**
Statistical methods	12	(*a*) Describe all statistical methods, including those used to control for confounding **Page 12**
		(*b*) Describe any methods used to examine subgroups and interactions
		(*c*) Explain how missing data were addressed **No missing data reported**
		(*d*) If applicable, explain how loss to follow-up was addressed
		(*e*) Describe any sensitivity analyses **Page 14-15**
**Results**		
Participants	13*	(a) Report numbers of individuals at each stage of study—eg numbers potentially eligible, examined for eligibility, confirmed eligible, included in the study, completing follow-up, and analysed **Page 13**
		(b) Give reasons for non-participation at each stage
		(c) Consider use of a flow diagram
Descriptive data	14*	(a) Give characteristics of study participants (eg demographic, clinical, social) and information on exposures and potential confounders **Table 1**
		(b) Indicate number of participants with missing data for each variable of interest **No missing data reported**
		(c) Summarise follow-up time (eg, average and total amount) **Page 10-11**
Outcome data	15*	Report numbers of outcome events or summary measures over time **Supplementary table S5 and S6**
Main results	16	(*a*) Give unadjusted estimates and, if applicable, confounder-adjusted estimates and

For peer review only

		their precision (eg, 95% confidence interval). Make clear which confounders were adjusted for and why they were included **Supplementary table S6**
		(*b*) Report category boundaries when continuous variables were categorized **Supplementary Table S2**
		(*c*) If relevant, consider translating estimates of relative risk into absolute risk for a meaningful time period
Other analyses	17	Report other analyses done—eg analyses of subgroups and interactions, and sensitivity analyses **Page 14-15**
**Discussion**		
Key results	18	Summarise key results with reference to study objectives **Page 15**
Limitations	19	Discuss limitations of the study, taking into account sources of potential bias or imprecision. Discuss both direction and magnitude of any potential bias **Page 17-18**
Interpretation	20	Give a cautious overall interpretation of results considering objectives, limitations, multiplicity of analyses, results from similar studies, and other relevant evidence **Page 15-19**
Generalisability	21	Discuss the generalisability (external validity) of the study results **Page 18**
**Other information**		
Funding	22	Give the source of funding and the role of the funders for the present study and, if applicable, for the original study on which the present article is based **Page 5**

*Give information separately for exposed and unexposed groups.

Note: An Explanation and Elaboration article discusses each checklist item and gives methodological background and published examples of transparent reporting. The STROBE checklist is best used in conjunction with this article (freely available on the Web sites of PLoS Medicine at http://www.plosmedicine.org/, Annals of Internal Medicine at http://www.annals.org/, and Epidemiology at http://www.epidem.com/). Information on the STROBE Initiative is available at http://www.strobe-statement.org.

For peer review only

For peer review only - http://bmjopen.bmj.com/site/about/guidelines.xhtml
